# Long promoter sequences form higher-order G-quadruplexes: an integrative structural biology study of *c-Myc*, *k-Ras* and *c-Kit* promoter sequences

**DOI:** 10.1093/nar/gkac182

**Published:** 2022-03-23

**Authors:** Robert C Monsen, Lynn W DeLeeuw, William L Dean, Robert D Gray, Srinivas Chakravarthy, Jesse B Hopkins, Jonathan B Chaires, John O Trent

**Affiliations:** UofL Health Brown Cancer Center, University of Louisville, Louisville, KY 40202, USA; UofL Health Brown Cancer Center, University of Louisville, Louisville, KY 40202, USA; UofL Health Brown Cancer Center, University of Louisville, Louisville, KY 40202, USA; UofL Health Brown Cancer Center, University of Louisville, Louisville, KY 40202, USA; The Biophysics Collaborative Access Team (BioCAT), Department of Biological, Chemical, and Physical Sciences, Illinois Institute of Technology, Chicago, IL 60616, USA; The Biophysics Collaborative Access Team (BioCAT), Department of Biological, Chemical, and Physical Sciences, Illinois Institute of Technology, Chicago, IL 60616, USA; UofL Health Brown Cancer Center, University of Louisville, Louisville, KY 40202, USA; Department of Medicine, University of Louisville, Louisville, KY 40202, USA; Department of Biochemistry and Molecular Genetics, University of Louisville, Louisville, KY 40202, USA; UofL Health Brown Cancer Center, University of Louisville, Louisville, KY 40202, USA; Department of Medicine, University of Louisville, Louisville, KY 40202, USA; Department of Biochemistry and Molecular Genetics, University of Louisville, Louisville, KY 40202, USA

## Abstract

We report on higher-order G-quadruplex structures adopted by long promoter sequences obtained by an iterative integrated structural biology approach. Our approach uses quantitative biophysical tools (analytical ultracentrifugation, small-angle X-ray scattering, and circular dichroism spectroscopy) combined with modeling and molecular dynamics simulations, to derive self-consistent structural models. The formal resolution of our approach is 18 angstroms, but in some cases structural features of only a few nucleotides can be discerned. We report here five structures of long (34–70 nt) wild-type sequences selected from three cancer-related promoters: *c-Myc*, *c-Kit* and *k-Ras*. Each sequence studied has a unique structure. Three sequences form structures with two contiguous, stacked, G-quadruplex units. One longer sequence from *c-Myc* forms a structure with three contiguous stacked quadruplexes. A longer c-Kit sequence forms a quadruplex-hairpin structure. Each structure exhibits interfacial regions between stacked quadruplexes or novel loop geometries that are possible druggable targets. We also report methodological advances in our integrated structural biology approach, which now includes quantitative CD for counting stacked G-tetrads, DNaseI cleavage for hairpin detection and SAXS model refinement. Our results suggest that higher-order quadruplex assemblies may be a common feature within the genome, rather than simple single quadruplex structures.

## INTRODUCTION

DNA and RNA sequences with four runs of typically three consecutive guanine residues separated by 1–7 nucleotides may fold into stable four-stranded structures called G-quadruplexes (G4s) under defined solution conditions (1,2). In G4s, four G residues from different runs assemble to form a planar G-quartet that is stabilized by Hoogsteen hydrogen bonding. These quartets stack to form the G4 core, which is further stabilized by coordination of a monovalent cation to the guanine O6 atoms (reviewed in (3)).

Bioinformatic analysis of a variety of genomes has revealed that oncogenic promoter regions frequently contain tracts of G residues that could potentially fold into quadruplex structures and may regulate adjacent gene transcription (4–6). These findings have been validated by direct *in vitro* and ChIP-sequencing studies (7,8). More recently studies have revealed that promoter quadruplex formation is linked to binding of transcription factors (9) and epigenetic regulation of promoter function in live cells (10). Numerous studies have now validated the concept that ligand-induced G4 stabilization can modulate oncogene expression (reviewed in ref. (5)). For example, the c-Myc protein is aberrantly overexpressed in >80% of solid tumors. The *c-Myc* promoter region (NHEIII) contains a potential G4-forming sequence of 27 nucleotides (Pu-27) that contains two G_3_ and two G_4_ tracts. Ligand stabilization of the c-Myc promoter G4 decreases production of c-Myc transcripts in cultured cells (11).

To date, most promoter G4 drug discovery efforts have focused on targeting features of the structures of short G-rich sequences that are amenable to characterization by traditional structural biology methods, such as NMR or X-ray crystallography. These sequences are often short (<33 nt), heavily modified (e.g. mutations or deletions), and potentially removed from their biological context (e.g. arbitrarily truncated without consideration of adjacent G-tracts), see Supplementary Table S1 (12–43). Some examples include *c-Myc*(44), *c-Kit* (45), *k-Ras* (46) and *hTERT* (47). It is apparent from the dearth of clinically useful G4 ligands produced by structure-based design that using these simple G4 structures may not be as relevant as drug targets as proposed (48). A possible reason for this limited success is that these ‘well-behaved’ G4s have a paltry repertoire of druggable features. All share a common dominant drug binding site, the terminal G-quartet face. Targeting the G-quartet face often results in selection of planar poly- and hetero-cyclic aromatic compounds that lack optimal drug-like properties, and that bind with high affinity, but little selectivity, to the G-tetrad face (48–50). New avenues for selectively targeting promoter G4s are needed. We hypothesize that longer wild-type promoter sequences can form more complex higher-order structures that might be biologically relevant, and which might contain a richer repertoire of druggable features. These structures might include multiple quadruplexes stacked on one another, or multiple quadruplexes linked by, or including, other secondary structural elements like hairpins. The size of such assemblies is technically challenging for the NMR or X-ray diffraction methods commonly used to determine G-quadruplex structures.

Recent structural studies show that higher-order G4 assemblies exist *in vitro*, and that these more complex structures contain unique binding sites for drug targeting (51–55). It is well established that parallel G4s can stack at the 5′ and 3′ tetrad interfaces (56,57). This arrangement is favorable in the packed conditions of the cell and could be an important regulatory mechanism (58), as these unique structures would provide selective recognition by proteins (59). However, extended G-rich sequences are difficult to study. Often, the number and disposition of G-tracts in promoters suggest the possibility of formation G4 structures with more than the canonical three G-tetrad stack and loop lengths much greater than the traditional 1–4 nucleotides (60). In addition, the presence of multiple G runs can result in formation of G-vacancies or different ‘G-register exchange’ isomers in which different pairings of G residues form a stack (6,61). Such sequence and structural variants, while making structural determination difficult (as there is an ensemble of configurations and potentially topologies), may have biological advantages such as providing an extra G that can substitute for an oxidatively damaged member of a quartet (the ‘spare tire’ hypothesis (62)) or by contributing to the conformational entropy of the folded states, thereby enhancing the probability of G4 formation (61). Further difficulties arise in situations where thermodynamically or kinetically equivalent competing secondary structures exist (22). This plethora of secondary structural possibilities sets the stage for the coexistence of mixtures of G4 topologies. These ensembles of conformers manifest themselves by ill-defined NMR spectra as well as multiple species by SEC, AUC, or electrophoretic experiments (52,60,63–65). Thus, it has been impossible to obtain high-resolution structures of native sequences without resorting to manipulative truncations and mutations to stabilize or create a single conformer at the expense of others (47).

Simple G-quadruplexes from short sequences within promoters have been shown by NMR and X-ray crystallography to adopt a variety of topologies including parallel (or ‘propeller’) and antiparallel (‘chair’, ‘basket’ or ‘hybrid’) structures. The parallel conformation facilitates G4 stacking because by necessity the loops project away from the tetrad faces (hence ‘propeller loops’) which allows charge balancing of sugar phosphate backbone repulsion and counterion binding at stacking interfaces (57,66). G4s can also accommodate large loops (>7 nt) (67,68), and when these loops form duplexes they can be stabilizing (69). In their natural context, promoter G4 sequences are flanked at their termini by several nucleotides. It was recently revealed that 5′-flanking bases tend to favor a parallel topology (70). Indeed, nearly all of the high-resolution promoter-derived quadruplex structures flanked at their 5′ ends are parallel (e.g. promoter/PDB IDs: k-Ras/5I2V (46), c-Kit/2KQG (45), c-Myc/2LBY (71), c-Myc/6NEB (72), c-Myc/1XAV (44), and VEGF/2M27 (73)), although exceptions are noted (an hTERT promoter G4 with a 5′-A flanking base co-exists as parallel and hybrid 3 + 1 (47)). We have observed this general trend for parallel preference by exhaustively searching the literature for all reported CD spectra of putative promoter G4s (Supplementary Table S1) (12–43). The *in vivo* preference for parallel conformations in promoter G4s is supported by recent ChIP-sequencing studies (74).

**Figure 1.**
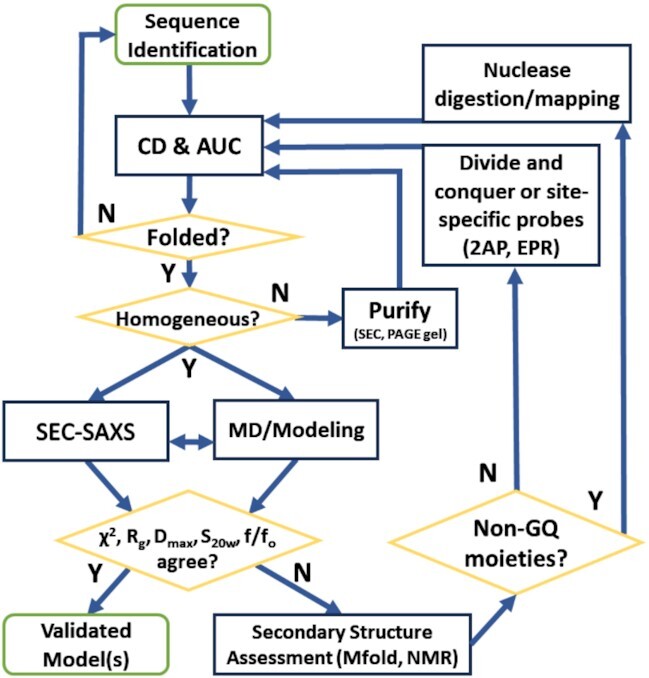
Flow chart of the integrative structural biology approach to model higher-order DNA G-quadruplexes. See Table 1 for more description of each technique or experimental property.

To overcome the limitations of high-resolution structural biology techniques in studying extended G-rich sequences, we have developed an integrative structural biology (ISB) platform (75). The integrative approach (Figure 1 and Table 1) uses every available piece of experimental information about a system, in combination with prior structural information and physical theory, to derive self-consistent molecular models that best explain the collective observables. For DNA, each topology comes with a defining spectroscopic signature (NMR, UV, or CD) (76–79) as well as characteristic hydrodynamic and scattering properties including sedimentation coefficient (*S*_20,w_), frictional ratio (*f/f*_o_) and radius of gyration (*R*_g_) (52,65,80,81). The former informs directly on secondary structural features, while the latter provides coarse grain low- to medium-resolution shape information useful in refining or filtering out inconsistent models. We previously utilized this approach to characterize the human telomerase reverse-transcriptase (*hTERT*) core promoter, a 68-nt sequence with twelve runs of three to five consecutive Gs (52,82). A structure consisting of G4s and WC-hairpin segments was previously proposed based largely on DMS foot-printing experiments (83). Using our ISB approach, however, we demonstrated that such a model was inconsistent with a battery of biophysical measurements, and that the most probable structure for the *hTERT* core promoter was one with three-stacked G4 units, each in a parallel topology (52).

**Table 1.** Table of experimental techniques used in the integrative approach and corresponding qualitative and quantitative information gained from each type of analysis

Experimental technique (Ref)	Qualitative information	Quantitative information	Model refinement/assessment
CD (78)	Topology	# parallel G-tetrad stacks	
AUC(80)	Foldedness from frictional ratio (*f/f*_o_), oligomeric state(monomer/dimer/oligomer)	Molecular weight (MW), Sedimentation coefficient (*S*_20,w_), translational diffusion coefficient (*D*_t_)	*HYDROPRO(104)* calculated *S*_20,w_
SEC (127)	Oligomeric state (monomer/dimer/oligo/ aggregate)	Stokes radius (*R*_s_) or molecular weight (MW)	
SAXS(102,109)	Foldedness, flexibility, shape	Radius of gyration (*R*_g_), maximum particle dimension (*D*_max_), volume	*CRYSOL(102)* calculated scattering, reduced χ^2^ (Eq. 1 in methods), calculated *R*_g_*, HYDROPRO(104)* calculated volume & *D*_max_, *ab initio* reconstructions (96–99)
^1^H NMR (128,129)	Qualitative assessment of duplex/quadruplex imino proton shifts	Amount of Watson-Crick or Hoogsteen bonds	
Nuclease Digestion (52)	Topological changes (when monitored by CD)	Changes in MW*, S*_20,w_*, f/f*_o_, *R*_g_, *D*_max_	*CRYSOL(102)* calculated scattering, reduced χ^2^ (Eq. 1 in methods), calculated *R*_g_*, HYDROPRO(104)* calculated volume & *D*_max_, *ab initio* reconstructions (96–99)
Site-specific Probes (130,131)	Relative solvent exposure	Residue-residue distances	SAS calculations, distance calculations

Here we hypothesize that, like *hTERT*, other long promoter sequences may preferentially form stacked parallel G4s. The all-parallel tertiary structure has a distinctive CD signature characterized by a maximum at ∼264 nm (79,84,85) and we realized that the amplitude of the molar circular dichroism at this wavelength could be used quantitatively to count the number of stacked quartets. We validated and calibrated this by determining the CD spectra of a series of oligonucleotides dTG_*n*_T (*n* = 3–6) known to form tetrameric, all-parallel G4s in K^+^-containing solutions (86,87). We confirmed that the magnitude of the normalized CD signal at 264 nm for parallel promoters is proportional to the number of stacked G4s. A previous study showed the same relationship for a different set of all-parallel oligonucleotides (88,89). Our calibration curve reveals that the number of stacked parallel G-quartets in an unknown G4 can be determined from its 264 nm CD signal.

We also developed a new independent validation method for the ISB approach by examining the agreement between experimental scattering with theoretical scattering curves calculated from models using the program *CRYSOL*. We measured the solution scattering properties of 14 promoter and artificial G4s (Supplementary Table S4) from the Protein Data Bank (PDB) and compared their measured radii of gyration (*R*_g_) and scattering with that of their theoretical values based on their deposited structures. We found an excellent agreement in both cases and subsequently demonstrate how this analysis can and should be used as an additional tool to assess putative molecular models.

Finally, we added the use of secondary structure prediction for the longer promoter sequences, as localized competing structures become more likely with longer sequences and loop regions. To do this we implemented a simple DNaseI cleavage assay to test these predictions.

We used this improved ISB platform to evaluate the extended promoter sequences identified by *Quadparser* (2) in the *c-Kit, k-Ras* and *c-Myc* promoters (Table 2). Hydrodynamic and scattering data confirm that all higher-order sequences form secondary structures. All sequences are consistent with preferential formation of parallel stacked topologies. *In silico* construction of models refined by CD, AUC and SAXS data reveals that the three 8-tract sequences form highly compact parallel stacked G4s, consistent with the parallel promoter hypothesis. The c-Kit 12-tract sequence, which encompasses its 8-tract counterpart, exhibits a similar compact parallel G4 region with an extended GC duplex hairpin feature, which was confirmed by SAXS and DNaseI cleavage experiments. Lastly, the 12-tract c-Myc sequence predominantly forms a globular parallel stacked structure but has competing hairpin features that obscure its analysis by any singular method. Here we demonstrate how our expanded ISB platform can be used to study even the most recalcitrant higher-order DNA G4 systems, and in doing so reveal novel loop and junctional topologies that might be useful in drug discovery efforts.

**Table 2.** Oligonucleotides used in this study with predicted number of G-tetrad stacks based on CD 264 nm amplitude. See Supplementary Table S2 for sequences used in SAXS Rg analysis

ODN Name	Sequence (5′→ 3′)	# nt	# G-Stacks	ϵ (M^–1^ cm^–1^)	MW (Da) (strand)	CD Δϵ_264_ (M^–1^ cm^–1^)	Predicted # G-stacks
TG3T	TGG GT	5	3	471,500	1534		
TG4T	TGG GGT	6	4	576,200	1863		
TG5T	TGG GGG T	7	5	680,900	2193		
TG6T	TGG GGG GT	8	6	785,600	2522		
1XAV	TGA GGG TGG GTA GGG TGG GTA A	22	3	228,700	6992	190	2.9
2LBY	TAG GGA GGG TAG GGA GGG T	19	3	201,700	6054	206	3.1
2M27	CGG GGC GGG CCT TGG GCG GGG T	22	3	200,400	6905	220	3.2
5I2V	AGG GCG GTG TGG GAA TAG GGA A	22	3	233,100	6970	182	2.8
c-Myc-8	GGG GAG GGT GGG GAG GGT GGG GAA GGT GGG GAG G	34		354,900	10976	361	4.7
c-Myc-12	GGG AAC CCG GGA GGG GCG CTT ATG GGG AGG GTG GGG AGG GTG GGG AAG GTG GGG AGG AGA CTC AGC CGG G	70		702,100	22255	705	8.3
c-Kit-8	GGG CGG GCG CGA GGG AGG GGA GGC GAG GGG CGT GG	38		381,800	12143	317	4.2
c-Kit-12	GGG CGG GCG CGA GGG AGG GGA GGC GAG GGG CGT GGC CGG CGC GCA GAG GGA GGG CGC TGG G	64		626,800	20349	339	4.5
k-Ras-8	GGG AGC GGC TGA GGG CGG TGT GGG AAG AGG GAA GAG GGG GAG G*	43		445,300	13755	276	3.8

*Underlined region of k-Ras-8 overlaps with sequence studied in ref (76).

## MATERIALS AND METHODS

### Bioinformatic analysis

*Quadparser* software was downloaded from the Balasubramanian group (2). The *Homo sapiens* genome (December 2013 GRCh38/hg38) Eukaryotic Promoter Database was used for the to generate the promoter sequences from −499 to +100 and −750 to +100, which were searched separately. We set the search parameters to identify four, eight, and twelve runs of two or three guanines with 1–10 loop residues. The results are given in Supplemental Table S2.

### Oligodeoxynucleotides and G4 formation

Oligos (Table 2) were obtained from Eurofins (Louisville, KY) or IDT (Coralville, IA) as lyophilized, desalted powders. Stock solutions of approximately 1 mM were prepared in Milli-Q H_2_O, warmed for ∼30 min at 50°C to facilitate solubilization, and stored at 4°C. Oligo concentration was estimated from the absorbance at 260 nm determined at either pH 11 or 90°C using their extinction coefficients. Working solutions were prepared by diluting the stock oligo solution to the desired strand concentration in the respective buffer. All extended promoter samples were purified by preparative size-exclusion chromatography (SEC) (Superdex 75 16/600 SEC column, GE Healthcare 28-9893-33, running at 0.5 ml/min, fractions collected every 2 min) and concentrated with Pierce protein concentrators (ThermoFisher, #88515) prior to analysis. Two separate buffers were used throughout: TBAP (tBAP, tetrabutyl ammonium phosphate) and BPEK (potassium phosphate). Buffers contained 1 mM EDTA and had a pH of either 6.8 (TBAP) or 7.2 (BPEK) and were supplemented with varying levels of KCl (25–200 mM) as indicated. Samples were annealed by heating for 10 min. in 1 l boiling water followed by slow cooling overnight to room temperature. To ensure complete and rapid formation of d[TG_*n*_T]_4_ tetramers, 1 mM solutions of the dTG_*n*_T oligos were supplemented with 10× BPEK to a final buffer concentration of 1× BPEK followed by overnight incubation at 4°C (90). The samples were not heated. Preliminary experiments revealed that tetramer formation occurred only when folding was initiated by K^+^ addition to oligos at high concentration. Once tetramers were formed at mM oligo concentration, the tetrameric aggregation state was stable after dilution to μM working concentrations as verified by analytical ultracentrifugation (65,80).

### CD spectra

Baseline-corrected, normalized CD spectra were recorded in 1-cm quartz cuvettes with Jasco J710 or J810 spectropolarimeters using the protocol outlined by Del Villar (78). DNaseI digestions assays were conducted in 0.5-cm quartz cuvettes with a Jasco J710 as detailed previously (52). In brief, samples at 12 μM were annealed in EDTA-free TBAP buffer with 185 mM KCl, and then mixed with 4× DNaseI reaction buffer (80 mM Tris, 8 mM MgCl_2_, 40 mM KCl, pH 7.2), and MilliQ dH_2_O in a 2:1:1 ratio to achieve a final G4 concentration of 3 μM in a total of 500 μl volume. Scans were acquired every 5 min for ∼4 h after the addition of 50 μl of amplification grade DNase (1 unit/μL) added immediately after the first scan.

### Analytical ultracentrifugation

Sedimentation velocity measurements were carried out in a Beckman Coulter ProteomeLab XL-A analytical ultracentrifuge (Beckman Coulter Inc., Brea, CA) at 20.0°C and at 50 000 rpm in standard two sector cells. Data (100 scans collected over an 8-hour centrifugation period) were analyzed using the program *SEDFIT* in the continuous c(s) model (www.analyticalultracentrifugation.com). Buffer density was determined on a Mettler/Paar Calculating Density Meter DMA 55A at 20.0°C and buffer viscosity was measured on an Anton Paar Automated Microviscometer AMVn. For the calculation of frictional ratio and molecular weight, 0.55 ml/g was used for partial specific volume (65).

### Molecular modeling

A generic parallel G-quartet stack was built by superimposing the parallel quadruplex structure 1XAV to build a 12-tetrad stacked parallel G-tetrad model with removal of the loops. The appropriate maximum number of G-tetrads, as determined by CD and the sequence, were used to create the central stacked models. Multiple stacked parallel quadruplexes were created, and the loop sequences were manually inserted to minimize the loop length when contiguous guanines in G-runs were greater than the individual number of G-tetrads of a given quadruplex. Potassium ions were added between quartets, and the initial structures were minimized (implicit water solvation, AMBER* force field *Macromodel*, Schrodinger Inc., https://www.schrodinger.com/), whilst restraining the G-tetrads. The models then underwent full AMBER minimization and molecular dynamics using our standard protocol. The models were imported into the xleap module of *AMBER 2018* with the default force field, ff14SB and OL15 DNA force field, neutralized with K^+^ ions, and solvated in a rectangular box of TIP3P water molecules with a 15 Å buffer distance. All simulations were equilibrated using sander using the following steps: (i) minimization of water and ions with restraints of 10.0 kcal/mol/Å on all nucleic acid and amino acid residues (2000 cycles of minimization, 500 steepest decent before switching to conjugate gradient) and 10.0 Å cutoff, (ii) heating from 0 K to 100 K over 20 ps with 50 kcal/mol/Å restraints on all nucleic acid and amino acid residues, (iii) minimization of entire system without restraints (2500 cycles, 1000 steepest decent before switching to conjugate gradient) with 10 Å cutoff, (iv) heating from 100 K to 300 K over 20 ps with restraints of 10.0 kcal/mol/Å on all nucleic acid and amino acid residues and (v) equilibration at 1 atm for 100 ps with restraints of 10.0 kcal/mol/Å on nucleic acids. The output from equilibration was then used as the input file for 100 ns of unrestrained MD simulations using pmemd with GPU acceleration in the isothermal isobaric ensemble (*P* = 1 atm, *T* = 300 K). Periodic boundary conditions and PME were used. 2.0 fs time steps were used with bonds involving hydrogen frozen using SHAKE (ntc = 2). Trajectories were analyzed using the cpptraj module in the *AmberTools 18* package. Accelerated molecular dynamics under the same conditions were also performed for 100 ns trajectories. All systems were stable throughout the production phase.

Hydrodynamic properties were calculated using 500 equally spaced snapshots across the entire trajectory. This was accomplished using *HYDROPRO10* using an atomic level calculation (INMODE = 1, AER = 2.53) with vbar = 0.55. All *HYDROPRO* calculations used temperature of 20.0°C, viscosity = 0.0101 poise and density = 1.0092 g/cm^3^.

### SEC-resolved small-angle X-ray scattering (SEC-SAXS)

All samples analyzed by small-angle X-ray scattering (SAXS) were in BPEK buffer supplemented with 185 mM KCl, purified by preparative SEC (Superdex 75 16/600 SEC column, GE Healthcare 28-9893-33, running at 0.5 ml/min, fractions collected every 2 min), concentrated with Pierce protein concentrators (ThermoFisher, #88515), and dialyzed (Spectra/Por Float-A-Lyzers G2 3.5 kDa, Sigma #Z726060) prior to SAXS analysis. SEC-SAXS was performed at the BioCAT beamline (18ID) at the Advanced Photon Source in Chicago, IL. Prepared samples were centrifuged and subsequently loaded onto an equilibrated Superdex 200 Increase 10/300 GL column (Cytiva) maintained at a flow rate of 0.6 or 0.7 ml/min (see Supplementary Tables S3 and S4) using an AKTA Pure FPLC (GE Healthcare Life Sciences). After passing through the UV monitor, the eluate was directed through the SAXS flow cell, which consists of a 1 mm ID quartz capillary with 20 μm walls. A co-flowing buffer sheath was used to separate the sample and the capillary walls, helping to prevent radiation damage (91). Scattering intensity was recorded with a Pilatus3X1M (Dectris) detector placed 3.628 m from the sample, giving access to a *q*-range of 0.0044–0.35 Å^–1^. A series of 0.5 s exposures were acquired continuously during elution and the data was reduced using the software *BioXTAS RAW versions 1.6.3, 2.0.3 or 2.1.1* (92). Buffer blanks were created by averaging regions flanking the elution peak and subtracted from exposures selected from the sample elution peak to create the buffer corrected I(q) vs. q curves for subsequent analyses. A few of the monomeric G4 sequences (PDB IDs 2KQG, 2LBY, 2M27, 6GH0, 6L92) eluted as oligomeric species and required evolving factor analysis (EFA) (93,94) to retrieve the monomer scattering profile. Singular value decomposition (SVD) and EFA are both standard integrated data deconvolution methods that are integrated in BioXTAS RAW (more information on the use of these methods can be found at https://bioxtas-raw.readthedocs.io/en/latest/). SAXS sample preparation, data collection, data reduction, analysis, presentation, and interpretation have been done in close accordance with recently published guidelines (95). Tabulated results and elution/*R*_g_ profiles from our SAXS analyses can be found in Supplementary Tables S3, S4 and Supplementary Figures S1–S22. All SAXS data have been deposited in the SASBDB (https://www.sasbdb.org/).

Generation of SAXS space-filling envelopes was accomplished using DAMMIF (96) in slow mode with 20 reconstructions (no symmetry or anisometry assumptions) followed by averaging and clustering using DAMAVER (97) and DAMCLUST (98), respectively. The output ‘damstart.pdb’ from DAMAVER was subsequently used as input for a final refinement in DAMMIN (99). The input P(r) distribution files were generated using the program *GNOM 4.6* (100) in RAW v2.1.1 (92) and truncated to the recommended 0.3 q. See Supplementary Tables S3 and S4 for the normalized spatial discrepancy values (NSDs), *χ^2^* values, and resolutions via SASRES (101). The best-fit molecular models were determined using *CRYSOL* v2.8.3 (102) command line interface to perform calculations across 1000 evenly spaced frames from each 100 ns standard MD trajectory. The best fit structure was determined by minimization of a *χ^2^* function:

(1) $$\begin{equation*}{\chi ^2}\left( {{r_o},{\delta _\rho }} \right)\ = \ \frac{1}{{{N_p}}}\mathop \sum \limits_{i\ = \ 1}^{{N_p}} {\left( {\frac{{{I_{exp}}\left( {{q_i}} \right) - cI\left( {{q_i},{r_o},{\delta _\rho }} \right)}}{{\sigma \left( {{q_i}} \right)}}} \right)^2}\end{equation*}$$

where ${I_{exp}}( {{q_i}} )$ and $I( {{q_i}} )$ are the experimental and computed profiles, respectively, *σ(q_i_)* is the experimental error of the measured profile, ${N_p}$ is the number of points in the profile, and *c* is the scaling factor. Two other parameters, ${r_o}$ and ${\delta _\rho }$, are fitted and represent the effective atomic radius and the hydration layer density, respectively. The solvent electron density used in *CRYSOL* calculations was adjusted for the buffer components from 0.334 to 0.3368 e^–^/Å^–3^. Best fit atomistic models were docked to their most probable space-filling envelopes using SUBCOMB (103) and visualized in Chimera v1.12.

Sedimentation coefficient calculations from SAXS *ab initio DAMMIF/N* refined bead models were done in the following way. First, theoretical volumes were calculated for each system with the following equations:

(2) $$\begin{eqnarray*}{V_{anhydrous}}\ \left({{{\AA}^3}} \right) &=& \ M\left( {Da} \right)\nonumber\\ && \times \frac{{\left( {0.55\ \left( {\frac{{c{m^3}}}{g}} \right)} \right) \times \left( {{{10}^{24}}\left( {\frac{{{{\AA}^3}}}{{c{m^3}}}} \right)} \right)}}{{6.023x{{10}^{23}}\left( {\frac{{Da}}{g}} \right)}}, \end{eqnarray*}$$

(3) $$\begin{equation*}\left( {\left( {\frac{\delta }{{\rho \times \upsilon }}} \right) + 1} \right) \times {V_{anhydrous}}\end{equation*}$$

where $\rho$ is the solution density and the partial specific volume, $\upsilon = 0.55$ cm^3^/g was assumed. Volumes were then adjusted to reflect an assumed hydration of $\delta = 0.3$ g/g H_2_O (64) (Equation 3). For lower-resolution SAXS models, HYDROPRO10 (104) recommends that calculations be performed with INDMODE = 1 and an AER (hydrodynamic radius of elements in primary model) value that results in agreement between calculated and expected particle volume. The adjusted AER should also yield *R_g_* in good agreement with that measured by SAXS. Starting values for AER were derived from the *DAMMIF/N* refined model header under ‘average volume per atom’, *V_a_*, and the following equation for radius of a sphere:

(4) $$\begin{equation*}AER\ = \ \sqrt[3]{{\frac{{3 \times {V_a}}}{{4 \times \pi }}}}\end{equation*}$$

The resulting AER values were then fed into HYDROPRO and, if necessary, adjusted until the calculated volumes were within 5% of expected values. In each case, the resulting *R_g_* values were within 4% of their measured value. Results are tabulated in Supplementary Table S5.

## RESULTS

### Bioinformatic queries show that long sequences might form higher-order G4 structures and are abundant in promoter regions of the human genome

We used *Quadparser(2)* to search human promoter sequences between −750 or −499 to + 100 relative to the transcriptional start sites for various combinations of two or three contiguous guanines in runs of 4, 8, and 12 tracts interspersed by loop lengths ranging from 1–7 to 1–10 (Supplementary Table S2). We searched for the motifs [G_*x*_-L_*y*_-G_*x*_-L_*y*_-G_*x*_-L_*y*_-G_*x*_]_*z*_, where *x* = 2, 3, *y* = 1–7, 1–8, 1–9, 1–10, *z* = 1, 2, 3 and where an additional loop, L_*y*_ is included between each motif block when *z* = 2 and 3. Such sequences might form multiple G4 structures. We found hundreds of thousands of potential G4 forming sequences identified with over 56 000 promoter sequences with eight tracts of G_2,3_ and over 20 000 with 12 tracts of G_2,3_ with loops of 1–10 bases. From the abundance of these promoter sequences, we chose 8- and 12-tract sequences from the oncogene promoters of *c-Myc* (‘c-Myc-8’, ‘c-Myc-12’), *c-Kit* (‘c-Kit-8’,’c-Kit-12’), and *k-Ras* (‘k-Ras-8’) to characterize on the basis of their disease relevance (Table 2). What follows is our application of our ISB approach to characterize the structure of these sequences.

### The CD 264 nm peak amplitude correlates with G-quartet number in stacked parallel intramolecular quadruplexes

**Figure 2.**
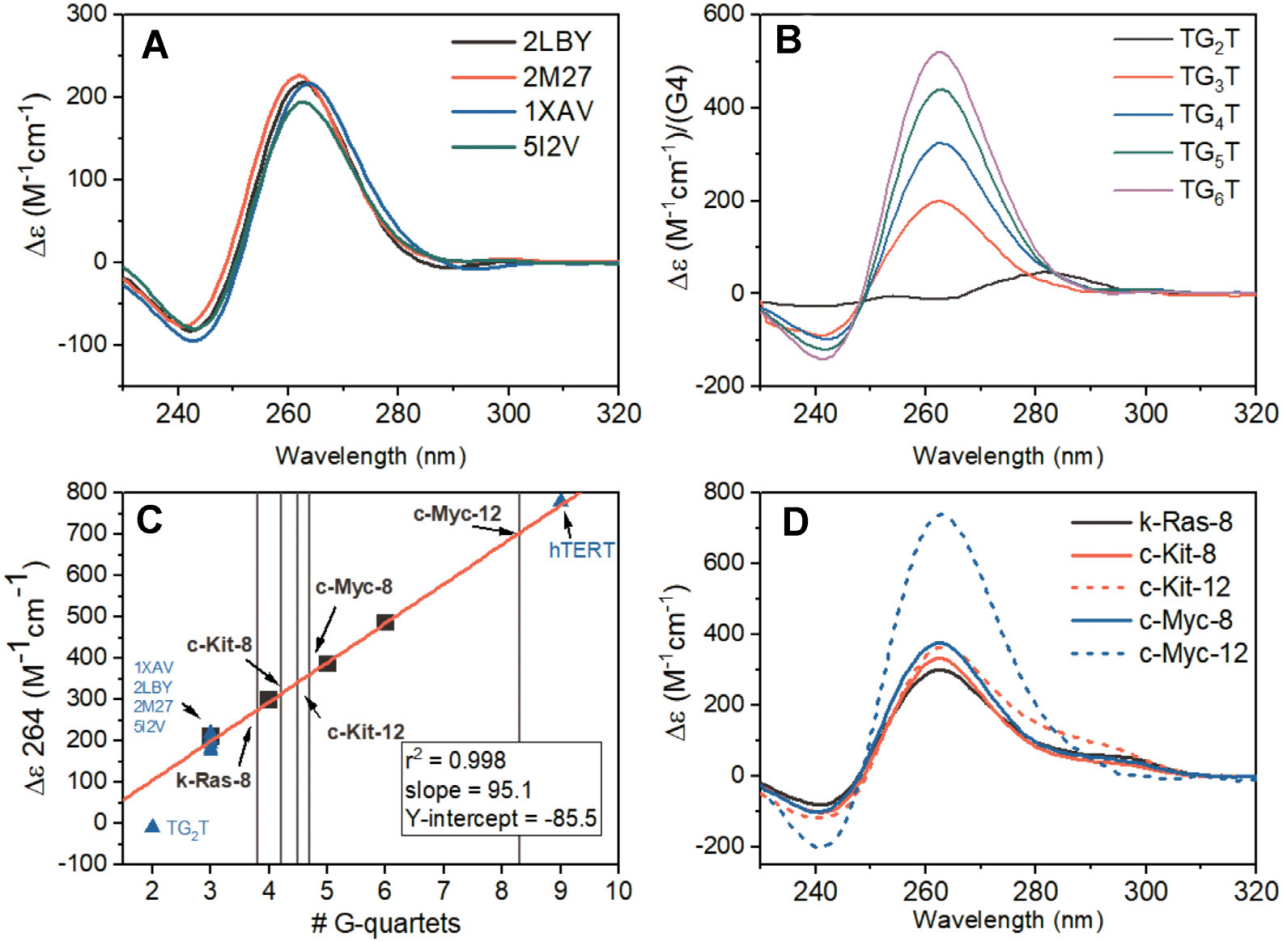
(**A**) CD spectra of parallel promoter G4s with three G-quartet stacks from the literature. (**B**) CD spectra of d[TG_*n*_T]_4_ oligos normalized to G4 concentration. (**C**) Linear relationship of the 264 nm CD signal normalized by G4 concentration of parallel d[TG_*n*_T]_4_ G4s (black squares) versus number of contiguous stacked G-quartets. The line of best fit is shown in red. The blue triangles show the 264 nm magnitudes for the promoter sequences in (A), the magnitude of the 9-quartet hTERT (52), and the TG_2_T sequence (which does not form a G4). The light gray vertical lines cross the regression at the 264 nm values obtained for the extended promoter 8-tract and 12-tract sequences (shown in D). (**D**) CD spectra of the 8-tract and 12-tract promoter sequences studied here.

We observed that parallel three-tetrad G4s exhibit similar spectral shapes and CD 264 nm amplitudes (∼200 Δϵ) (Figure 2A). The amplitude of a single G4 is approximately 1/3 to 1/4 of the nine-tetrad stack parallel hTERT promoter quadruplex (∼750 Δϵ) (52). To determine if the magnitude of the 264 nm CD signal is correlated with the number of stacked G-tetrads, we measured the CD spectra of a series of well-characterized, all-parallel tetrameric G4s with different numbers of stacked quartets that form with the sequences d[TG_*n*_T] (*n* = 3–6) (Figure 2B). To ensure formation of d[TG_*n*_T]_4_ rather than misfolded structures, it was necessary to initiate G4 formation by adding K^+^ to a concentrated (∼1 mM) solution of monomeric oligonucleotide (90). This procedure ensures rapid, in-register formation of tetrameric G4s rather than G-wires or other misfolded structures. The homogeneity of these tetramolecular structures was confirmed by molecular weights and sedimentation distributions by analytical ultracentrifugation. There is a clear linear relationship between the 264 nm ellipticity normalized with respect to the number of contiguous G-tetrad stacks per tetramer (Figure 2C). In addition, the normalized CD amplitude determined for the 3-tetrad, parallel promoter G4s of known structure all fall on the calibration line. These include: *k-Ras*, 5I2V (46); *c-Myc*, 2LBY (71); *c-Myc*, 1XAV (44); *VEGF*, 2M27 (73) and *hTERT* (52). Linear least-squares fitting of the d[TG_*n*_T]_4_ data provided a slope *m* = 95.1 ± 3.8 M^–1^ cm^–1^/quartet and an intercept *b* = −85.5 ± 16.4 M^–1^ cm^–1^, with a correlation coefficient *r*^2^ = 0.998.

Figure 2D shows the normalized CD spectra of the five putative higher-order promoter G4 sequences, c-Myc-8, c-Myc-12, c-Kit-8, c-Kit-12 and k-Ras-8, identified in our bioinformatic inquiry. In each case, the CD spectrum exhibits a maximum near 264 nm and minimum at 240 nm, consistent with parallel G-quadruplex formation. The number of G-tetrads per promoter strand estimated from the slope of the calibration curve in Figure 2C is summarized in Table 2. The presence of a non-integral number of stacks could reflect some slight structural heterogeneity. The 3-tetrad, parallel promoter G4s in Figure 2A show a 264 nm deviation about the linear regression fit of up to 7%. Each of the extended promoter sequences, aside from c-Kit-12, are within 7% of their integral G-tetrad number, i.e. c-Kit-8 and k-Ras-8 have ∼4 G-tetrads, c-Myc-8 has ∼5 G-tetrads, and c-Myc-12 has ∼8 G-tetrads. The 295 nm shoulder in the c-Kit-12 spectrum raises the possibility that its 264 nm value is influenced by the presence of other structures, either an antiparallel quadruplex, a hairpin, or a combination thereof (as shown below). By enumerating the number of stacked quartets in the structures formed by each promoter sequence, these CD results provide quantitative constraints for model building, an important first step in the integrated structural strategy.

### Analytical ultracentrifugation sedimentation velocity (AUC-SV) studies assess homogeneity, compactness and hydrodynamic shape of G-quadruplexes

**Figure 3.**
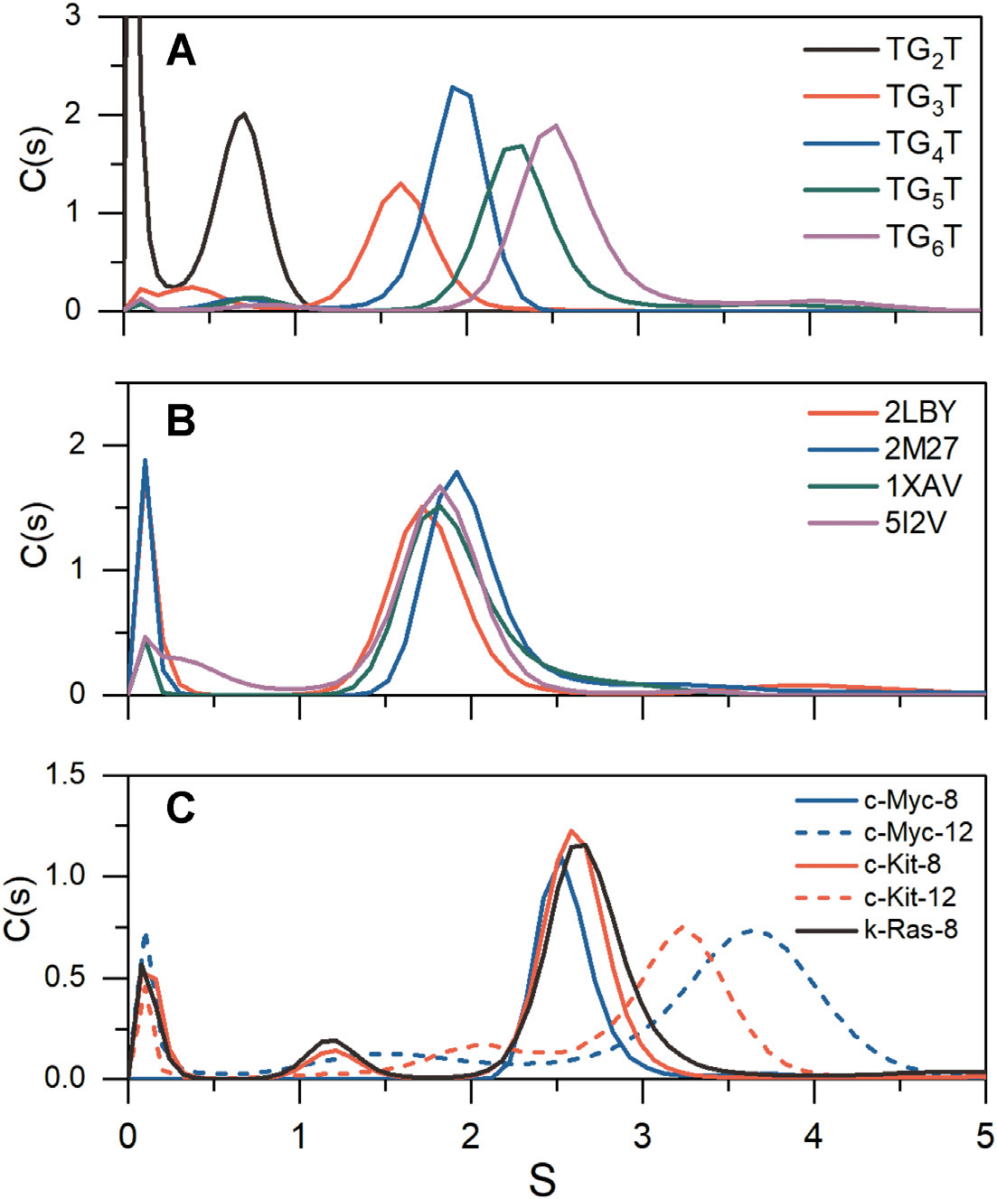
Sedimentation velocity profiles for G4s. (**A**) shows d[TG_*n*_T]_4_ oligos. (**B**) shows the promoter G4s from Figure 1A, and (**C**) shows the c-Myc, c-Kit and k-Ras higher-order promoters. Tabulated frictional ratios and sedimentation values corrected to reflect water at 20°C (*S*_20,w_) are given in Table 3.

After sequence identification and characterization by CD, the next step in our ISB workflow is to assess folding of each sequence into a discrete structure and the homogeneity of samples (Figure 1 and Table 1). AUC-SV is an ideal tool for this purpose, since it can provide unambiguous evidence of heterogeneity, estimates of the molecular weights of all species present, and low-resolution shape information (105). As demonstrated in Figure 3A, the tetrameric G4s (d[TG_*n*_T]_4_) are highly stable, even on 50-fold dilution to working concentrations suitable for spectropolarimetry. The C(s) distribution analysis shows >95% tetramolecular species for each of the d[TG_*n*_T]_4_ series used for calibration, confirming that the CD signals in Figure 2 arise from the expected tetrameric species and are not influenced by aggregates or unfolded single strands. Similarly, the 3-stack promoter parallel G-quadruplexes *k-Ras* 5I2V (46), *c-Myc* 2LBY (71), *c-Myc* 1XAV (44) and *VEGF* 2M27 (73) (Figure 3B) are all folded and homogeneous. Figure 3C, shows the *C*(*s*) distributions of the extended promoter sequences. All AUC-derived molecular weights are within 10% of their true molecular weights. Qualitatively, the distributions show that the 8-tract promoter sequences exhibit sedimentation coefficients close to those of the 5- and 6-tetrad d[TG_*n*_T]_4_ sequences (which are of similar MW), and much higher than the 3-stack promoter G4s, consistent with compact unimolecular folded species. We note that in Figure 2D, the c-Kit-12 CD spectrum is similar in 264 nm magnitude to the 8-tract promoter G4s, indicating similar number of tetrad stacks, yet it exhibits a sedimentation coefficient that is in between the 8-tract promoters and c-Myc-12. The measured frictional ratios (*f/f_o_*) for all sequences are <1.5 confirming that all sequences contain secondary structure and are not random coils (Table 3) (80). The AUC-SV results provide additional quantitative constraints for model building.

**Table 3.** Summary of analytical ultracentrifugation and HYDROPRO results. For the higher-order 8- and 12-tract promoters (c-Kit, k-Ras, and c-Myc) the HYDROPRO calculated results are reflective of only the final best fit all-parallel model builds. SAXS *ab initio* HYDROPRO calculations were done using the final refined models from *DAMMIN* and an AER that was adjusted until the HYDROPRO calculated volumes and radii of gyration were within ∼5% of the expected values (from calculation or SAXS measurements, respectively) (see Supplementary Table S5)

Sequence	G4 molecularity	AUC MW (kDa)	True MW (kDa)	*f/f_o_*	*S*_20,w_ observed	*S*_20,w_ calculated (all atom)	*S*_20,w_ calculated (SAXS *ab initio*)
TG3T	(tetramer)	6.6	6.14	1.32	1.75		
TG4T	(tetramer)	7.7	7.45	1.25	2.05		
TG5T	(tetramer)	10.2	8.77	1.27	2.44		
TG6T	(tetramer)	11.3	10.09	1.24	2.67		
1XAV	(monomer)	8.3	6.99	1.38	1.86		
2LBY	(monomer)	7.1	6.05	1.38	1.72		
2M27	(monomer)	7.9	6.91	1.24	2.10		
5I2V	(monomer)	7.9	6.97	1.49	1.76		
c-Myc-8	(monomer)	12.0	10.98	1.33	2.60	2.16–2.52	2.53
c-Myc-12	(monomer)	22.3	22.25	1.46	3.57	3.68–3.78	3.04
c-Myc-12 (Post DNaseI digestion)	(monomer)	20.6	-	1.41	3.61	-	3.26
c-Kit-8	(monomer)	12.9	12.14	1.34	2.70	2.08–2.61	2.46
c-Kit-12	(monomer)	19.2	20.34	1.42	3.33	3.38–3.73	2.96
c-Kit-12 (Post DNaseI digestion)	(monomer)	14.7	-	1.42	2.85	-	2.82
k-Ras-8	(monomer)	14.6	13.75	1.4	2.83	2.79–2.85	2.56

### Small-angle X-ray scattering measures the global shape of G4 structures

SAXS is a powerful technique for characterizing complex higher-order G-quadruplex systems (52,81). SAXS provides both qualitative and quantitative structural information, including particle shape, compactness, volume, maximum particle dimension (*D_max_*) and radius of gyration (*R_g_*) (106,107). Scattering data are especially powerful when combined with molecular modeling (95,108), as scattering patterns may be readily computed from *in silico* models with programs such as *CRYSOL* (102). In this way, models can be refined directly against ‘medium’ (∼18–30 Å) resolution experimental structural information (107–109).

**Figure 4.**
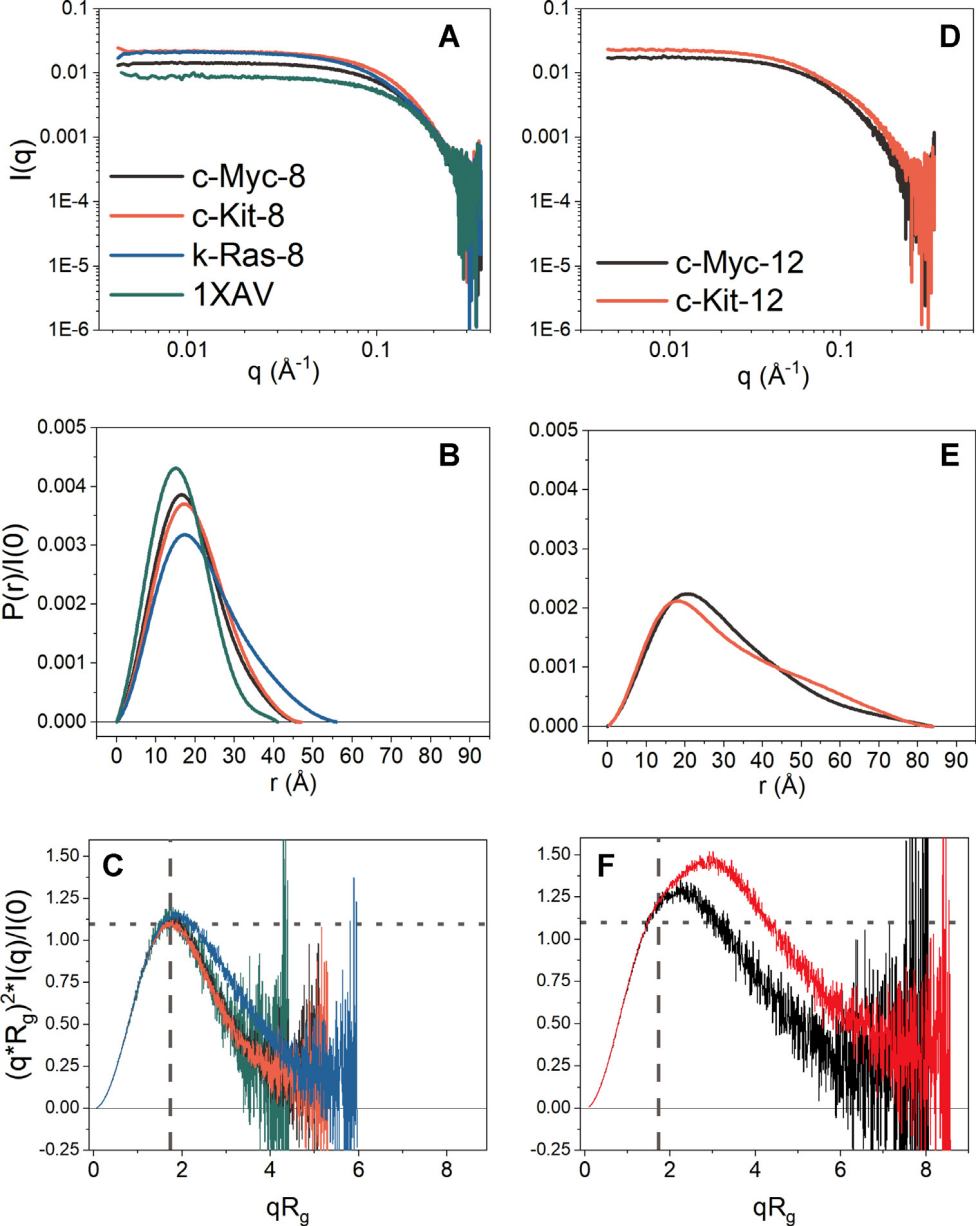
SEC-SAXS analysis of 8- and 12-tract promoter G-quadruplex sequences. (**A**, **D**) Log-linear plots of buffer subtracted scattering of 8-run (A), and 12-run (D) sequences. (**B**, **E**) Pair distance distribution functions, P(r), normalized to scattering intensity at zero angle (*I*(0)) for the 8-run (B) and 12-run (E) sequences. Both plots have the same X-axis to emphasize differences in *D*_max_, the maximum interparticle distances. (**C**, **F**) Dimensionless Kratky plots of 8-run (C) and 12-run (F) sequences with grey dashed and dotted lines overlaid to illustrate where on the X- and Y-axis a peak is expected for a globular particle. Included in panels A-C are the SAXS results of the 4-tract parallel c-Myc G-quadruplex 1XAV (in green) to contrast with the higher-order promoter sequences. Tabulated results are given in Supplementary Tables S3 and S4.

The most important consideration prior to the interpretation of SAXS data is that there is no aggregation, radiation damage, or interparticle interactions (95). To ensure this, SAXS measurements were made as a function of elution time from an SEC column with a co-flowing buffer sheath to mitigate X-ray damage. The final scattering profiles of the 8- and 12- tract promoter sequences were monodisperse based on linearity of Guinier regression analysis (Supplementary Figures S1–S7), in agreement with AUC analysis (although we note minor amounts of larger and extended species are evident in c-Myc-12 and c-Kit-12 scattering and AUC profiles, respectively) (95). The 8- and 12-tract sequence SAXS results are shown in Figure 4 and a description of the data collection, reduction, and analysis are given in Supplementary Table S3. We included the analysis of 1XAV to serve as a ‘control’ for a globular particle and to aid in comparison of some key features of the results (2LBY, 2M27 and 5I2V were also analyzed but not shown; their scattering results are in Supplementary Table S4 and Supplementary Figures S8, S14, S15, S17). Figure 4A and D shows that each sequence has scattering that is horizontal and parallel to the X-axis at low *q*, supporting that the data are free from artifacts due to inter-particle interactions. The data are presented on a log–log scale to highlight the smooth curvature at ∼0.1 *q*. This smooth curvature is characteristic of globular particles (107), and all but c-Kit-12 exhibit a rounded decay at higher values of *q*.

The pair distance distribution functions, or P(r), plots for the extended promoter sequences, c-Kit-8, c-Myc-8, k-Ras-8, c-Myc-12 and c-Kit-12 are shown in Figure 4B and E. The *P*(*r*) plot is an *r*^2^-weighted real-space histogram of inter-atomic distances derived from the scattering by an indirect Fourier transform (107). P(r) distributions that are symmetric and Gaussian are indicative of globular shapes, as exemplified by 1XAV (Figure 4B, green) (106,107). Deviations from Gaussian shape, such as skewing or multiphasic distributions, indicates deviation from a globular particle, e.g. asymmetric oblate or prolate particles, multi-domains, or regions of disorder (107). Quantitative information is also gained from the *P*(*r*) plot. The maximum dimension of the particle, or *D_max_*, is where the curve intercepts the X-axis. The radius of gyration, *R_g_*, is an overall size estimate useful for comparisons with calculations from theoretical atomistic models. The *R_g_* can be determined from the second moment of the *P*(*r*) distribution (which is sometimes more reliable than the Guinier approximation) (106,107). *D_max_* and *R_g_* values for each sequence are tabulated in Supplementary Table S3. Figure 4B shows the P(r) distributions, normalized to *I*(0), for 1XAV, c-Myc-8, c-Kit-8 and k-Ras-8. In each case, the 8-tract promoter sequences are globular and of larger dimension than 1XAV, based on their slight positive skew. There is good agreement between Guinier and *P*(*r*)-derived I(0) and *R_g_* values for the 8-tract sequences, consistent with a globular and folded particle (Supplementary Table S3). In contrast, the 12-tract promoter sequences (Figure 4E) exhibit significant positive skew with a gradual decline to *D_max_*. The difference of 2–5% in the measured Guinier and *P*(*r*)-derived *I*(0) and *R_g_* values (Supplementary Table S3) indicates some amount flexibility (106). The latter may be attributed to large loop regions, co-existing G-register isomers, or general unstructured regions (61). In the case of c-Kit-12, a distinct shoulder is evident in the r-range of ∼40–65 Å which is characteristic of a multi-domain particle (107).

Another qualitative assessment of particle compactness and flexibility is a mathematical transformation of the scattering data into a Kratky plot (Figure 4C and F) (106). Scattering from a spherical particle decays rapidly at large angles, *I*(*q*) ∼ 1/*q*^4^, and so by plotting the scattering as *q*^2^*I(*q*) versus *q* a Gaussian profile is expected for globular species (106). To make interpretation more semi-quantitative, the Kratky plot can be made ‘dimensionless’ by multiplying *q* by *R_g_*, to account for particle size, and scaling the intensity by *I*(0), to account for mass. For a globular particle, this transformation yields a peak at X- and Y-dimensions of *qR_g_* = √3 = ∼1.75, and 3/*e* = 1.104, respectively (110). Fully unfolded particles will not return to zero at high *qR_g_*, but instead continue rising to a plateau at constant value of 2 (110). Structured particles with flexibility are expected to exhibit intermediate curves between these two extremes, e.g. a peak position shifted positively in both X- and Y-directions. Figure 4C and F show the dimensionless Kratky plots for each of the promoter sequences and 1XAV. 1XAV exhibits a Kratky profile with classical symmetric Gaussian shape about ∼1.73 *qR_g_*, with peak height of ∼1.1, consistent with its globular shape. Similarly, the 8-tract sequences exhibit curves that are bell-shaped, albeit slightly skewed relative to 1XAV (Figure 4C), indicating slight deviation from an entirely globular shape. In contrast, c-Myc-12 exhibits a highly skewed profile that may signify a globular shape with protruding flexible domain (Figure 4F). c-Kit-12 also exhibits a skewed globular profile; however, a notable monotonic increase is evident from ∼1.8–3.5 *qR_g_*, signifying that it is multi-domain and flexible (Figure 4F). Altogether, these results are consistent with the AUC analyses in that all 8-tract sequences adopt folded, compact tertiary structures and that the 12-tract sequences are overall globular but more complex than a simple oblate or prolate particle in their tertiary structure.

### Validation of CRYSOL *R*_g_ calculations as an ISB tool for filtering models of inconsistent size

**Figure 5.**
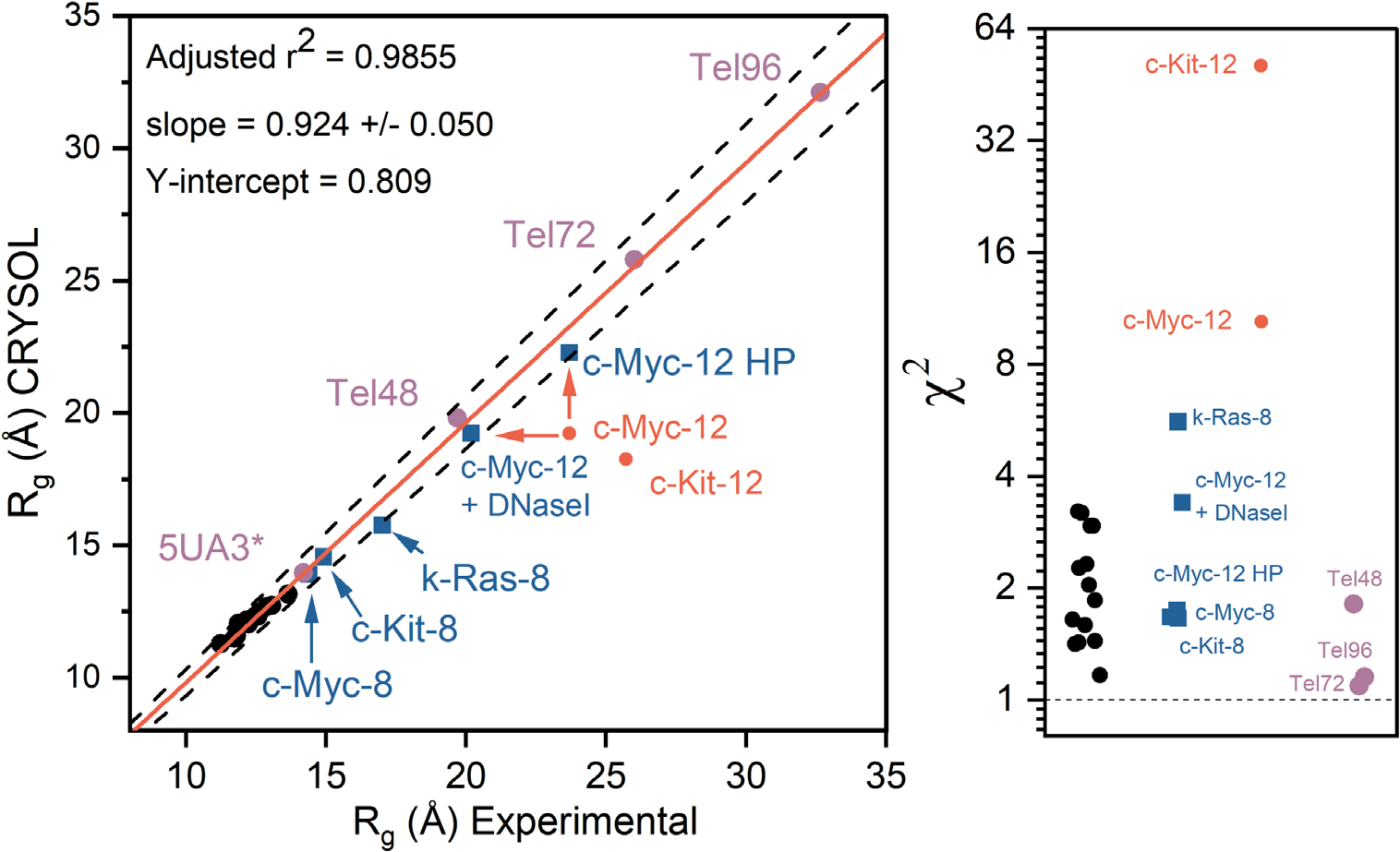
Correlation of experimental and *CRYSOL* calculated radii of gyration for known G4 structures (left) and corresponding χ^2^ values for each model (right). Black circles represent G4 structures that have been previously solved by either NMR (13/14) or X-ray (1/14) crystallography (tabulated values given in Supplementary Table S4 and *CRYSOL* fits in Fig. S22). Blue squares represent the G4 models derived from this study that are consistent with the collective experimental data. Red circles indicate parallel stacked models from this study that deviate from one or more biophysical measurements (initially). The red arrows indicate changes in correlation of c-Myc-12 either with DNaseI treatment or by modeling in competing hairpins. Purple circles are values obtained from prior G4 SAXS studies (81,112). In the case of the telomere sequences (Tel48, 72 and 96) the regression values were obtained from EOM analyses (118). The dashed black lines indicate a ± 5% of the line of best fit.

To date, no studies have systematically evaluated how well the *R_g_* values of atomistic models of G4s compare with their measured values. In a similar manner to the above CD analysis and our prior hydrodynamic bead modeling studies (65), we sought to determine the degree to which *R_g_* values calculated from atomic models agree with their SAXS-measured radii of gyration. To examine this, we collected SAXS data on 14 sequences from monomeric G4s deposited in the Protein Data Bank that had been studied in K^+^-containing buffers, including telomere, promoter, and artificial sequences (13 from NMR, 1 from XRD). The scattering, Guinier, Kratky, and P(r) distributions are shown in Supplementary Figures S8–S21. For *R_g_* determination, each PDB structure was stripped of ions, waters, and co-solutes, and minimized with K^+^ between G-tetrads prior to *CRYSOL* calculation (102). In *CRYSOL*, obtaining an *R_g_* from a model first requires calculation of the theoretical scattering, $I( q )$, after which the theoretical *R_g_* can be derived from the slope of the net intensity. If the experimental scattering, ${I_{exp}}( q )$, is supplied, *CRYSOL* will fit the theoretical scattering from model to the experimental by adjusting two free parameters that account for the average displaced volume per atomic group (or the effective atomic radius), ${r_o}$, and the contrast of the hydration layer, ${\delta _\rho }$. Without adjusting for hydration, systematic errors can arise leading to large discrepancies between resulting *R_g_* values. The theoretical scattering is then calculated as a function of both parameters, $I( {q,{r_o},\ {\delta _\rho }} )$, with initial values of ${r_o}$= 0.162 nm and ${\delta _\rho }$ = 30 e^–^/nm^3^. Next, a grid search is performed to identify optimal values of ${r_o}$and ${\delta _\rho }$that minimize the error-weighted χ^2^ discrepancy function in Equation (1). The grid search spans the ranges ${r_o},$ = 0.156 – 0.168 nm and ${\delta _\rho }$= 0–70 e^–^/nm^3,^ as these values encompass what has been experimentally observed (102). The resulting *R_g_* and χ^2^ values are plotted in Figure 5 and tabulated in Supplementary Tables S3, S4 (see Supplementary Figure S22 for fits). There is a very high correlation between experimental and calculated radii of gyration (*r*^2^ = 0.9855), with slope only slightly under 1. Importantly, the χ^2^ values of the known structures ranged from 1.17–3.22 with average of 2.1 ± 0.7, indicating that each model fit their respective scattering profile well. This range of χ^2^ values is comparable to *CRYSOL* benchmarking studies of proteins (although with differences in the number of data points used in the calculations) (111). Included in this regression are the higher-order telomere models derived previously (81) and PDB ID 5AU3 (112), the only other available G4s with SAXS data available, for a total of 18 data points. For the 14 PDB G4 structures, the average and standard deviation of ${r_o}$ and ${\delta _\rho }$ are 1.64 ± 0.19 Å and 60 ± 2 e^–^/nm^3^, respectively. The former value is consistent with the effective atomic radii reported for proteins and RNA (102,113) and the latter value is within the range observed for protein and RNA systems (with higher values associated with highly charged molecules) (102,113,114). We note here that the hydration density parameter could also be influenced by counterion condensation (114,115); however, this is outside the scope of our present study. The excellent agreement between model and measured *R_g_* indicates that the radius of gyration is useful as another metric to filter ill-fitting models. However, as we will show, a more rigorous criterion for model *inclusion* is the combination of *R_g_* agreement and low χ^2^ value.

### SAXS-MD model construction and refinement reveal that the 8-tract extended promoters form 4- or 5-quartet stacked parallel G-quadruplexes

The 8-tract structural models were constructed in the following way. First, the number of contiguous G-tetrad stacks formed by each sequence was constrained by values from CD analysis shown in Table 2 (c-Kit-8 and k-Ras-8 with 4 tetrads, c-Myc-8 with 5 tetrads). Next, G-tetrad columns were constructed using the known core G-tetrad stack structure of 1XAV. Each loop was then built into place using known G-quadruplex loop backbone orientations where possible. Adjustments to loop placement and geometry were made iteratively by checking the calculated sedimentation coefficient for the model against those measured by AUC. Models were then optimized and equilibrated as described in the methods section. Each system was then subjected to unrestrained, explicit solvent MD simulations for at least 100 ns. The calculated and experimental hydrodynamic parameters are given in Table 3 for each model. The calculated sedimentation coefficient ranges of the models constructed encompass the experimental values or are at most 0.1 S outside of the range.

**Figure 6.**
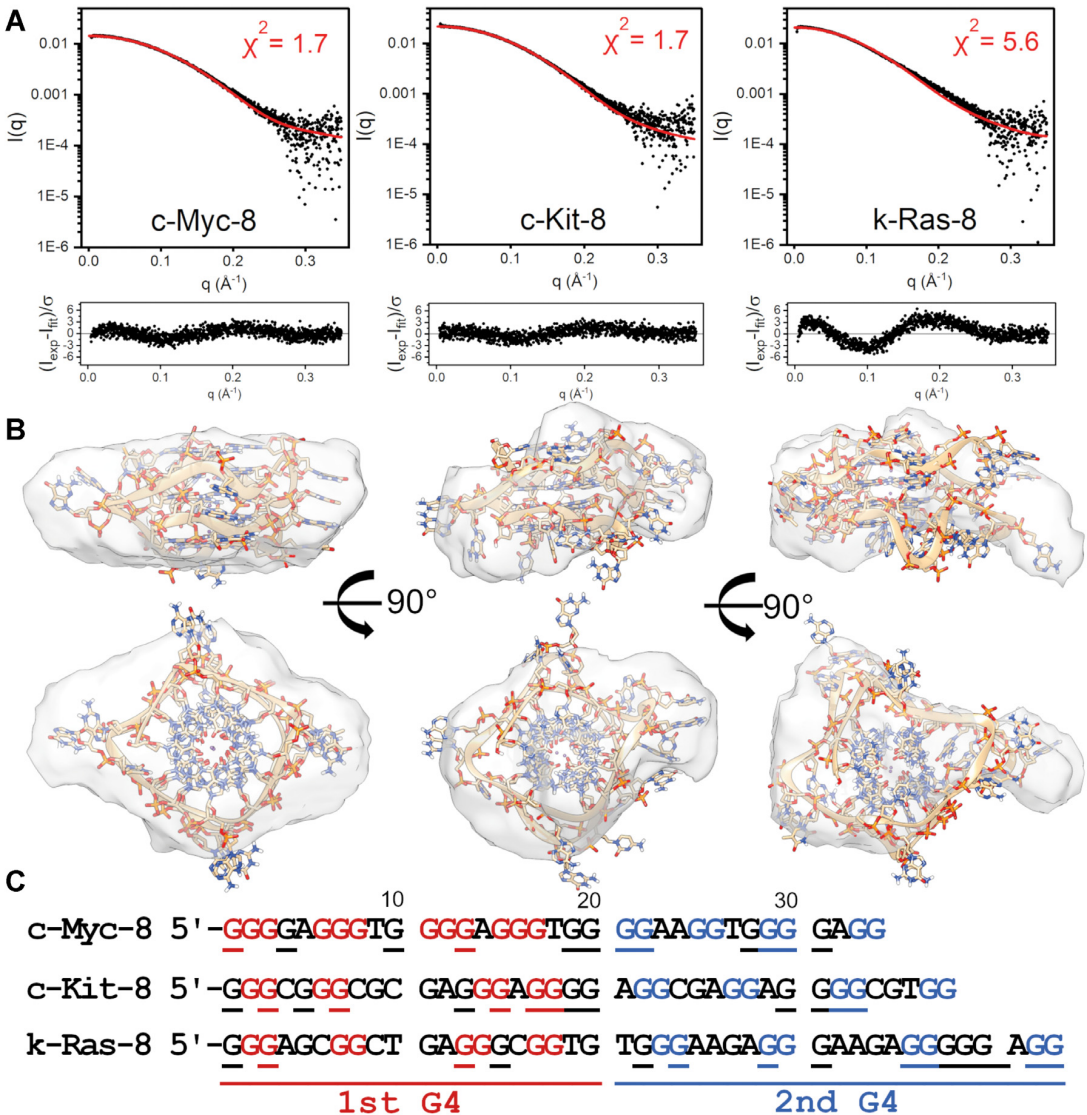
SAXS scattering profiles and CRYSOL fits for molecular models of c-Myc-8, c-Kit-8, and K-Ras-8 promoter sequences. (**A**) For each sequence the scattering is represented on a Log-linear scale in black, with the calculated profile fit from CRYSOL shown in red. Inset is each model's χ^2^ value. See methods for χ^2^ equation and parameter fitting. (**B**) Below each figure are the best fitting atomic models superimposed in their respective *DAMMIF/N* refined envelopes. (**C**) Sequences with red and blue coloration to highlight the guanines incorporated in each G4 of each model shown in (B), with underlined residues being those with the potential to be utilized by G-register isomers.

We next used *CRYSOL* to compute the χ^2^ fit values of 1000 equally spaced PDB frames from across each refined model's trajectory using their experimental scattering curve from Figure 4A. The *R_g_* and χ^2^ values of the best-fit models are shown in Figure 5. As shown in Figure 6, the atomistic models fit well into their *DAMMIF/N-refined* SAXS envelopes. Ambiguity assessments by *AMBIMETER* (116) gave ambiguity scores of 1.00, 1.08 and 1.53 for c-Myc-8, c-Kit-8 and k-Ras-8, reconstructions, respectively, indicating that the envelopes have little shape ambiguity (Supplementary Table S3). Further, each reconstruction agreed well with their measured *R_g_* and *D_max_*, had good agreement with known MWs, and normalized spatial discrepancy values (NSDs) of less than 1.0 (Supplementary Table S3). As a check of reconstruction validity, each *ab initio* model was used as input for HYDROPRO calculations to see if the shape is consistent with hydrodynamic measurements. Table 3 shows that c-Myc-8’s *ab initio* model was within 4% of its measured *S*_20,w_, whereas c-Kit-8 and k-Ras-8 reconstructions are 9% off from their measured value (although c-Kit-8 is well within the *S*_20,w_ range estimated from MD simulations of the all-atom model).

The atomistic models fit their scattering with χ^2^ values of 1.7, 1.7 and 5.6 for cMyc-8, cKit-8 and kRas-8, respectively, as determined by *CRYSOL*. The χ^2^ fit value can be influenced by the noise in the scattering data (see Equation 1). However, since we have data on 14 structural models of G4s under identical buffer conditions, and the scattering in each case has reasonable S/N, we could compare our χ^2^ fit values to those obtained for known structures. The average and standard deviation of our G4 library χ^2^s is 2.1 ± 0.7, indicating that c-Myc-8 and c-Kit-8 models fit their scattering data well relative to known atomic G4 models (see Figure 5). k-Ras-8 has an *R_g_* that is in good agreement with its measured value, but the model had a poor fit to its scattering, possibly reflecting coexistence of conformational isomers, which will be discussed below. This emphasizes *R_g_* agreement alone is not sufficient for model validation. The various models built, simulated, and filtered out for the c-Myc-8 and c-Kit-8 G4s had χ^2^ values on the order of 1.8–2.0 (G-register and loop isomers), 2.4–10.0 (fewer G-tetrad stacks) and >>10.0 (all others), supporting that SAXS can discriminate G-register or loop isomers, and can easily filter out models with inconsistent topologies.

### Inclusion of secondary structure prediction to refine models from SAXS data: Fold prediction and DNaseI cleavage analyses reveal that the 12-tract promoter sequences have competition between parallel G-quadruplex and hairpin features

Using the same modeling approach as outlined for the 8-tract sequences, *in silico* all-parallel stacked models were generated for c-Myc-12 and c-Kit-12. Although both models were in near agreement with their sedimentation values (Table 3), both deviated significantly from their experimental radii of gyration (red points in Figure 5). Based on the *P*(*r*) and Kratky analyses in Figure 4, we reasoned that unanticipated competing secondary structures, such as large loop regions, unstructured single-strands, or duplexes (69), could be responsible for the much larger than anticipated *R_g_*s. To test this possibility, we submitted each sequence, along with the 8-tract sequences, to the Mfold (117) server with 0.2 M monovalent cation concentration and the rest of settings at default values (Supplemental Figures S23–S27). For the three 8-tract sequences, there is minimal likelihood of competing thermodynamically stable duplex features (ΔGs from +3.7 to –1.9 kcal/mol). In contrast, c-Myc-12 and c-Kit-12 are predicted to have stable competing duplex features (Δ*G*s from −5.1 and −8.1 kcal/mol, respectively) that might need to be included in structural models.

**Figure 7.**
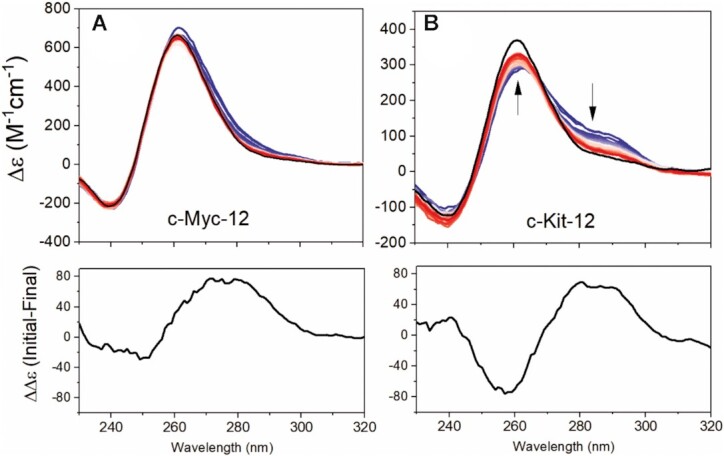
DNaseI digestions monitored by CD over 3 hours (blue to red) and overnight (black line) of c-Myc-12 (**A**) and c-Kit-12 (**B**) sequences. Shown below each digestion profile is the difference spectrum created by subtracting the final (overnight black line) from the initial spectra.

DNaseI, which does not cleave parallel G4s, can be used as a probe to test for duplex or hairpin features (52) within c-Myc-12 and c-Kit-12. The results of CD monitored DNaseI cleavage assays are shown in Figure 7. The spectral changes observed for the c-Myc-12 digestion were subtle (Figure 7A), but the CD difference spectrum is qualitatively consistent with digestion of non-G4 elements, as evident by the spectral magnitude, maximum at ∼280 nm and minimum at 250 nm. The c-Kit-12 digestion showed more pronounced changes in CD (Figure 7B) with a substantial 264 nm increase and reduction at 285 nm, consistent with a conversion to an all-parallel G4. The resulting CD difference spectrum, with ∼280 nm maximum and ∼255 nm minimum, is consistent with B-form hairpin DNA in both shape and magnitude (84).

From the Mfold analyses, both c-Kit-12 and c-Myc-12 were predicted to have hairpin loops that could compete with quadruplex formation. To investigate the extent to which duplex features contributed to each sequence, we analyzed their post-digest products by AUC-SV (Table 3). Surprisingly, c-Myc-12 had only a subtle reduction in molecular weight (∼2 kDa), corresponding to just a few nucleotides, yet had an increase in *S_20,w_* with decreased *f/f*_o_. Combined with the CD digestion analysis, this indicates that the remaining particle is a compact parallel G-quadruplex. The presence of only minor amounts of hairpin formation is consistent with c-Myc-12’s proton NMR spectra (Supplemental Figure S28) which shows an 18:1 ratio of G4 imino protons to Watson-Crick imino protons. Conversely, c-Kit-12 had a digestion product that is hydrodynamically and spectroscopically equivalent to the c-Kit-8 G4 (compare in Table 3 and Figure 2). Since c-Kit-12 fully encompasses the c-Kit-8 sequence, this suggests that the remaining particle from DNaseI cleavage is the c-Kit-8 parallel four stack quadruplex described above (Figure 6).

**Figure 8.**
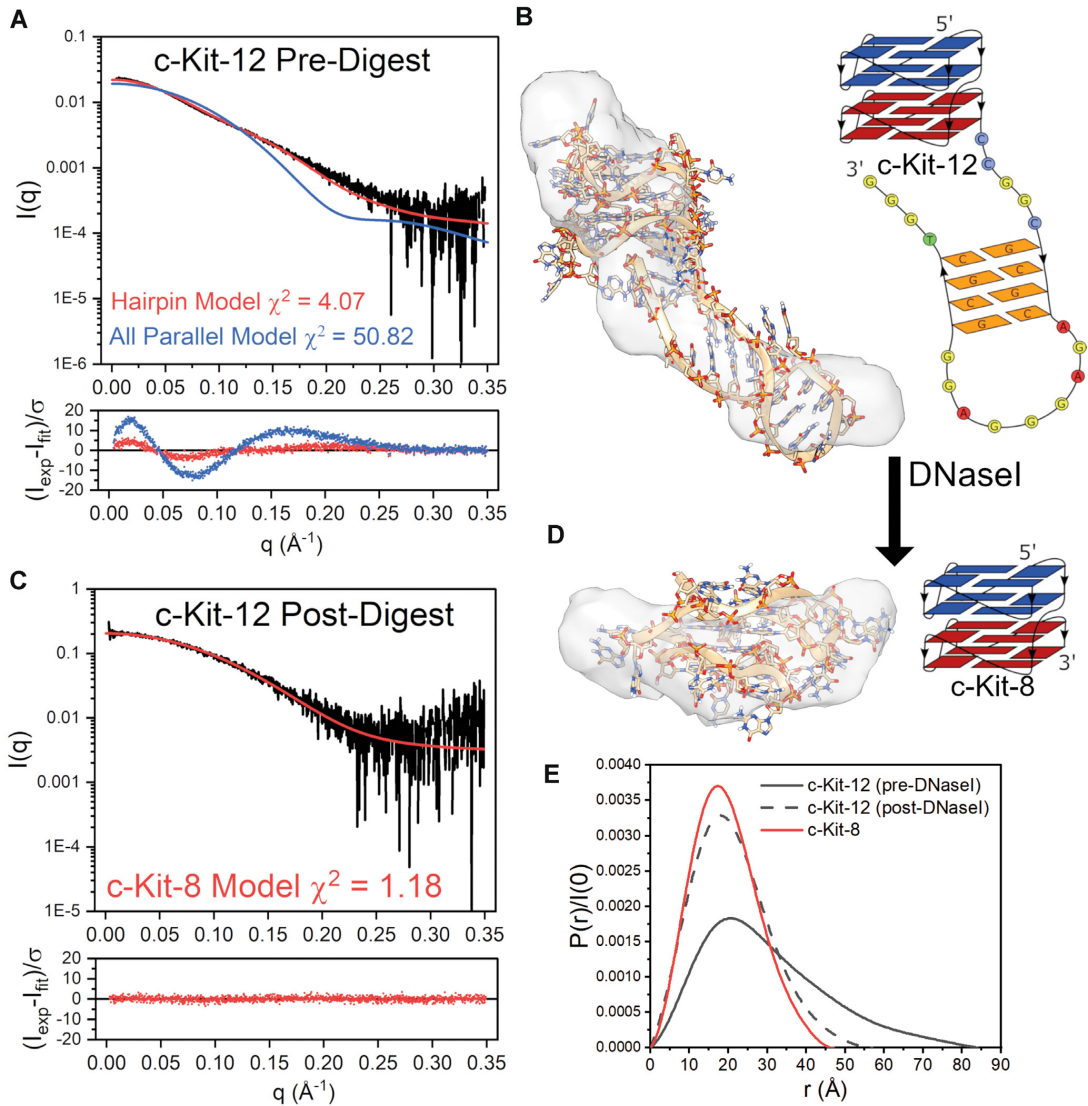
DNaseI-SEC-SAXS analysis of c-Kit-12 sequence. (**A**) SAXS scattering (Log-linear scale) of non-digested c-Kit-12 with overlaid fits of the all-parallel stacked or hairpin models and their weighted residuals. (**B**) Best-fitting hairpin model fit into *DAMMIF/N* refined scattering envelope along with its schematic representation. (**C**) SAXS scattering (Log-linear) of DNaseI-digested c-Kit-12 with overlaid fit of the c-Kit-8 four tetrad model (from Fig. 6) and its weighted residuals. (**D**) c-Kit-8 four stack model fit into the c-Kit-12 post-digestion *DAMMIF/N* refined scattering envelope along with its schematic representation. (**E**) Pair-distance distribution functions of c-Kit-12 pre-digestion (gray solid), c-Kit-12 post-digestion (gray dashed), and c-Kit-8 (red).

**Figure 9.**
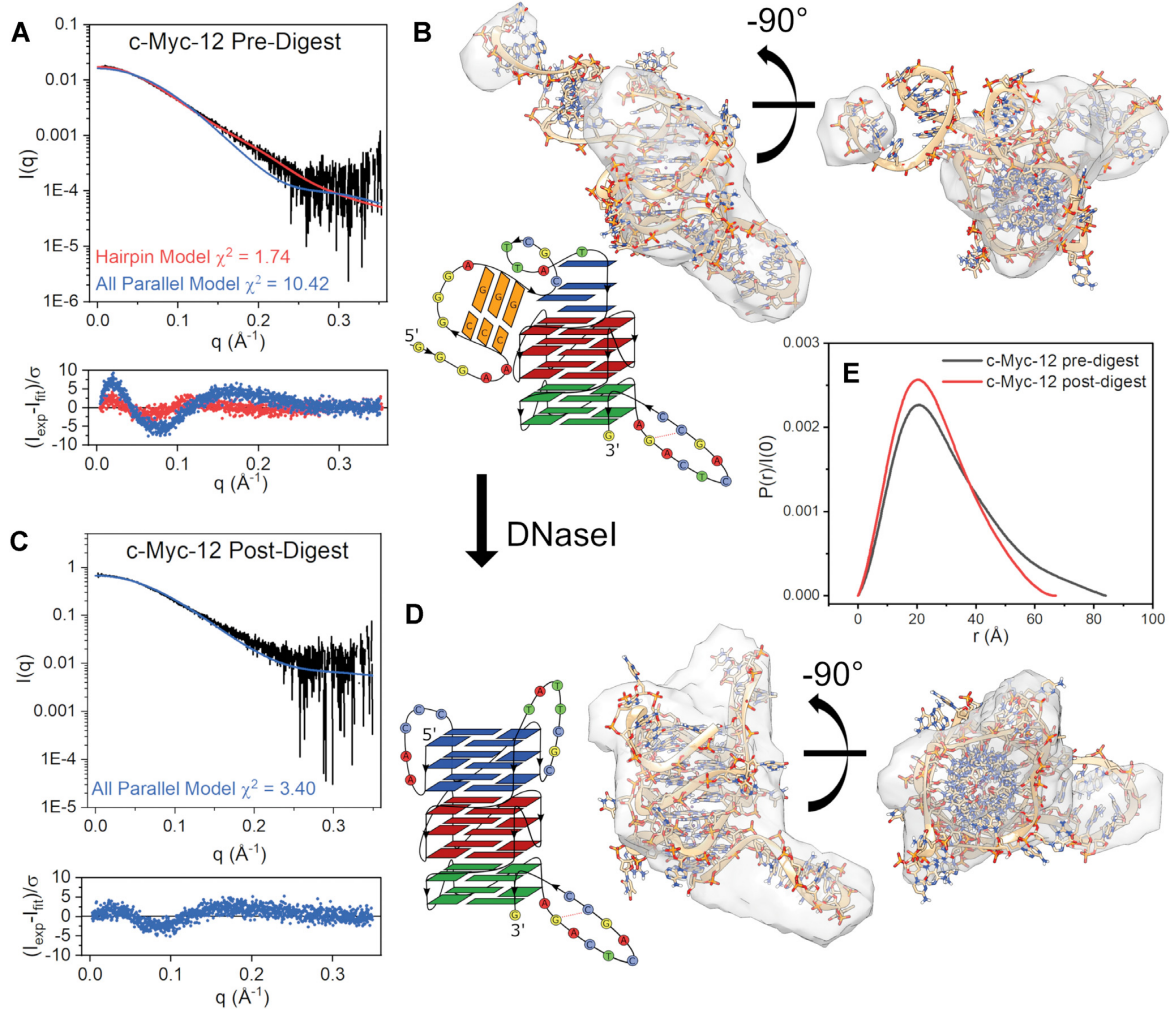
DNaseI-SEC-SAXS analysis of c-Myc-12 sequence. (**A**) SAXS scattering (Log-linear scale) of non-digested c-Myc-12 with overlaid fits of the all-parallel stacked or hairpin models and their weighted residuals. (**B**) Best-fitting hairpin model fit into the *DAMMIF/N* refined scattering envelope along with its schematic representation. (**C**) SAXS scattering (Log-linear) of DNaseI-digested c-Myc-12 sequence with overlaid fit of the all-parallel model (fully intact) and its weighted residuals. (**D**) c-Myc-12 all-parallel eight stack model fit into the post-digest *DAMMIF/N* refined scattering envelope and its schematic representation. (**E**) Pair-distance distribution functions of pre-digestion (gray) and post-digestion (red) particles.

To further investigate these possibilities, we collected SAXS data on the SEC-purified c-Kit-12 and c-Myc-12 DNaseI digestion products (Figures 8 and 9, respectively). We will first consider c-Kit-12. Figure 8 shows the scattering profiles of c-Kit-12 pre- and post-DNaseI treatment. In both cases the scattering data were determined to be homogeneous and free of interparticle artifacts, as shown by Guinier analysis of the low q region (Supplementary Figures S5 and S6). Figure 8A shows the scattering of c-Kit-12 pre-digest with overlaid *CRYSOL* fits of the stacked all-parallel model and a four stacked model with a hairpin incorporated as identified by Mfold. The hairpin model, although not in perfect agreement, is a much better representation of the scattering than the condensed globular, all-parallel model (not shown). This point is emphasized by the highly prolate space-filling envelope (Figure 8B). The χ^2^-value of 4.07 and poor envelope fit indicate that alternative hairpin-G4 isomers may also be contributing to the scattering, as this model is only one of multiple hairpin conformers identified as possible by Mfold. Further, the *ab initio* reconstruction is ambiguous based on AMBIMETER ambiguity score and the number of compatible shape categories (2.301 and 200, respectively) (Supplementary Table S3) and does not reflect the experimental sedimentation coefficient (Table 3). The envelope is shown to emphasize that the scattering is influenced by a highly extended species. More extensive flexible modeling approaches (e.g. EOM (118)) would likely be necessary to arrive at a better solution for the c-Kit-12 hairpin model. Figure 8C shows the post-DNaseI digestion scattering profile with the fit calculated from the four-tetrad c-Kit-8 model (same as in Figure 6). The fit is excellent as determined by its χ^2^-value of 1.2 and its normally distributed residuals. Visually, there is an imperfect fit with its *DAMMIF/N-refined* envelope (Figure 8D), and although the reconstruction is also potentially ambiguous (Supplementary Table S3), it does reflect well the measured *R_g_*, *D_max_*, and S_20,w_ Table 3. c-Kit-12’s change in size and shape from an extended prolate particle of *Dmax* = 86 Å and *R_g_* = 25.1 Å, to a very compact globular particle of *D_max_* = 54 Å and *R_g_* = 16.6 Å with similar dimensions (*D_max_*= 48 Å and *R_g_* = 15.0 Å) and sedimentation coefficient to c-Kit-8 (*S_20,w,c-Kit-8_* = 2.70 vs. *S_20,w,DNaseI_* = 2.85) is reflected in the pair distance distribution functions in Figure 8E.

The c-Myc-12 DNaseI cleavage product is not as easily interpreted as c-Kit-12’s (Figure 9). Figure 9A shows the pre-digest scattering of c-Myc-12 with overlaid *CRYSOL* fits of an all-parallel model and a model with a 3 base pair hairpin incorporated at the 5′ end. The hairpin model fits the scattering data well (χ^2^ = 1.74, Figure 9A), visually fits the *DAMMIF/N* envelope (Figure 9B), and has an *R_g_* that is in good agreement with what was originally measured (*R_g__,exp_*= 23.7 Å vs. *R_g,HP_* = 22.3 in Figure 5). However, based on our CD regression analysis, the incorporation of a B-form hairpin is expected to reduce the 260 nm CD signature by ∼200 Δϵ M^–1^cm^–1^_,_ as it disrupts the three tetrad G4 at the 5′ end. Coincidentally, this hairpin sequence is composed of contiguous GC base pairs that may exhibit a CD signature consistent with A-form DNA (peak at ∼264 nm, trough at ∼240 nm) (84) and resemble a parallel G4 CD signature, making it difficult to quantify its contribution (other than by using the NMR spectra in Supplementary Figure S28). However, the post-DNaseI SEC purified particle is much more globular in shape and has improved agreement with the all-parallel stacked model (Figure 9C and D). Taken with the increase in *S_20,w_* and lower *f/f_o_*, this suggests that the major form is compact and all parallel, with a minor extended hairpin species. We note that although the parallel stacked model fits well *visually* within its *ab initio* model, the reconstruction itself is potentially ambiguous and, further, both c-Myc-12 reconstructions had calculated *S_20,w_* values much lower than what was measured (Table 3). Also, because we don’t know with certainty which loop or hairpin nucleotides are cleaved by DNaseI, the *CRYSOL* calculation was performed using the intact parallel model, which could be one reason for the non-uniformly distributed residuals. Figure 9E shows the pair distance distribution function of pre- and post-digested c-Myc-12. The decrease in *D_max_* of and shift in *R_g_* from 23.7 down to 20.2 Å yields an improvement in c-Myc-12’s all-parallel model regression fit, (Figure 5 red arrow to blue). These collective results are consistent with a situation where coexistence of competing secondary structures can lead to convolution of CD, AUC, NMR, and scattering data, and so require an iterative integrative approach to parse out complex structural details.

## DISCUSSION

Our results show that an integrative structural biology approach can be used to effectively model the solution structures of higher-order G-quadruplexes, with a formal resolution of about 18 Å (107), but with derived atomistic models with greater detail. Structural models obtained for long *c-Myc*, *c-Kit* and *k-Ras* promoter sequences provide topological information not currently available from NMR or X-ray crystallography. While each of these promoter sequences fold into a unique structure, a common feature of these structures is the presence of contiguous parallel G4 units that interact to form unique interfacial pockets that might be targeted or recognized. Results from the longest *c-Myc* and *c-Kit* promoter sequences studied indicate that in addition to the G4 units, specific hairpin structures may also decorate the folded sequences to provide an even richer molecular terrain. These higher-ordered G4 structures all provide a rich array of potential drug binding sites.

**Figure 10.**
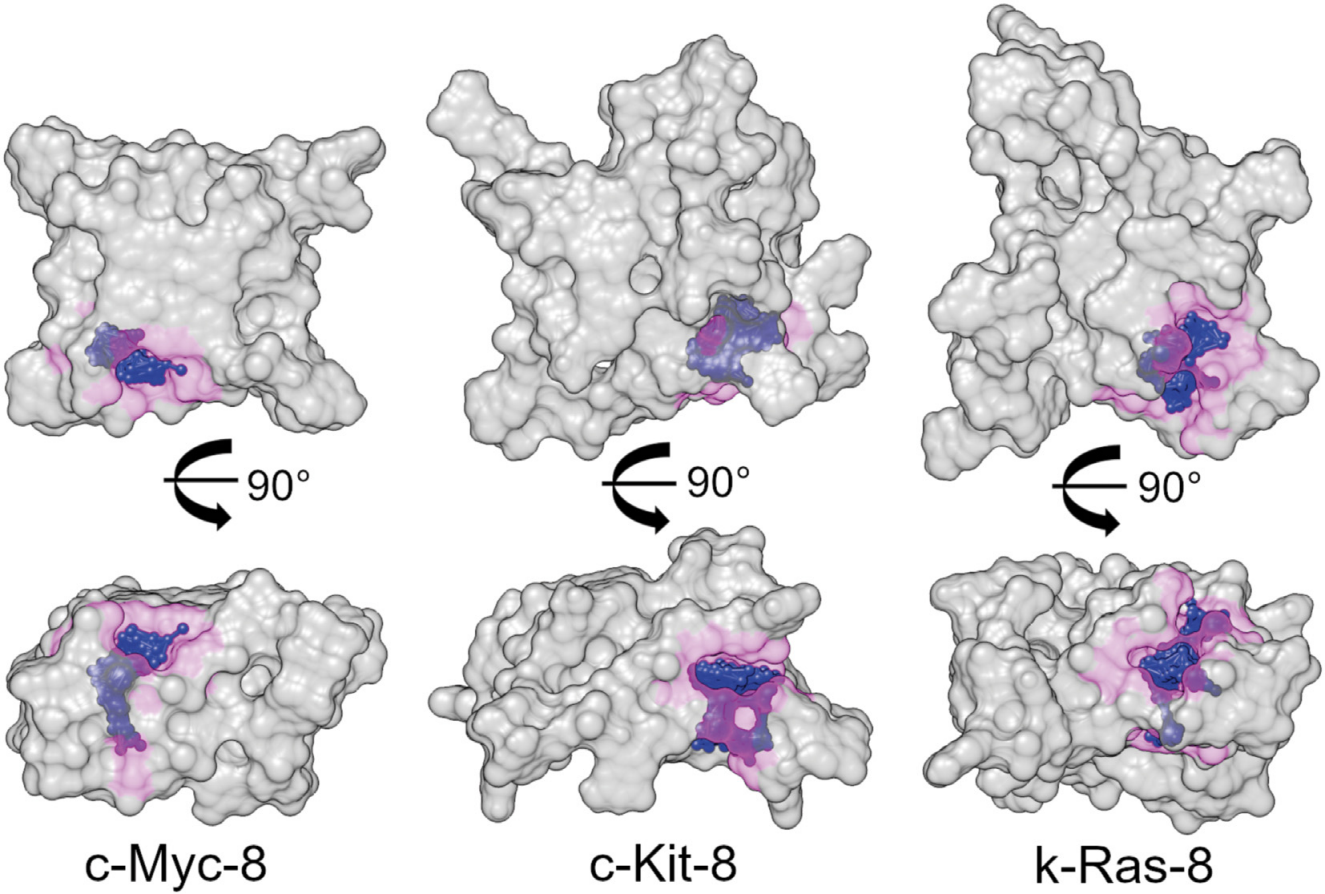
Space-filling representations of the c-Myc-8, c-Kit-8, and k-Ras-8 higher-order G-quadruplex models. The top row is looking down the central G4 stem and the bottom row is a side view. Magenta highlights a zone 4 Å from the ‘SITE’ ball and stick model (in blue) to emphasize the size of the predicted top-scoring binding sites.

Figure 10 shows surface renderings of the structures of c-Myc-8, c-Kit-8 and k-Ras-8. In this view, numerous unique topological features are evident, and these molecules at first glance look more like typical protein surfaces than canonical DNA. These folded G4 tertiary structures might be considered as DNA acting like a protein with respect to topological diversity. In order to illustrate the unique features of these higher-order G4 structures we used the program SiteMap (119) to generate information on the character and diversity of potential binding sites in the G4 structures we have determined (purple sites in Figure 10). SiteMap determines a ‘druggability score’ of a region of a protein or nucleic acid thereby providing a way to analyze potential binding sites and to predict target druggability. The calculated score characterizes a potential binding site with respect to: (i) the size of the site; (ii) the degrees of enclosure by the receptor and exposure to solvent; (iii) the tightness with which the site points interact with the receptor; (iv) the hydrophobic and hydrophilic character of the site and the balance between them and (v) the degree to which a ligand might donate or accept hydrogen bonds. For reference, the average scores for undruggable, difficult, and druggable sites are 0.63, 0.87 and 1.1, respectively (119). For the top binding sites in k-Ras-8, c-Myc-8, and c-Kit-8 the SiteMap druggability scores were 0.96, 0.87 and 1.00, respectively. For comparison, the binding sites of the c-Myc-derived monomeric, parallel, three tetrad-quadruplex (1XAV.pdb) four low affinity binding sites were found, with druggability scores ranging from 0.55 to 0.69. For the c-Myc-12 8-stack, c-Myc-12 hairpin, and c-Kit-12 hairpin structures, the top druggability scores were 0.94, 0.95, and 0.95, respectively, with multiple binding sites predicted (not shown). For further comparison, the known B-form minor groove binding site (289D.pdb), mithromycin A-form minor groove binding site (146D.pdb), and the daunomycin intercalation binding site (1d11.pdb) gave druggability scores of 0.99, 1.01, 0.85, respectively. Overall, this indicates that the higher-order 8- and 12-track structures have better and more druggable binding sites than simpler monomeric G4s. Figure 10 shows the top ranked (of several) sites in c-Myc-8, c-Kit-8 and k-Ras-8. These sites are unique to each sequence and structure, suggesting the possibility of targeting specific G4 promoters. However, targeting these sites experimentally is an effort beyond the scope of this work, but has begun and is an ongoing focus in our laboratory using the drug discovery platform we have described previously (120,121).

The coexistence of G-quadruplex isomers and competing secondary structures is often the rule of long G-rich sequences, and not the exception (20,22,122). The ISB approach (75), as demonstrated here, can satisfactorily characterize such complexities. Models of the 8-tract promoter sequences c-Myc-8 and c-Kit-8 (Figure 6) provide an excellent fit to all available biophysical measurements, although we caution that these models are only one solution to what could comprise an ensemble of G-register isomers. The k-Ras-8 model exemplifies this point (Figure 6, right panel). The parallel 4-stack model is self-consistent with spectroscopic and hydrodynamic measurements, yet its poor fit to its scattering data (e.g. skewing in its P(r) and Kratky profiles and poor χ^2^ fit) indicates that a population of structurally distinct isomers likely co-exist. Marquevielle et al. (123) recently reported on a 32-nt truncated version of the k-Ras-8 sequence. The authors show that their ‘K-RAS32R’ exists in a dynamic ensemble of two parallel G-register isomers that interconvert on a ms-timescale (123). This highlights the discriminatory power of combining hydrodynamics and spectroscopy with SAXS modeling.

The structural complexity of c-Myc-12 and c-Kit-12 are excellent examples of how powerful the ISB approach can be. In the former case, CD 264 nm values (Figure 2) indicate that c-Myc-12 is a fully stacked 8-tetrad parallel structure. However, its calculated radius of gyration was much smaller than measured (Figure 5). Integration of information from the Mfold DNA fold prediction server, physical theory, NMR (Supplemental Figure S28), and a DNaseI cleavage assay revealed that competing hairpin motifs (22) may be giving rise to a more extended prolate structure, as evidenced by the slightly higher frictional ratio, high *R_g_*, and a prolate space-filling envelope. Based on the low ratio of Watson-Crick imino peaks relative to the Hoogsteen peaks by proton NMR (1:18), the much lower than expected *S_20,w_* calculated for the extended *ab initio* models, and the nearly unchanged 264 nm CD magnitude after DNaseI digestion, it is reasonable to conclude that the major topology of c-Myc-12 is that of an all-parallel 8-tetrad system. Indeed, the all-parallel stacked atomistic model agrees well with the AUC sedimentation and SAXS scattering of the post-digestion sample (Figure 9 and Table 3). Conversely, c-Kit-12 was predicted to form extensive GC duplex features by Mfold, which was confirmed by DNaseI cleavage (Supplemental Figure S27). Hydrodynamic and scattering investigations of the post-digestion product reveals that c-Kit-12 likely forms the same parallel G4 as its truncated counterpart, c-Kit-8, with an extended hairpin loop as its major form, rather than a contiguous parallel stacked system. The biological importance of such a structure remains to be determined.

The sequence context in which we study extended sequences is also very important. We initially observed that promoter G4s preferentially adopt a parallel conformation, based on deposited structures and our prior investigations (52). Chen *et al.* recently reported that the addition of 5′-flanking non-guanine nucleotides induced conformational shifts from antiparallel or hybrid to parallel in ∼80% of the >300 sequences tested (70). We note that the sequences studied here do not have non-guanine 5′ flanking residues, but we subsequently tested the effects of adding 5′ nucleotides and found no substantial differences by CD (see Supplementary Figure S29). Recent studies also revealed that G4 motifs that are adjacent to one another tend to interact or stack rather than exist as a ‘beads-on-a-string’. We (52) and others (51) have shown that the hTERT core promoter region forms a three parallel stacked assembly and, importantly, that the internal G4 region only forms in the presence of one or both of the outer G4s. Two independent studies of the c-Kit proximal promoter region (non-overlapping with the c-Kit-8 or –12 sequences studied here) have shown a similar phenomenon, although no atomistic models were proposed. In the first study Rigo and Sissi (53) used CD and melting studies to show that the kit2-kit* higher-order quadruplex exhibits a thermodynamic and structural cross-talk between the two G4 subunits (53). A later study Ducani et al. (54) investigated a sequence containing all three of the previously reported monomeric G4s, kit2-kit*-kit1, as well as mutated combinations thereof. They report that the kit* sequence does not form an antiparallel G4 in the presence of kit2, but rather is stabilized as a parallel stacked higher-order complex (54). Importantly, two-tetrad parallel DNA G4s are unstable without external stabilizing forces, such as hairpin loops or stacking interactions (124), highlighting the importance of developing integrative approaches to tackle extended sequences.

As indicated in the introduction, there may be biological advantages in forming higher-order promoter G4s. Promoter G4s have long been suspected to exert regulatory functions on gene transcription based on genetic conservation, prevalence in nucleosome depleted regions, and their non-random distribution in gene promoters (4,125). Recently, state-of-the-art sequencing has revealed that promoter G4s act as transcription factor (TF) hubs, and that small molecules can effectively displace TF binding (9). However, biological studies of higher-order G4s are sparse. We have previously shown that the hTERT core promoter sequence adopts a higher-order three-stack parallel G4 (52). The Costello lab (126) has shown that the two frequent G > A hTERT core promoter mutations, both of which reside in the middle G4 G-tracts, lead to a TF profile switch that significantly increases promoter activity. Although they show that these specific mutations create new TF binding motifs, it is very difficult to parse out contributions from putative G4 secondary structure for this very reason. A more direct dissection of a higher-order G4 was recently conducted by the Terenzi lab (54) using the c-Kit promoter. By systematically mutating out each G4 motif with poly adenines, the authors show that promoter activity is directly modulated by the status of higher-order G4 formation. Moreover, their study reveals that the c-Kit G4s do not ‘titrate’ the promoter response or behave as simple switches, rather, they may instead ‘code’ for a particular promoter response. Collectively, these studies suggest that higher-order G4 promoters offer an unrivaled 3-dimensional landscape that is critically linked to promoter activity.

## CONCLUSION

The promoter regions of oncogenes have long sequences with the potential to form multiple quadruplexes, yet this complexity has largely been intractable to modeling. Structural biologists have instead, for expediency, focused primarily on shorter sequences that fold into a single monomeric G4. The integrated structural biology approach provides the means for studying the structures of the more relevant extended sequences. Every such sequence studied to date shows that more complex higher-order G4 structures readily form, featuring contiguous G4 units, longer loop structures and in some cases coexisting duplex hairpins. We suggest that these more complex assemblies may be the more important regulators of promoter function in ways that are yet to be defined.

## DATA AVAILABILITY

Small-angle X-ray scattering data and models, where applicable, have been deposited in the publicly accessible Small Angle Scattering Biological Data Bank (https://www.sasbdb.org/) under the names (IDs): c-Myc-8 (SASDMJ6), c-Myc-12 (SASDMM6), c-Myc-12 post-DNaseI (SASDM36), c-Kit-8 (SASDMK6), c-Kit-12 (SASDMN6), c-Kit-12 post-DNaseI (SASDM46), k-Ras-8 (SASDML6), 2KZD (SASDM56), 201D (SASDM76), 6GH0 (SASDM86), 2GKU (SASDM96), 2JSL (SASDKF3), 5I2V (SASDMA6), 6L92 (SASDMB6), 2KQG (SASDMC6), 2LBY (SASDMD6), 6NEB (SASDME6), 1XAV (SASDMF6), 2KZE (SASDM66), 5CMX (SASDMG6), 2M27 (SASDMH6).

## Supplementary Material

gkac182_Supplemental_File
