# Supplementary material for: Long promoter sequences form higher-order G-quadruplexes: an integrative structural biology study of c-Myc, k-Ras and c-Kit promoter sequences
Source: Nucleic Acids Res. 2022 Mar 23;50(7):4127–47. doi: 10.1093/nar/gkac182 (PMC9023277; doi:10.1093/nar/gkac182)
Supplement: gkac182_Supplemental_File [file gkac182_supplemental_file.pdf]

Supplemental Material:

Long promoter sequences form higher-order G-quadruplexes: An Integrative structural biology study of *c-Myc*, *k-Ras*, and *c-Kit* promoter sequences

Robert C. Monsen[1], Lynn W. DeLeeuw[1], William L. Dean[1], Robert D. Gray[1], Srinivas Chakravarthy[2], Jesse B. Hopkins[2], Jonathan B. Chaires*[1,3,4], and John O. Trent*[1,3,4]

[1]UofL Health Brown Cancer Center, University of Louisville, 505 S. Hancock St., Louisville, KY 40202 USA
[2]The Biophysics Collaborative Access Team (BioCAT), Department of Biological, Chemical, and Physical Sciences, Illinois Institute of Technology, Chicago, IL 60616, USA
[3]Department of Medicine, University of Louisville, Louisville, KY 40202, USA
[4]Department of Biochemistry and Molecular Genetics, University of Louisville, Louisville, KY 40202, USA

Contents:

Table S1. Qualitative literature summary of CD signatures for short promoter sequences with or without 5' flanking residues. Red highlights the 5' flanked sequences that aren't parallel. Black indicates sequences that are not 5' flanked that are not parallel. Blue highlights 5' flanked sequences with at least partial parallel characteristics.

Table S2. *Quadparser* analysis of promoter potential quadruplex sequences (PQS) in the human genome.

Table S3. Tabulated small-angle X-ray scattering data acquisition, reduction, analysis, results and SASBDB identifiers.

Table S4. Tabulated small-angle X-ray scattering data acquisition, reduction, analysis, results and SASBDB identifiers of oligonucleotides from the PDB used in CRYSOL R_g regression.

Table S5. Tabulated hydrodynamic results from SAXS-derived ab initio bead models.

S1. SAXS data summary for c-Myc-8. a) Series intensity (blue, left axis) vs. frame, and, if available, Rg vs. frame (red, right axis). Green shaded regions are buffer regions, purple shaded regions are sample regions. b) Scattering profile(s) on a log-lin scale. c) Guinier fit(s) (top) and fit residuals (bottom). d) Normalized Kratky plot. Dashed lines show where a globular system would peak. Figure created in BioXTAS RAW v2.1.1.

S2. SAXS data summary for c-Myc-12. a) Series intensity (blue, left axis) vs. frame, and, if available, Rg vs. frame (red, right axis). Green shaded regions are buffer regions, purple shaded regions are sample regions. b) Scattering profile(s) on a log-lin scale. c) Guinier fit(s) (top) and fit residuals (bottom). d) Normalized Kratky plot. Dashed lines show where a globular system would peak. Figure created in BioXTAS RAW v2.1.1.

S3. SAXS data summary for c-Myc-12 Post-DNaseI. a) Series intensity (blue, left axis) vs. frame, and, if available, Rg vs. frame (red, right axis). Green shaded regions are buffer regions, purple shaded regions are sample regions. b) Scattering profile(s) on a log-lin scale. c) Guinier fit(s) (top) and fit residuals (bottom). d) Normalized Kratky plot. Dashed lines show where a globular system would peak. e) P(r) function(s), normalized by I(0). Figure created in BioXTAS RAW v2.1.1.

S4. SAXS data summary for c-Kit-8. a) Series intensity (blue, left axis) vs. frame, and, if available, Rg vs. frame (red, right axis). Green shaded regions are buffer regions, purple shaded regions are sample regions. b) Scattering profile(s) on a log-lin scale. c) Guinier fit(s) (top) and fit residuals (bottom). d) Normalized Kratky plot. Dashed lines show where a globular system would peak. Figure created in BioXTAS RAW v2.1.1.

S5. SAXS data summary for c-Kit-12. a) Series intensity (blue, left axis) vs. frame, and, if available, Rg vs. frame (red, right axis). Green shaded regions are buffer regions, purple shaded regions are sample regions. b) Scattering profile(s) on a log-lin scale. c) Guinier fit(s) (top) and fit residuals (bottom). d) Normalized Kratky plot. Dashed lines show where a globular system would peak. Figure created in BioXTAS RAW v2.1.1.

S6. SAXS data summary for c-Kit-12 Post-DNaseI. a) Series intensity (blue, left axis) vs. frame, and, if available, Rg vs. frame (red, right axis). Green shaded regions are buffer regions, purple shaded regions are sample regions. b) Scattering profile(s) on a log-lin scale. c) Guinier fit(s) (top) and fit residuals (bottom). d) Normalized Kratky plot. e) Normalized Pr distribution. Dashed lines show where a globular system would peak. Figure created in BioXTAS RAW v2.1.1.

S7. SAXS data summary for k-Ras-8 Post-DNaseI. a) Series intensity (blue, left axis) vs. frame, and, if available, Rg vs. frame (red, right axis). Green shaded regions are buffer regions, purple shaded regions are sample regions. b) Scattering profile(s) on a log-lin scale. c) Guinier fit(s) (top) and fit residuals (bottom). d) Normalized Kratky plot. Dashed lines show where a globular system would peak. Figure created in BioXTAS RAW v2.1.1.

S8. SAXS data summary for 1XAV. a) Series intensity (blue, left axis) vs. frame, and, if available, Rg vs. frame (red, right axis). Green shaded regions are buffer regions, purple shaded regions are sample regions. b) Scattering profile(s) on a log-lin scale. c) Guinier fit(s) (top) and fit residuals (bottom). d) Normalized Kratky plot. Dashed lines show where a globular system would peak. e) P(r) function(s), normalized by I(0). Figure created in BioXTAS RAW v2.1.1.

S9. SAXS data summary for 2GKU. a) Series intensity (blue, left axis) vs. frame, and, if available, Rg vs. frame (red, right axis). Green shaded regions are buffer regions, purple shaded regions are sample regions. b) Scattering profile(s) on a log-lin scale. c) Guinier fit(s) (top) and fit residuals (bottom). d) Normalized Kratky plot. Dashed lines show where a globular system would peak. e) P(r) function(s), normalized by I(0). Figure created in BioXTAS RAW v2.1.1.

S10. SAXS data summary for 2JSL. a) Series intensity (blue, left axis) vs. frame, and, if available, Rg vs. frame (red, right axis). Green shaded regions are buffer regions, purple shaded regions are sample regions. b) Scattering profile(s) on a log-lin scale. c) Guinier fit(s) (top) and fit residuals (bottom). d) Normalized Kratky plot. Dashed lines show where a globular system would peak. e) P(r) function(s), normalized by I(0). Figure created in BioXTAS RAW v2.1.1.

S11. SAXS data summary for 2KQG. a) Series intensity (blue, left axis) vs. frame, and, if available, Rg vs. frame (red, right axis). Green shaded regions are buffer regions, purple shaded regions are sample regions. b) Scattering profile(s) on a log-lin scale. c) Guinier fit(s) (top) and fit residuals (bottom). d) Normalized Kratky plot. Dashed lines show where a globular system would peak. e) P(r) function(s), normalized by I(0). Green (029_2), orange (029_1), and blue (029_0) species are the result of deconvolution using

evolving factor analysis(1). The monomeric species, as determined by approximate molecular weight (Vc), is shown in green. Figure created in BioXTAS RAW v2.1.1.

S12. SAXS data summary for 2KZD. a) Series intensity (blue, left axis) vs. frame, and, if available, Rg vs. frame (red, right axis). Green shaded regions are buffer regions, purple shaded regions are sample regions. b) Scattering profile(s) on a log-lin scale. c) Guinier fit(s) (top) and fit residuals (bottom). d) Normalized Kratky plot. Dashed lines show where a globular system would peak. e) P(r) function(s), normalized by I(0). Figure created in BioXTAS RAW v2.1.1.

S13. SAXS data summary for 2KZE. a) Series intensity (blue, left axis) vs. frame, and, if available, Rg vs. frame (red, right axis). Green shaded regions are buffer regions, purple shaded regions are sample regions. b) Scattering profile(s) on a log-lin scale. c) Guinier fit(s) (top) and fit residuals (bottom). d) Normalized Kratky plot. Dashed lines show where a globular system would peak. e) P(r) function(s), normalized by I(0). Figure created in BioXTAS RAW v2.1.1.

S14. SAXS data summary for 2LBY. a) Series intensity (blue, left axis) vs. frame, and, if available, Rg vs. frame (red, right axis). Green shaded regions are buffer regions, purple shaded regions are sample regions. b) Scattering profile(s) on a log-lin scale. c) Guinier fit(s) (top) and fit residuals (bottom). d) Normalized Kratky plot. Dashed lines show where a globular system would peak. e) P(r) function(s), normalized by I(0). Green (027_2), orange (027_1), and blue (027_0) species are the result of deconvolution using evolving factor analysis(1). The monomeric species, as determined by approximate molecular weight (Vc), is shown in green. Figure created in BioXTAS RAW v2.1.1.

S15. SAXS data summary for 2M27. a) Series intensity (blue, left axis) vs. frame, and, if available, Rg vs. frame (red, right axis). Green shaded regions are buffer regions, purple shaded regions are sample regions. b) Scattering profile(s) on a log-lin scale. c) Guinier fit(s) (top) and fit residuals (bottom). d) Normalized Kratky plot. Dashed lines show where a globular system would peak. e) P(r) function(s), normalized by I(0). Orange (031_1), and blue (031_0) species are the result of deconvolution using evolving factor analysis(1). The monomeric species, as determined by approximate molecular weight (Vc), is shown in orange. Figure created in BioXTAS RAW v2.1.1.

S16. SAXS data summary for 5CMX. a) Series intensity (blue, left axis) vs. frame, and, if available, Rg vs. frame (red, right axis). Green shaded regions are buffer regions, purple shaded regions are sample regions. b) Scattering profile(s) on a log-lin scale. c) Guinier fit(s) (top) and fit residuals (bottom). d) Normalized Kratky plot. Dashed lines show where a globular system would peak. e) P(r) function(s), normalized by I(0). Figure created in BioXTAS RAW v2.1.1.

S17. SAXS data summary for 5I2V. a) Series intensity (blue, left axis) vs. frame, and, if available, Rg vs. frame (red, right axis). Green shaded regions are buffer regions, purple shaded regions are sample regions. b) Scattering profile(s) on a log-lin scale. c) Guinier fit(s) (top) and fit residuals (bottom). d) Normalized Kratky plot. Dashed lines show where a globular system would peak. e) P(r) function(s), normalized by I(0). Figure created in BioXTAS RAW v2.1.1.

S18. SAXS data summary for 6GH0. a) Series intensity (blue, left axis) vs. frame, and, if available, Rg vs. frame (red, right axis). Green shaded regions are buffer regions, purple shaded regions are sample regions. b) Scattering profile(s) on a log-lin scale. c) Guinier fit(s) (top) and fit residuals (bottom). d) Normalized Kratky plot. Dashed lines show where a globular system would peak. e) P(r) function(s), normalized by

I(0). Green (035_2), orange (031_1), and blue (031_0) species are the result of deconvolution using evolving factor analysis(1). The monomeric species, as determined by approximate molecular weight (Vc), is shown in green. Figure created in BioXTAS RAW v2.1.1.

S19. SAXS data summary for 6L92. a) Series intensity (blue, left axis) vs. frame, and, if available, Rg vs. frame (red, right axis). Green shaded regions are buffer regions, purple shaded regions are sample regions. b) Scattering profile(s) on a log-lin scale. c) Guinier fit(s) (top) and fit residuals (bottom). d) Normalized Kratky plot. Dashed lines show where a globular system would peak. e) P(r) function(s), normalized by I(0). Orange (031_1), and blue (031_0) species are the result of deconvolution using evolving factor analysis(1). The monomeric species, as determined by approximate molecular weight (Vc), is shown in orange. Figure created in BioXTAS RAW v2.1.1.

S20. SAXS data summary for 6NEB. a) Series intensity (blue, left axis) vs. frame, and, if available, Rg vs. frame (red, right axis). Green shaded regions are buffer regions, purple shaded regions are sample regions. b) Scattering profile(s) on a log-lin scale. c) Guinier fit(s) (top) and fit residuals (bottom). d) Normalized Kratky plot. Dashed lines show where a globular system would peak. e) P(r) function(s), normalized by I(0). Figure created in BioXTAS RAW v2.1.1.

S21. SAXS data summary for 201D. a) Series intensity (blue, left axis) vs. frame, and, if available, Rg vs. frame (red, right axis). Green shaded regions are buffer regions, purple shaded regions are sample regions. b) Scattering profile(s) on a log-lin scale. c) Guinier fit(s) (top) and fit residuals (bottom). d) Normalized Kratky plot. Dashed lines show where a globular system would peak. e) P(r) function(s), normalized by I(0). Figure created in BioXTAS RAW v2.1.1.

Figure S22. CRYSOL v2.8.3 fits of the 14 G4s from the PDB used in building the R_g regression. PDB ID's are: (a) 1XAV, (b) 2GKU, (c) 2JSL, (d) 2KQG, (e) 2KZD, (f) 2KZE, (g) 2LBY, (h) 2M27, (i) 5CMX, (j) 5I2V, (k) 6GH0, (l) 6L92, (m) 6NEB, (n) 201D. Tabulated results for the fits are given in Table S4. Figure created in BioXTAS RAW v2.1.1.

Figure S23. Mfold analysis of cMyc-8.

Figure S24. Mfold analysis of cKit-8.

Figure S25. Mfold analysis of kRas-8.

Figure S26. Mfold analysis of cMyc-12.

Figure S27. Mfold analysis of cKit-12.

Figure S28. 1D proton NMR analysis of MYC-12. Red lines are integrations of the Watson-Crick and G-quadruplex imino proton regions, yielding a ratio of ~18:1 G-tetrad imino protons to duplex (Watson-Crick face) imino protons.

Figure S29. CD spectra of extended promoter sequences with 5' flanking nucleotides.

Gene/Name	Spectrum	5' flanked?	Length (nt)	Ref
BCL-2	parallel	Y	39	(2)
VEGF	parallel	Y	25	(3)
Hif1a	parallel	Y	31	(4)
c-MYB	parallel	N	42	(5)
PDGF-A	parallel	Y	48	(6)
AR	parallel	N	33	(7)
RET	parallel	Y	20	(8)
EGFR	mixture	N	30	(9)
PARP1	antiparallel/hybrid	Y	23	(10)
WNT1	parallel/mix	N	22	(11,12)
MyoD	parallel	Y	26	(13)
CLIC4	antiparallel/hybrid	N	31-33	(14)
RAD17	hybrid	Y	24	(15)
RAD17	parallel	N	19	(15)
PCNA	parallel	Y	26-34	(16)
Yin Yang1	parallel	N	62	(17)
HSP90	Parallel	Y	27	(18)
HSP90	Parallel/mix	N	21	(18)
NPGPx	parallel + hairpin	Y	60	(19)
NPGPx-32	parallel	Y	32	(19)
UCP1	parallel	Y	24	(20)
MEST	parallel	Y	51	(21)
PCGF3	parallel/mix	N	21	(22)
TH49	parallel/mix	N	45	(23)
ALOX5	parallel	Y	43	(24)
VEGFR-2	parallel/mix	N	23	(25)
TKQ1	antiparallel/hybrid	N	21	(26)
TKQ2	antiparallel/hybrid	N	22	(26)
NRF2	parallel	N	24	(27)
MET	parallel	Y	23	(28)
c-MYC Pu19	parallel	Y	19	(29)
cKIT21	parallel	Y	21	(30)
cKIT18T	parallel	Y	18	(30)
cKIT21T	parallel	Y	21	(30)
FGFR2 S1	parallel	N	24	(31)
FGFR2 S2	parallel/mix	N	35	(31)
FGFR2 S3	parallel	N	22	(31)

Table S1. Qualitative literature summary of CD signatures for short promoter sequences with or without 5’ flanking residues. Red highlights the 5’ flanked sequences that aren’t parallel. Black indicates sequences that are not 5’ flanked that are not parallel. Blue highlights 5’ flanked sequences with at least partial parallel characteristics.

	2G, 4 runs				3G, 4 runs			
	1-7 loop	1-8 loop	1-9 loop	1-10 loop	1-7 loop	1-8 loop	1-9 loop	1-10 loop
Occurrence -499 to 100	166808	178795	187996	194846	21112	24292	27337	29992
Gene -499 to 100	28160	28430	28660	28803	12752	13992	15077	15951
% promoters	95.14	96.05	96.83	97.31	43.08	47.27	50.94	53.89
Occurrence -750 to 100	216564	233501	247240	257082	25716	29705	33552	36968
Gene -750 to 100	28674	28880	29052	29160	14051	15293	16383	17259
% promoters	96.88	97.57	98.16	98.52	47.47	51.67	55.35	58.31

	2G, 8 runs				3G, 8 runs			
	1-7 loop	1-8 loop	1-9 loop	1-10 loop	1-7 loop	1-8 loop	1-9 loop	1-10 loop
Occurrence -499 to 100	33551	40663	47181	53587	1508	1982	2469	3007
Gene -499 to 100	16996	18896	20451	21730	1380	1788	2220	2641
% promoters	57.42	63.8	69.1	73.4	4.7	6	7.5	8.9
Occurrence -750 to 100	40653	49566	58048	66307	1754	2301	2901	3521
Gene -750 to 100	18136	19972	21560	22782	1564	2014	2514	2987
% promoters	61.27	67.48	72.84	76.97	5.28	6.8	8.49	10.09

	2G, 12 runs				3G, 12 runs			
	1-7 loop	1-8 loop	1-9 loop	1-10 loop	1-7 loop	1-8 loop	1-9 loop	1-10 loop
Occurrence -499 to 100	10153	13327	16629	20012	233	351	470	600
Gene -499 to 100	7653	9548	11342	13058	228	339	448	568
% promoters	25.86	32.26	38.32	44.12	0.77	1.15	1.51	1.92
Occurrence -750 to 100	12001	15874	19964	24155	269	410	545	697
Gene -750 to 100	8462	10507	12409	14188	261	393	513	651
% promoters	28.59	35.5	41.93	47.94	0.88	1.33	1.73	2.2

Table S2. *Quadparser* analysis of promoter PQS in the human genome.

(a) Sample Details.

	c-Myc-8	c-Myc-12	c-Myc-12 Post-DNaseI	c-Kit-8	c-Kit-12	c-Kit-12 Post-DNaseI	k-Ras-8
Organism	synthetic	synthetic	synthetic	synthetic	synthetic	synthetic	synthetic
Extinction coefficient (nearest neighbor approximation) (M^{-1} cm^{-1})	354900	731000		381800	626800	n/a	445300
vbar (cm^3/g) (estimate)	0.55	0.55	0.55	0.55	0.55	0.55	0.55
M from chemical composition (Da)	10976	23218	20600*	12143	20349	14700*	13755
SEC-SAXS column, 10 x 300 Superdex 75							
Loading concentration (mg/ml)	6.6	7.4	2.3	8.9	4.3	1.4	6.5
Injection volume (µl)	285	330	270	275	340	230	310
Flow rate (ml/min)	0.7	0.7	0.6	0.7	0.7	0.6	0.7
Solvent (solvent blanks taken from SEC flowthrough prior to elution of protein)	BPEK, 185 mM KCl, pH 7.2	BPEK, 185 mM KCl, pH 7.2	BPEK, 185 mM KCl, pH 7.2	BPEK, 185 mM KCl, pH 7.2	BPEK, 185 mM KCl, pH 7.2	BPEK, 185 mM KCl, pH 7.2	BPEK, 185 mM KCl, pH 7.2

*Measured by AUC-SV

(b) SAXS data-collection parameters.

Instrument/data processing	BioCAT facility at the Advanced Photon Source beamline 18ID with Pilatus3 X 1M (Dectris) detector
Wavelength (Å)	1.033
Beam size (µm)	150 (h) x 25 (v)
Camera length (m)	3.628
q measurement range ($Å^{-1}$)	0.0044-0.35
Absolute scaling method	Glassy Carbon, NIST SRM 3600
Normalization	To incident intensity, by ion chamber counter
Monitoring for radiation damage	Automated frame-by-frame comparison of relevant regions
Exposure time, number of exposures	0.5 s exposure time with a 2s total exposure period (0.5 s on, 1.5 s off) of entire SEC elution.
Sample configuration	SEC-SAXS. Size separation by an AKTA Pure with a Superdex 200 Increase 10/300 GL column and sheath flow cell(32) to minimize sample damage. SAXS data measured in a 1 mm ID quartz capillary with effective path length 0.542 mm.
Sample temperature (°C)	R.T.

(c) Software employed for SAXS data reduction, analysis, and interpretation.

SAXS data reduction	Radial averaging; frame comparison, averaging, and subtraction done using BioXTAS RAW 1.6.3 (Hopkins et al. 2017(33))
Extinction coefficient estimate	Nearest neighbor approximation
Basic analyses: Guinier, P(r), Vp	Guinier fit, P(r), Kratky analysis, and molecular weight using BioXTAS RAW 2.1.1 and GNOM(33,34)
Shape/bead modelling	DAMMIF/DAMAVER/DAMCLUST/DAMMIN in BioXTAS RAW 2.1.1 (35-38)
Atomic structure modelling	CRYSOL v2.8.3 (39)
Three-dimensional graphic model representations	UCSF Chimera v1.12

(d) Structural parameters.

Guinier analysis	c-Myc-8	c-Myc-12	c-Myc-12 Post-DNaseI	c-Kit-8	c-Kit-12	c-Kit-12 Post-DNaseI	k-Ras-8
I(0) (cm^{-1})	0.0146 ± 0.0000128	0.0177 ± 0.0000269	0.6840 ± 0.00118	0.022 ± 0.000018	2.2415 ± 0.00162	0.2075 ± 0.000666	0.0214 ± 0.000018
Rg (Å)	14.35 ± 0.03	22.84 ± 0.06	19.45 ± 0.08	14.89 ± 0.02	24.29 ± 0.04	15.78 ± 0.10	16.77 ± 0.03
qmin*Rg	0.06	0.1	0.06	0.06	0.1	0.05	0.13
qmax*Rg	1.3	1.3	1.16	1.3	1.09	1.35	1.3
χ^2	0.99	0.99	0.98	0.99	1.00	0.93	1.00
M (Da) from Vc (ratio to predicted)	13300 (1.21)	24500 (1.06)	20700 (1.00)	14700 (1.21)	21700 (1.07)	15300 (1.04)	16100 (1.17)
P(r) analysis							
I(0) (cm^{-1})	0.0146 ± 0.0000132	0.0179 ± 0.0000317	0.6918 ± 0.0011	0.022 ± 0.00002	2.2550 ± 0.00154	0.2096 ± 0.0006	0.0214 ± 0.0000198
Rg (Å)	14.42 ± 0.0229	23.70± 0.07	20.33 ± 0.69	14.95 ± 0.02	25.10 ± 0.03	16.55 ± 0.06	16.98 ± 0.03
Dmax (Å)	46	84	65	48	86	54	56
χ^2	1.03	1.20	1.50	1.01	1.79	1.44	1.01
Porod volume ($Å^{-3}$) (ratio Vp/calculated M)	9500 (0.87)	27400 (1.18)	19600 (0.95)	11400 (0.94)	23900 (1.17)	12700 (0.86)	14600 (1.06)

(e) Shape model-fitting results

	c-Myc-8	c-Myc-12	c-Myc-12 Post-DNaseI	c-Kit-8	c-Kit-12	c-Kit-12 Post-DNaseI	k-Ras-8
AMBIMETER (default parameters)							
Number of compatible shape categories, (Ambiguity score)	10, (1.000)	51, (1.708)	183, (2.262)	12, (1.079)	200, (2.301)	142, (2.152)	34, (1.531)
3D reconstruction	potentially unique	potentially ambiguous	potentially ambiguous	potentially unique	potentially ambiguous	potentially ambiguous	potentially ambiguous
DAMMIF (20x, slow mode), DAMAVER, DAMCLUST, DAMMIN (refinement)							
q range for fitting ($Å^{-1}$)	0.01-0.30	0.007-0.30	0.006-0.30	0.006-0.30	0.004-0.30	0.003-0.30	0.007-0.30
Symmetry, anisotropy assumptions	P1, none	P1, none	P1, none	P1, none	P1, none	P1, none	P1, none
Mean NSD ± STDEV	0.534 ± 0.017	1.688 ± 0.287	0.828 ± 0.087	0.583 ± 0.063	1.065 ± 0.096	0.799 ± 0.141	0.895 ± 0.17
# Rejected models	1	2	1	1	2	1	2
# Clusters	11	9	4	4	5	4	6
Ensemble Resolution (Å)	17 ± 2	37 ± 3	26 ± 2	18 ± 2	30 ± 2	23 ± 2	23 ± 2
χ^2	1.027	1.192	1.505	1.017	1.787	1.434	1.01
Rg (Å)	14.43	23.74	20.42	14.97	25.12	16.575	16.96
Dmax (Å)	48	88	70	50	88	58	56
MW from Vc (kDa)	10.1	23.7	20.1	11.35	21.33	13.01	13.14

(f) Atomistic modelling.

Model	c-Myc-8	c-Myc-12 (Hairpin)	c-Myc-12 (All Parallel)	c-Kit-8	c-Kit-12 (Hairpin)	c-Kit-8	k-Ras-8
q range for all modelling	0.01-0.35	0.007-0.353	0.006-0.3491	0.006-0.35	0.004-0.353	0.003-0.3491	0.007-0.35
CRYSOL v2.8.3							
χ^2	1.67	1.74	3.4	1.66	4.07	1.18	5.6

Calculated Rg (Å)	14.14	22.28	19.04	14.7	23.39	15.52	15.66
Vol (Å^3), Ra (Å), Dro (e Å^{-3})	10341, 1.680, 0.075	17910, 1.400, 0.045	19937, 1.800, 0.045	11026, 1.800, 0.040	21061, 1.500, 0.075	13196, 1.620, 0.075	12136, 1.800, 0.060
(g) SASBDB IDs for data and models.							
ID	SASDMJ6	SASDMM6	SASDM36	SASDMK6	SASDMN6	SASDM46	SASDML6

Table S3. Tabulated small-angle X-ray scattering data acquisition, reduction, analysis, results and SASBDB identifiers.

(a) SAXS Sample Details.

	2KZD	201D	6GH0	2GKU	2JSL	5I2V	6L92	2KQG	2LBY	6NEB	1XAV	2KZE	5CMX	2M27
Organism	synthetic	synthetic	synthetic	synthetic	synthetic	synthetic	synthetic	synthetic	synthetic	synthetic	synthetic	synthetic	synthetic	synthetic
Source	IDT	IDT	IDT	IDT	IDT	IDT	IDT	IDT	IDT	IDT	IDT	IDT	IDT	IDT
Extinction coefficient (nearest neighbor approximation) (M^{-1} cm^{-1})	202900	241822	211000	244300	253100	233100	208000	206900	201700	272400	190394	202900	297500	200400
vbar (cm^3/g) (estimate)	0.55	0.55	0.55	0.55	0.55	0.55	0.55	0.55	0.55	0.55	0.55	0.55	0.55	0.55
M from chemical composition (Da)	6369	8856	6923	7575	7879	6970	6883	6618	6054	8547	6992	6369	9710	6905
SEC-SAXS column, 10 x 300 Superdex 200														
Loading concentration (mg/ml)	1.7	6.7	3.5	5	2.4	3.5	3.8	3.2	3.4	3.8	6.3	2.1	8.6	3.7
Injection volume (µl)	400	250	300	400	400	300	300	300	300	350	250	400	100	300
Flow rate (ml/min)	0.6	0.6	0.6	0.6	0.6	0.6	0.6	0.6	0.6	0.6	0.6	0.6	0.6	0.6
Solvent (solvent blanks taken from SEC flowthrough prior to elution of protein)	BPEK, 185 mM KCl	BPEK, 185 mM KCl	BPEK, 185 mM KCl	BPEK, 185 mM KCl	BPEK, 185 mM KCl	BPEK, 185 mM KCl	BPEK, 185 mM KCl	BPEK, 185 mM KCl	BPEK, 185 mM KCl	BPEK, 185 mM KCl	BPEK, 185 mM KCl	BPEK, 185 mM KCl	BPEK, 185 mM KCl	BPEK, 185 mM KCl

(b) SAXS data-collection parameters.

Instrument/ data processing	BioCAT facility at the Advanced Photon Source beamline 18ID with Pilatus3 X 1M (Dectris) detector
Wavelength (Å)	1.033
Beam size (µm)	150 (h) x 25 (v)
Camera length (m)	3.655
q measurement range ($Å^{-1}$)	0.0044-0.35
Absolute scaling method	Glassy Carbon, NIST SRM 3600

Normalization	To incident intensity, by ion chamber counter
Monitoring for radiation damage	Automated frame-by-frame comparison of relevant regions
Exposure time, number of exposures	0.5 s exposure time with a 2s total exposure period (0.5 s on, 1.5 s off) of entire SEC elution
Sample configuration	SEC-SAXS. Size separation by an AKTA Pure with a Superdex 200 Increase 10/300 GL column and sheath flow cell(32) to minimize sample damage. SAXS data measured in a 1 mm ID quartz capillary with effective path length 0.542 mm.
Sample temperature (°C)	22

(c) Software employed for SAXS data reduction, analysis, and interpretation.

SAXS data reduction	Radial averaging; frame comparison, averaging, and subtraction done using BioXTAS RAW 1.6.3 (Hopkins et al. 2017(33))
Extinction coefficient estimate	Nearest neighbor approximation
Basic analyses: Guinier, P(r), Vp	Guinier fit, P(r), Kratky analysis, and molecular weight using BioXTAS RAW 2.1.1 and GNOM(33,34)
Shape/bead modelling	N/A
Atomic structure modelling	CRYSOL v2.8.3 (39)
Three-dimensional graphic model representations	UCSF Chimera v1.12

(d) Structural parameters.

	2KZD	201D	6GH0	2GKU	2JSL	5I2V	6L92	2KQG	2LBY	6NEB	1XAV	2KZE	5CMX	2M27
**Guinier analysis**														
I(0) (cm^{-1})	0.92 ± 0.000987	0.01 ± 0.0000118	0.23 ± 0.0015	0.01 ± 0.0000188	0.02 ± 0.0000153	0.37 ± 0.00041	0.4 ± 0.000435	0.22 ± 0.00267	0.27 ± 0.000493	0.52 ± 0.000446	0.00889 ± 0.0000139	0.0068 ± 0.000013	0.00729 ± 0.000012	0.42 ± 0.000616
Rg (Å)	11.7 ± 0.03	12.89 ± 0.03	10.4 ± 0.17	12.27 ± 0.04	12.13 ± 0.03	13.49 ± 0.03	11.76 ± 0.03	15.64 ± 0.44	12.15 ± 0.05	13.56 ± 0.02	12.63 ± 0.04	12.5 ± 0.05	15.44 ± 0.06	12.66 ± 0.04
qmin*Rg	0.112	0.057	0.16	0.072	0.088	0.107	0.052	0.128	0.1	0.061	0.056	0.141	0.127	0.093
qmax*Rg	1.259	1.144	1.287	1.258	1.146	1.276	1.268	1.214	1.268	1.275	1.25	1.267	1.268	1.245
Coefficient of correlation, R^2	0.991	0.991	0.843	0.98	0.988	0.987	0.978	0.144	0.96	0.985	0.971	0.971	0.991	0.981

M (Da) from Vc	5800	7400	8400	6500	6700	10700	8900	9400	9000	8100	6800	6300	12800	10300
**P(r) analysis (GNOM)**														
I(0) (cm^{-1})	0.93 ± 0.000949	0.01 ± 0.0000147	0.24 ± 0.000568	0.01 ± 0.0000177	0.02 ± 0.0000185	0.37 ± 0.000452	0.4 ± 000486	0.2 ± 00166	0.27 ± 0.000502	0.52 ± 0.000439	0.00887 ± 0.0000143	0.00679 ± 0.0000127	0.00731 ± 0.0000112	0.42 ± 0.000596
Rg (Å)	11.73 ± 0.02	12.88 ± 0.03	11.24 ± 0.03	12.23 ± 0.03	12.2 ± 0.03	13.61 ± 0.04	11.81 ± 0.03	11.88 ± 0.1	12.19 ± 0.03	13.64 ± 0.02	12.56 ± 0.03	12.49 ± 0.04	15.68 ± 0.04	12.64 ± 0.03
Dmax (Å)	36	41	31	38	39	49	39	32	39	45	41	42	52	38
X^2	1.386	0.99	3.032	1.201	1.354	2.237	1.961	3.185	2.098	1.74	1.28	1.355	1.032	1.439
Porod volume ($Å^{-3}$)	3460	5950	2260	4500	4670	6030	3440	3100	3740	7000	4940	4320	8980	5260
**Ambimeter**														
Number of compatible shape categories, (Ambiguity score)	31 (1.49)	5 (0.7)	174 (2.24)	32 (1.5)	8 (0.9)	19 (1.28)	2 (0.3)	33 (1.52)	113 (2.05)	11 (1.04)	20 (1.3)	69 (1.84)	132 (2.12)	12 (1.08)
3D reconstruction	Potentially unique	Potentially unique	might be ambiguous	might be ambiguous	potentially unique	potentially unique	potentially unique	might be ambiguous	might be ambiguous	potentially unique	potentially unique	might be ambiguous	might be ambiguous	potentially unique
(f) Atomistic modelling.														
Model (PDB ID)	2KZD	201D	6GH0	2GKU	2JSL	5I2V	6L92	2KQG	2LBY	6NEB	1XAV	2KZE	5CMX	2M27
q range	0.0096 - 0.3497	0.0044 - 0.3497	0.0154 - 0.35	0.0059 - 0.3497	0.0073 - 0.3497	0.0079 - 0.35	0.0045 - 0.35	0.0082 - 0.35	0.0082 - 0.35	0.0045 - 0.35	0.0044 - 0.3497	0.0113 - 0.3497	0.0082 - 0.35	0.0073 - 0.35
**CRYSOL (constant subtraction allowed)**														
X^2	2.261	1.59	3.185	1.166	1.413	2.943	2.317	3.215	2.038	3.103	1.44	1.428	1.640	1.642
Calculated Rg (Å)	11.48	12.68	11.27	12.03	12.18	12.98	11.57	12.04	12.11	13.15	12.31	12.47	14.80	12.47
Dro (e $Å^{-3}$), Ra (Å)	0.075, 1.52	0.065, 1.80	0.018, 1.80	0.072, 1.80	0.062, 1.80	0.075, 1.80	0.075, 1.80	0.070, 1.40	0.043, 1.64	0.075, 1.40	0.075, 1.80	0.075, 1.80	0.075, 1.40	0.075, 1.80
(g) SASBDB IDs for data and models.														
**ID**	SASDM56	SASDM76	SASDM86	SASDM96	SASDKF3	SASDMA6	SASDMB6	SASDMC6	SASDMD6	SASDME6	SASDMF6	SASDM66	SASDMG6	SASDMH6

Table S4. Tabulated small-angle X-ray scattering data acquisition, reduction, analysis, results and SASBDB identifiers of oligonucleotides from the PDB used in CRYSOL R_g regression.

	c-Myc-8			c-Myc-12			c-Myc-12 Post-DNaseI		
**MW (Da)**	10980			22250			20600		
**Calculated volume (Å³) (eq. 2 & 3)**	15842			32102			29721		
**Avg vol per atom (from DAMMIN refine header)**	11.06			152.80			30.34		
**Calculated AER (eq. 4)**	1.38			3.32			1.93		
**Adjusted AER**	1.18			2.60			1.49		
	**HYDROPRO**	**SAXS**	**AUC**	**HYDROPRO**	**SAXS**	**AUC**	**HYDROPRO**	**SAXS**	**AUC**
**Radius of gyration (Å)**	14.20	14.43		23.70	23.7		20.60	20.33	
**Volume (Å³)**	17000	9500		33800	27400		29100	19600	
**Sedimentation coefficient (S)**	2.53		2.60	3.04		3.57	3.26		3.61

	c-Kit-8			c-Kit-12			c-Kit-12 DNaseI			k-Ras-8		
**MW (Da)**	12140			20340			14700			13750		
**Calculated volume (Å³) (eq. 2 & 3)**	17516			29347			21209			19839		
**V_a (from DAMMIN refine header)**	11.06			45.28			19.10			30.34		
**Calculated AER (eq. 4)**	1.38			2.21			1.66			1.93		
**Adjusted AER**	1.20			1.77			1.38			1.70		
	**HYDROPRO**	**SAXS**	**AUC**	**HYDROPRO**	**SAXS**	**AUC**	**HYDROPRO**	**SAXS**	**AUC**	**HYDROPRO**	**SAXS**	**AUC**
**Radius of gyration (Å)**	15.00	14.95		26.50	25.1		16.40	16.55		17.40	16.98	
**Volume (Å³)**	18800	11400		30100	23900		20800	12700		18500	14600	
**Sedimentation coefficient (S)**	2.46		2.70	2.96		3.33	2.82		2.85	2.56		2.83

Table S5. Tabulated hydrodynamic results from SAXS-derived *ab initio* bead models.

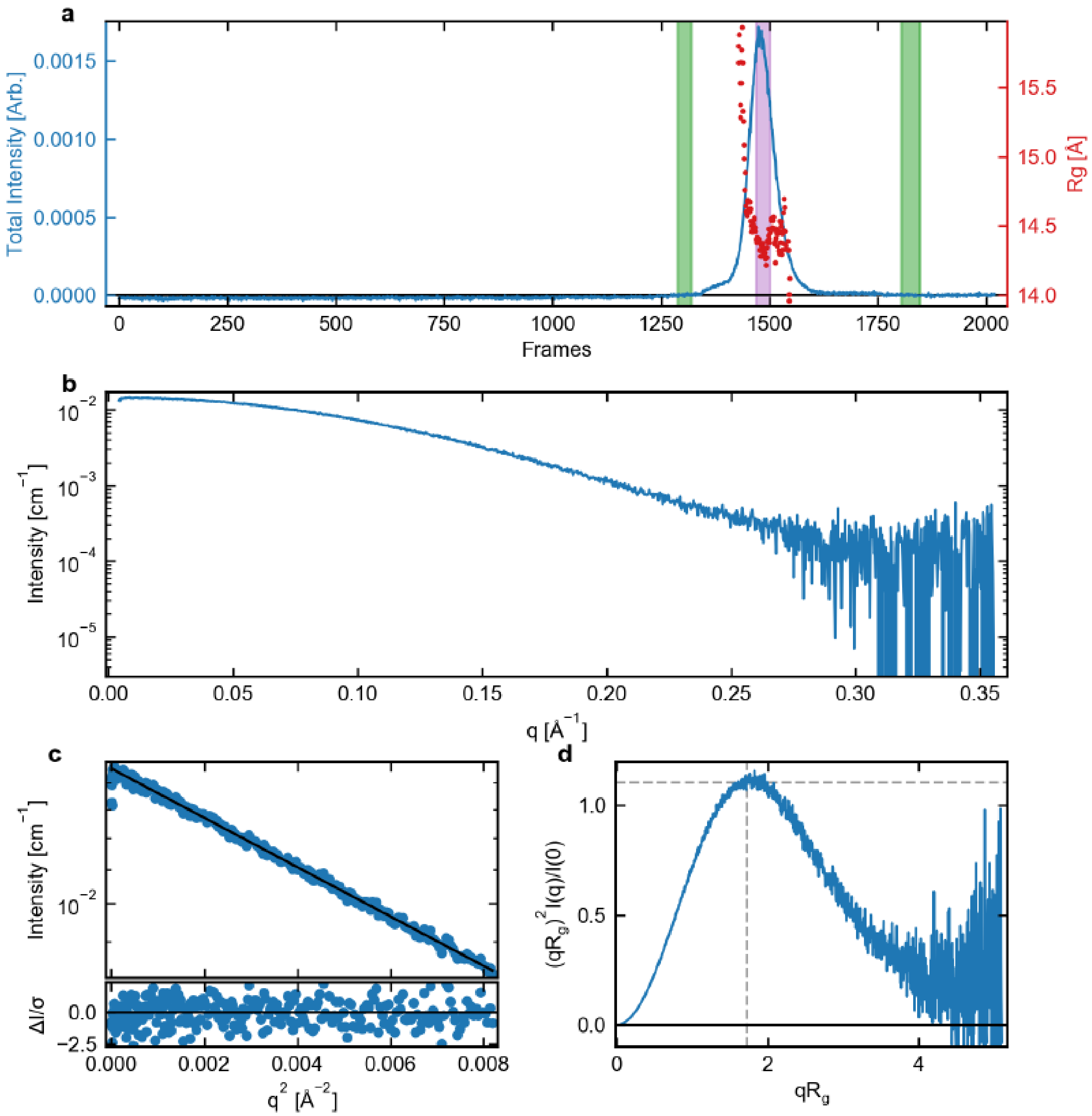

S1. SAXS data summary for c-Myc-8. a) Series intensity (blue, left axis) vs. frame, and, if available, Rg vs. frame (red, right axis). Green shaded regions are buffer regions, purple shaded regions are sample regions. b) Scattering profile(s) on a log-lin scale. c) Guinier fit(s) (top) and fit residuals (bottom). d) Normalized Kratky plot. Dashed lines show where a globular system would peak. Figure created in BioXTAS RAW v2.1.1.

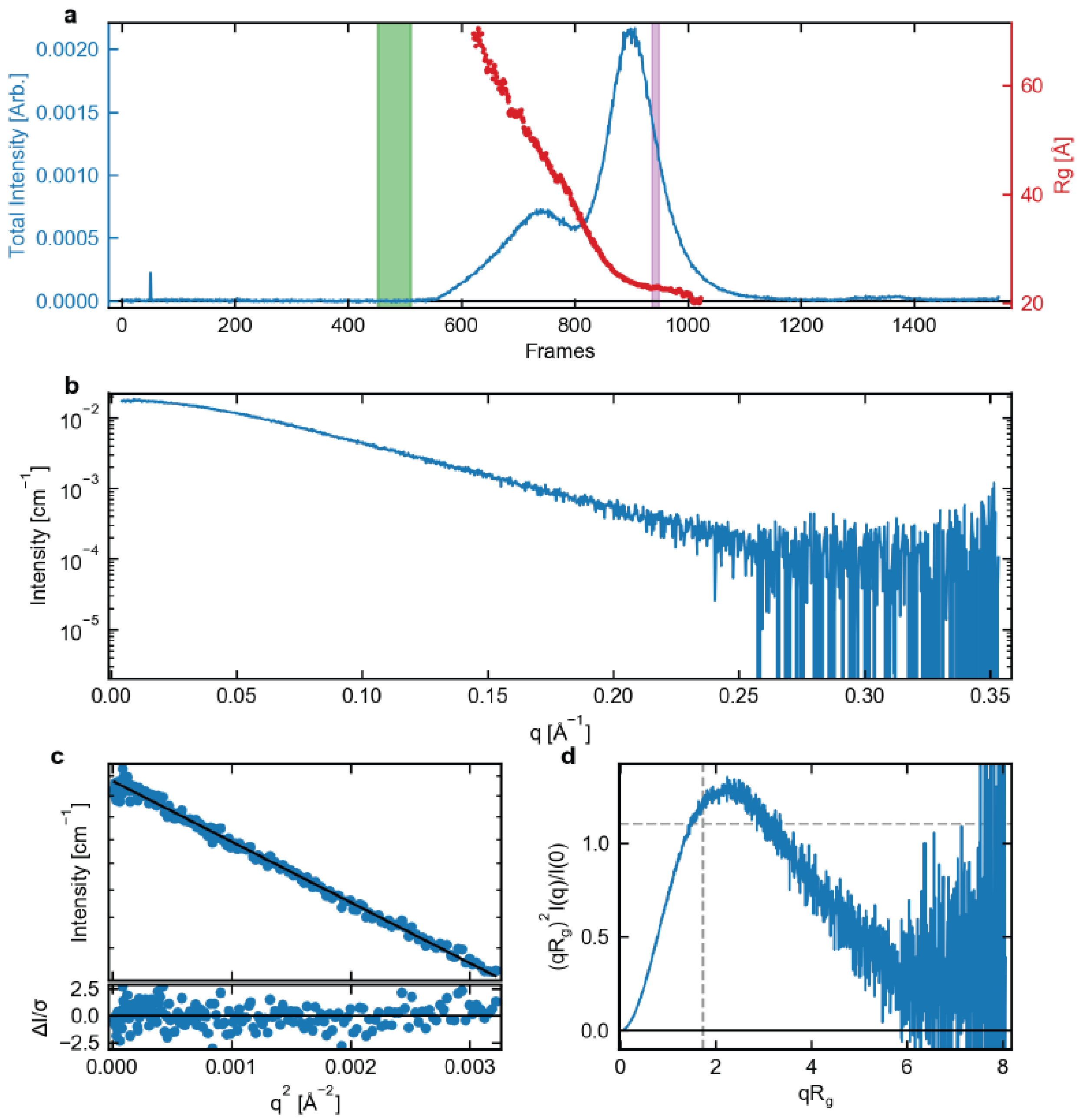

S2. SAXS data summary for c-Myc-12. a) Series intensity (blue, left axis) vs. frame, and, if available, Rg vs. frame (red, right axis). Green shaded regions are buffer regions, purple shaded regions are sample regions. b) Scattering profile(s) on a log-lin scale. c) Guinier fit(s) (top) and fit residuals (bottom). d) Normalized Kratky plot. Dashed lines show where a globular system would peak. Figure created in BioXTAS RAW v2.1.1.

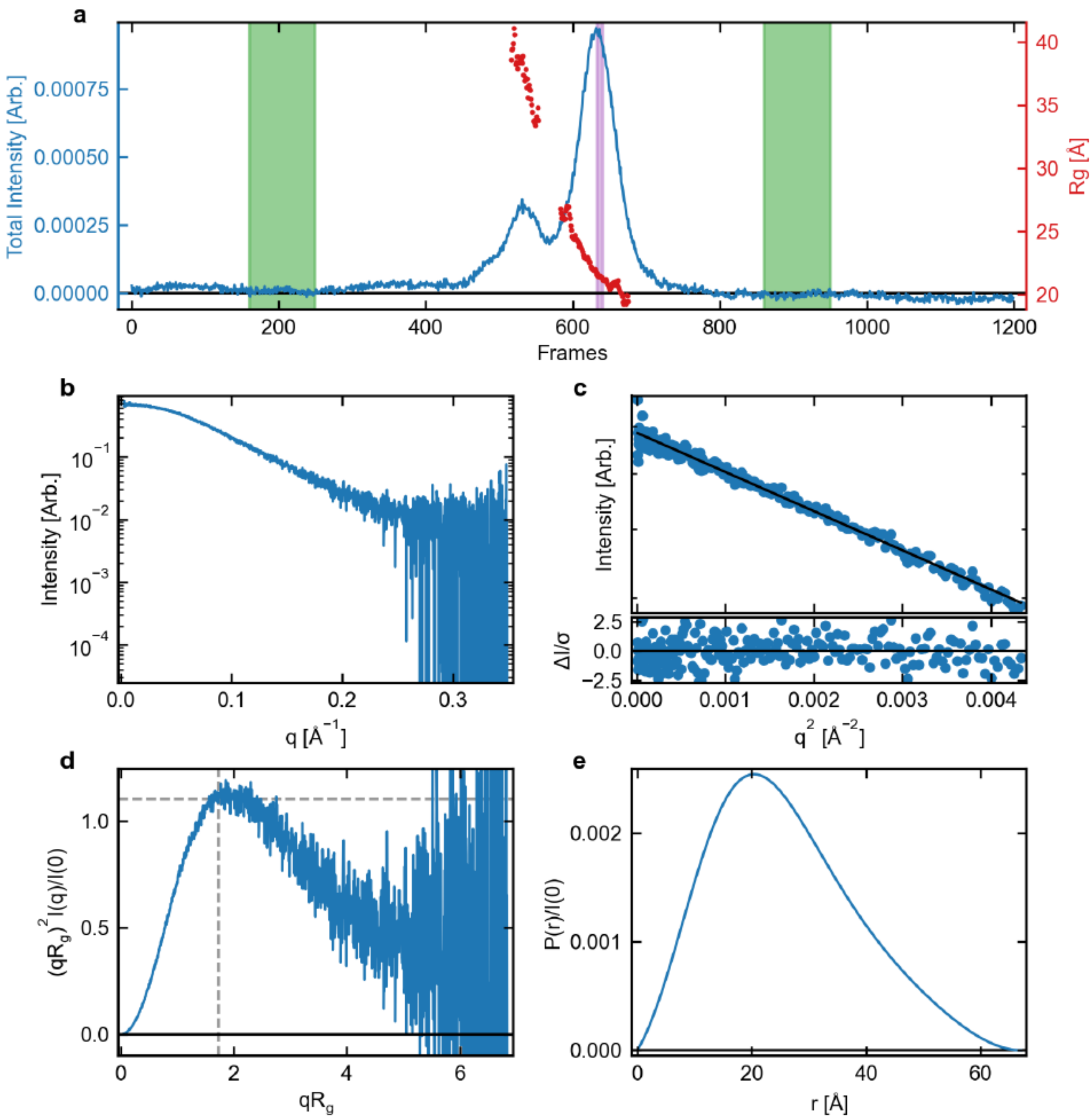

S3. SAXS data summary for c-Myc-12 Post-DNaseI. a) Series intensity (blue, left axis) vs. frame, and, if available, Rg vs. frame (red, right axis). Green shaded regions are buffer regions, purple shaded regions are sample regions. b) Scattering profile(s) on a log-lin scale. c) Guinier fit(s) (top) and fit residuals (bottom). d) Normalized Kratky plot. Dashed lines show where a globular system would peak. e) P(r) function(s), normalized by I(0). Figure created in BioXTAS RAW v2.1.1.

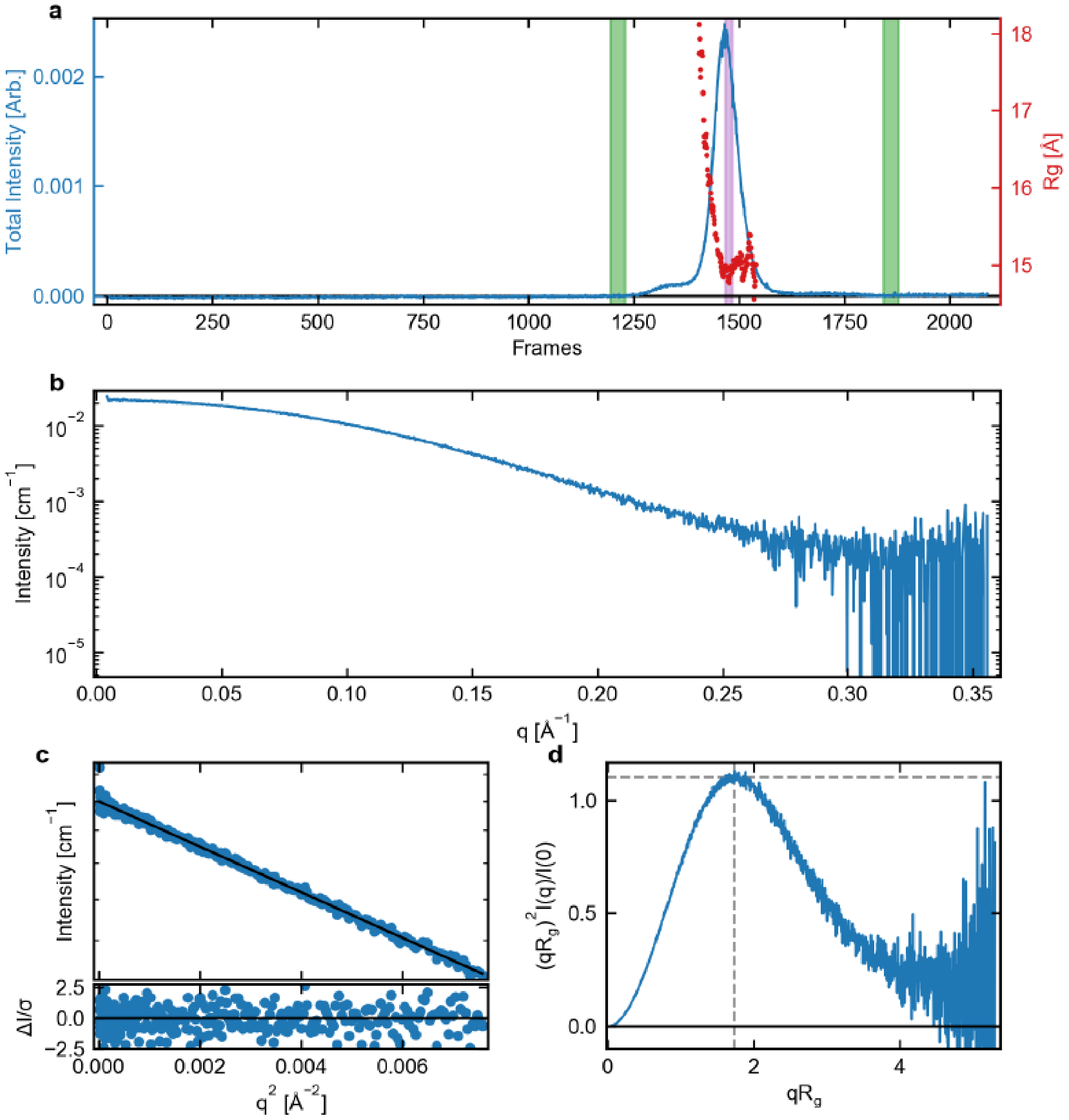

S4. SAXS data summary for c-Kit-8. a) Series intensity (blue, left axis) vs. frame, and, if available, Rg vs. frame (red, right axis). Green shaded regions are buffer regions, purple shaded regions are sample regions. b) Scattering profile(s) on a log-lin scale. c) Guinier fit(s) (top) and fit residuals (bottom). d) Normalized Kratky plot. Dashed lines show where a globular system would peak. Figure created in BioXTAS RAW v2.1.1.

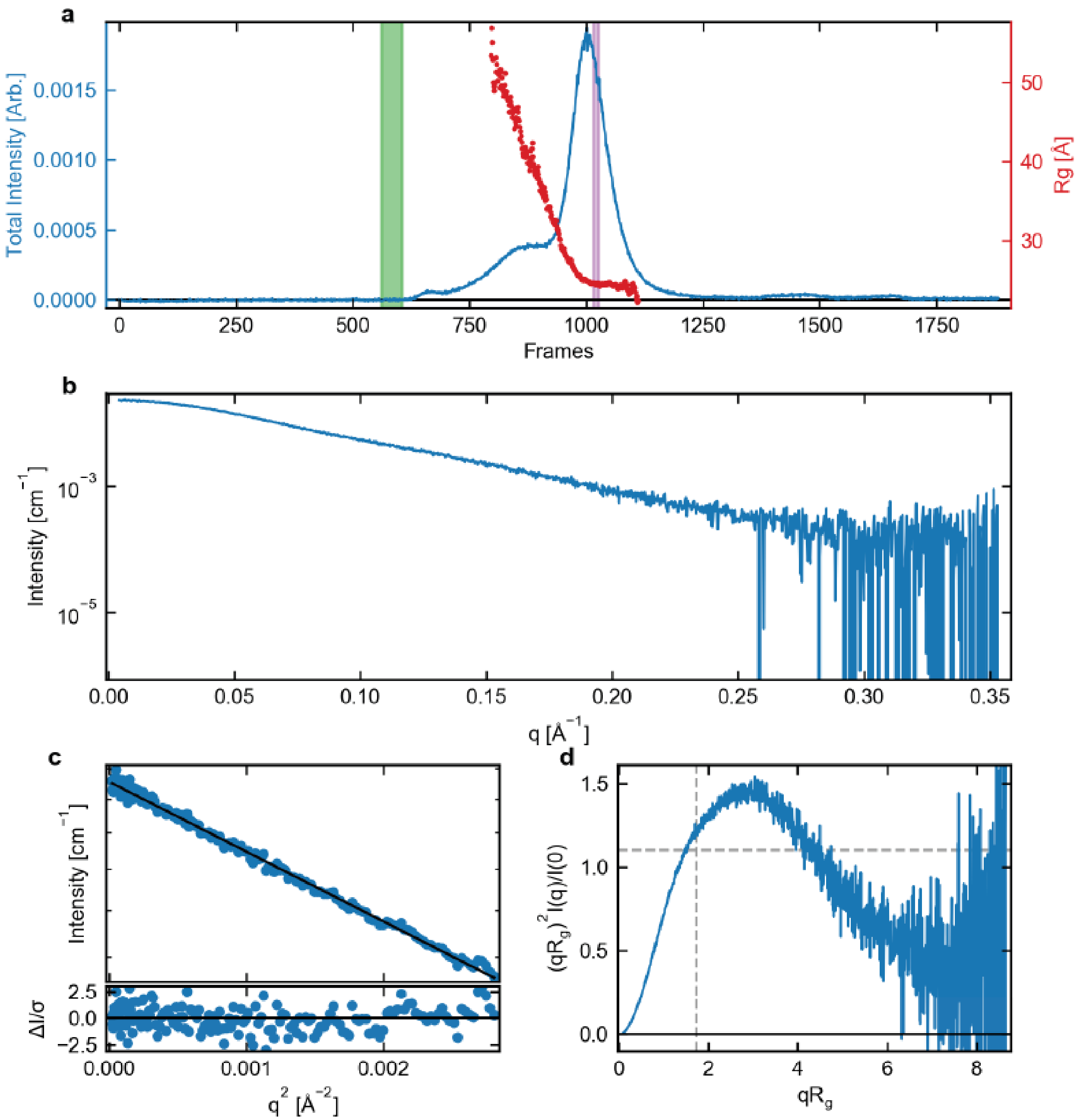

S5. SAXS data summary for c-Kit-12. a) Series intensity (blue, left axis) vs. frame, and, if available, Rg vs. frame (red, right axis). Green shaded regions are buffer regions, purple shaded regions are sample regions. b) Scattering profile(s) on a log-lin scale. c) Guinier fit(s) (top) and fit residuals (bottom). d) Normalized Kratky plot. Dashed lines show where a globular system would peak. Figure created in BioXTAS RAW v2.1.1.

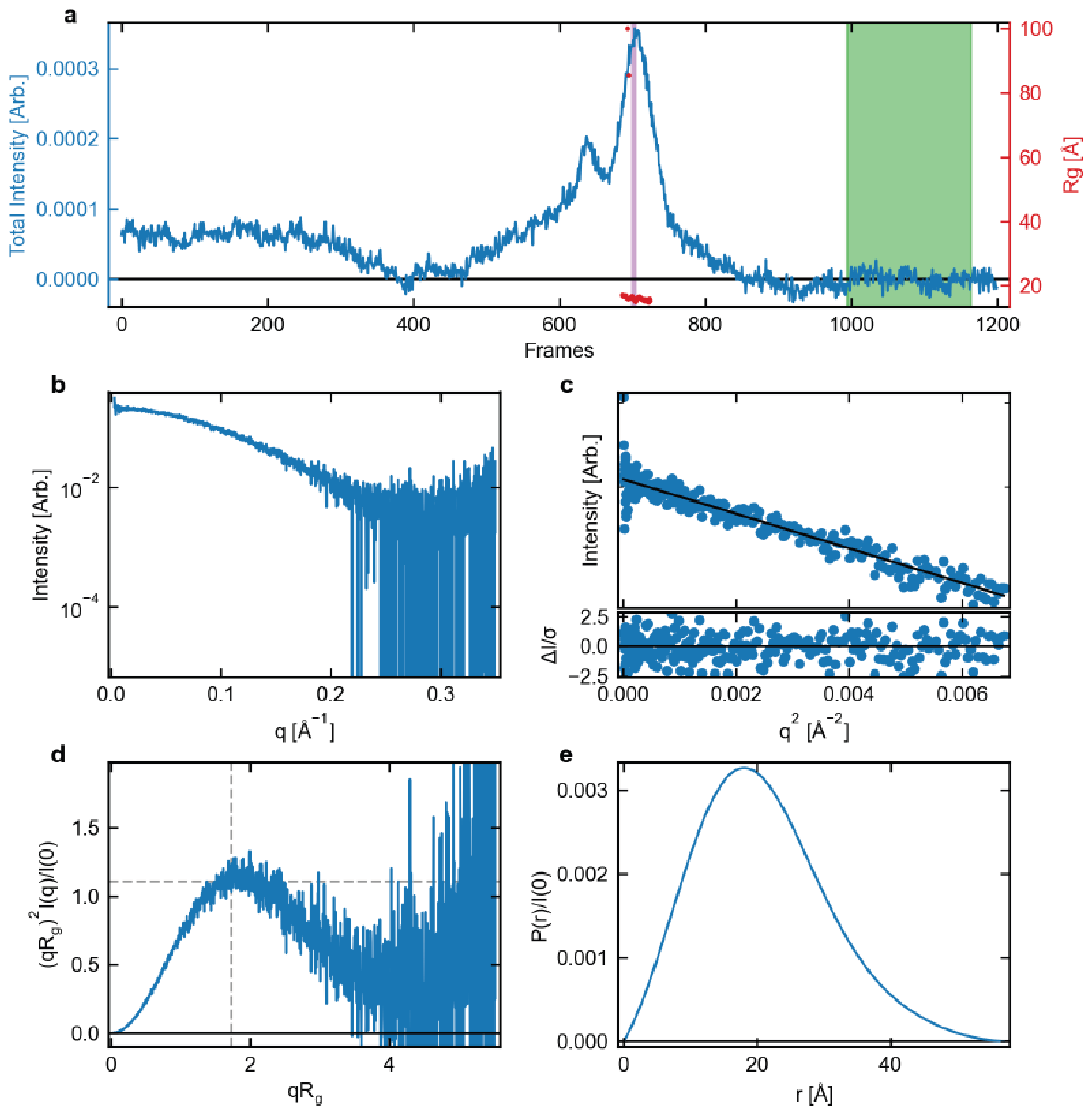

S6. SAXS data summary for c-Kit-12 Post-DNaseI. a) Series intensity (blue, left axis) vs. frame, and, if available, Rg vs. frame (red, right axis). Green shaded regions are buffer regions, purple shaded regions are sample regions. b) Scattering profile(s) on a log-lin scale. c) Guinier fit(s) (top) and fit residuals (bottom). d) Normalized Kratky plot. e) Normalized Pr distribution. Dashed lines show where a globular system would peak. Figure created in BioXTAS RAW v2.1.1.

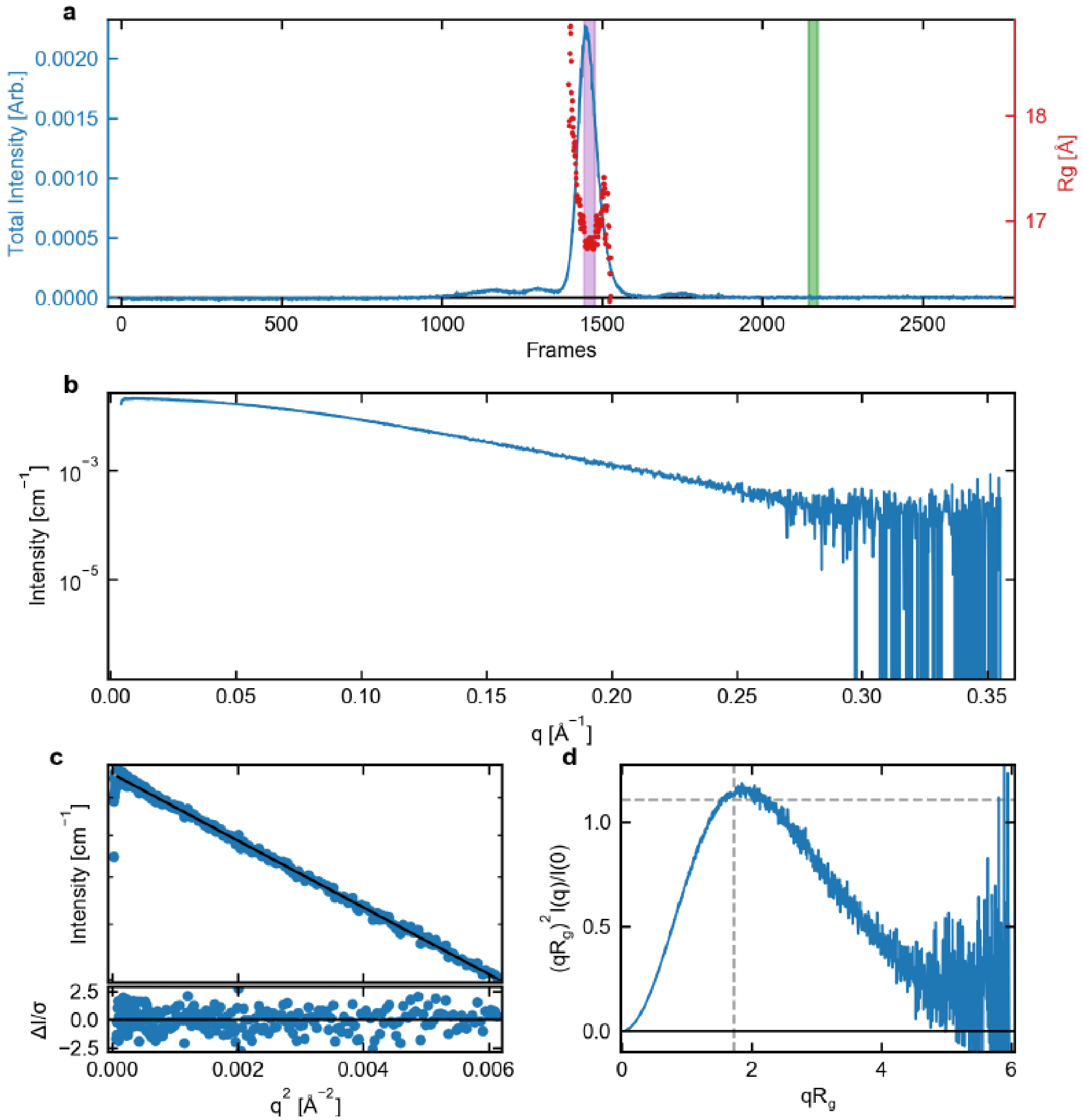

S7. SAXS data summary for k-Ras-8 Post-DNaseI. a) Series intensity (blue, left axis) vs. frame, and, if available, Rg vs. frame (red, right axis). Green shaded regions are buffer regions, purple shaded regions are sample regions. b) Scattering profile(s) on a log-lin scale. c) Guinier fit(s) (top) and fit residuals (bottom). d) Normalized Kratky plot. Dashed lines show where a globular system would peak. Figure created in BioXTAS RAW v2.1.1.

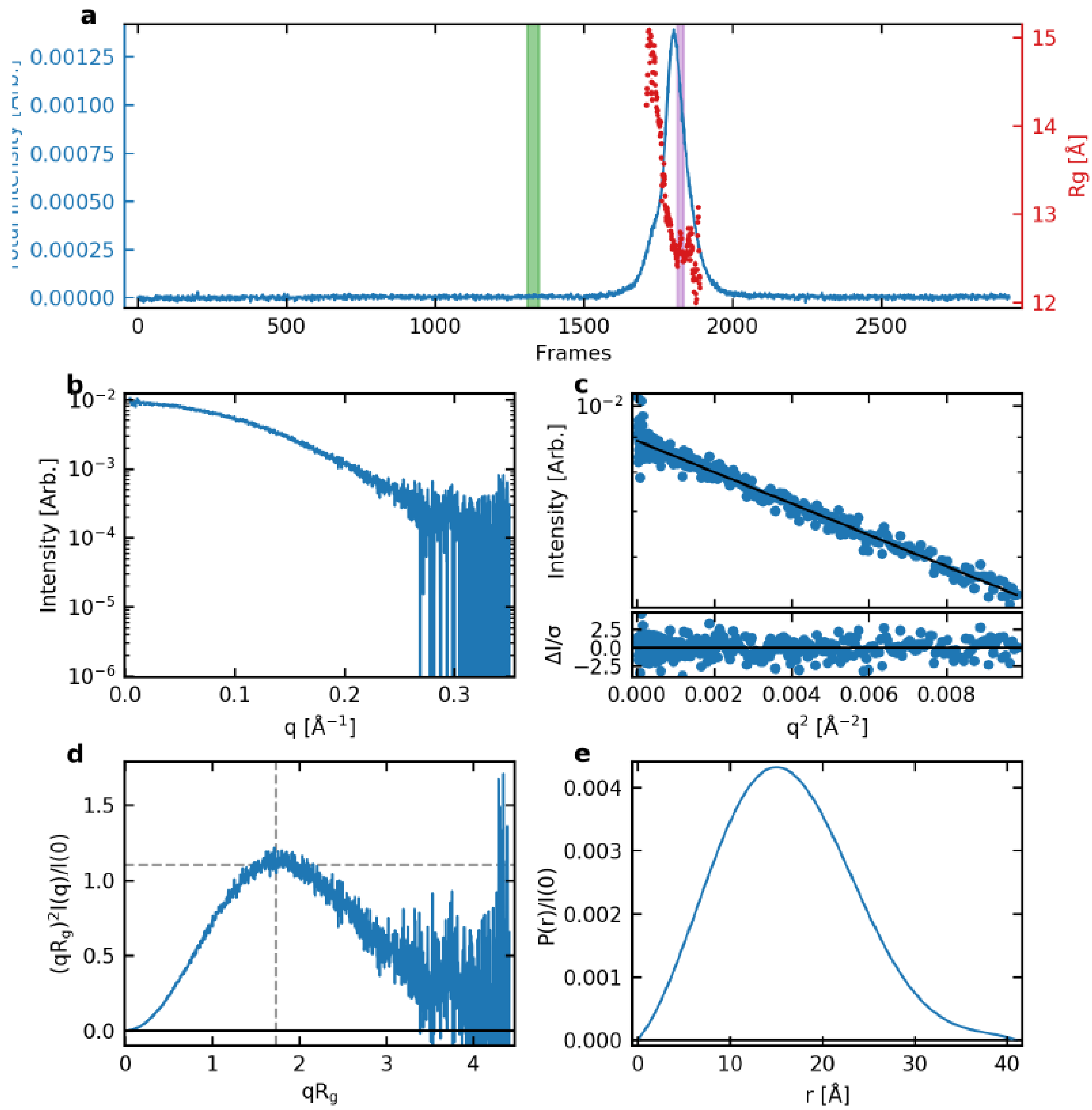

S8. SAXS data summary for 1XAV. a) Series intensity (blue, left axis) vs. frame, and, if available, Rg vs. frame (red, right axis). Green shaded regions are buffer regions, purple shaded regions are sample regions. b) Scattering profile(s) on a log-lin scale. c) Guinier fit(s) (top) and fit residuals (bottom). d) Normalized Kratky plot. Dashed lines show where a globular system would peak. e) P(r) function(s), normalized by I(0). Figure created in BioXTAS RAW v2.1.1.

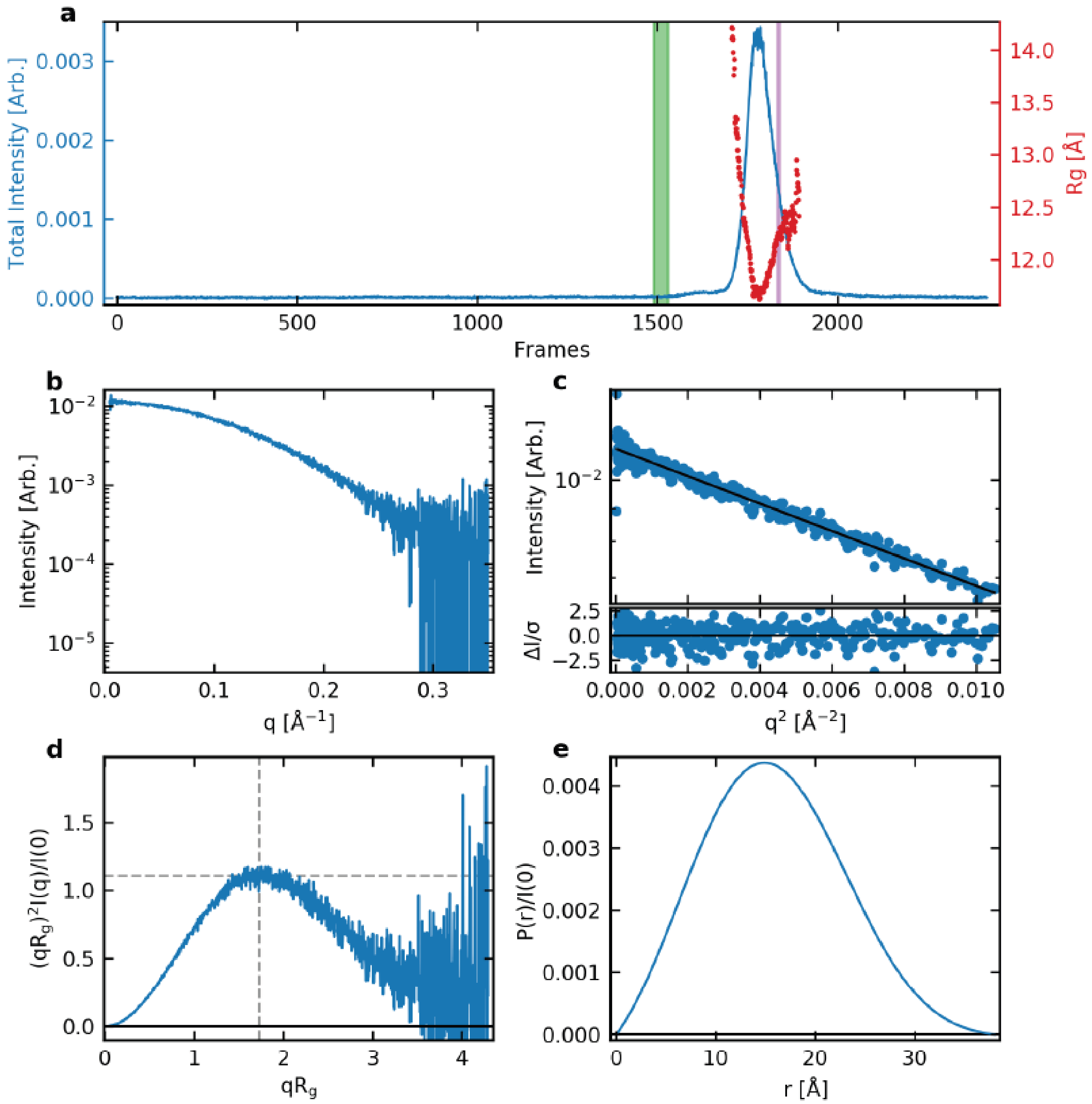

S9. SAXS data summary for 2GKU. a) Series intensity (blue, left axis) vs. frame, and, if available, Rg vs. frame (red, right axis). Green shaded regions are buffer regions, purple shaded regions are sample regions. b) Scattering profile(s) on a log-lin scale. c) Guinier fit(s) (top) and fit residuals (bottom). d) Normalized Kratky plot. Dashed lines show where a globular system would peak. e) P(r) function(s), normalized by I(0). Figure created in BioXTAS RAW v2.1.1.

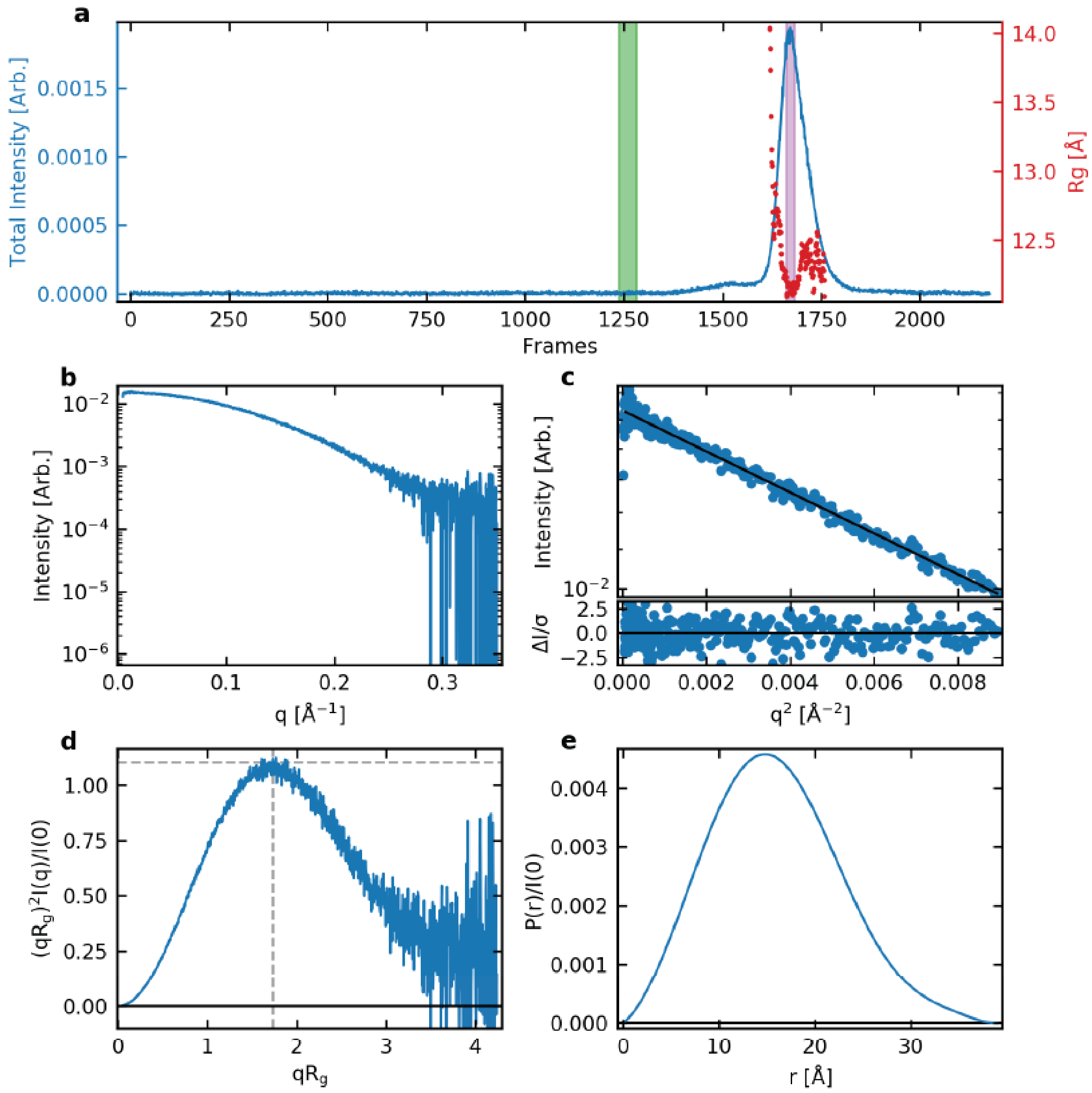

S10. SAXS data summary for 2JSL. a) Series intensity (blue, left axis) vs. frame, and, if available, Rg vs. frame (red, right axis). Green shaded regions are buffer regions, purple shaded regions are sample regions. b) Scattering profile(s) on a log-lin scale. c) Guinier fit(s) (top) and fit residuals (bottom). d) Normalized Kratky plot. Dashed lines show where a globular system would peak. e) P(r) function(s), normalized by I(0). Figure created in BioXTAS RAW v2.1.1.

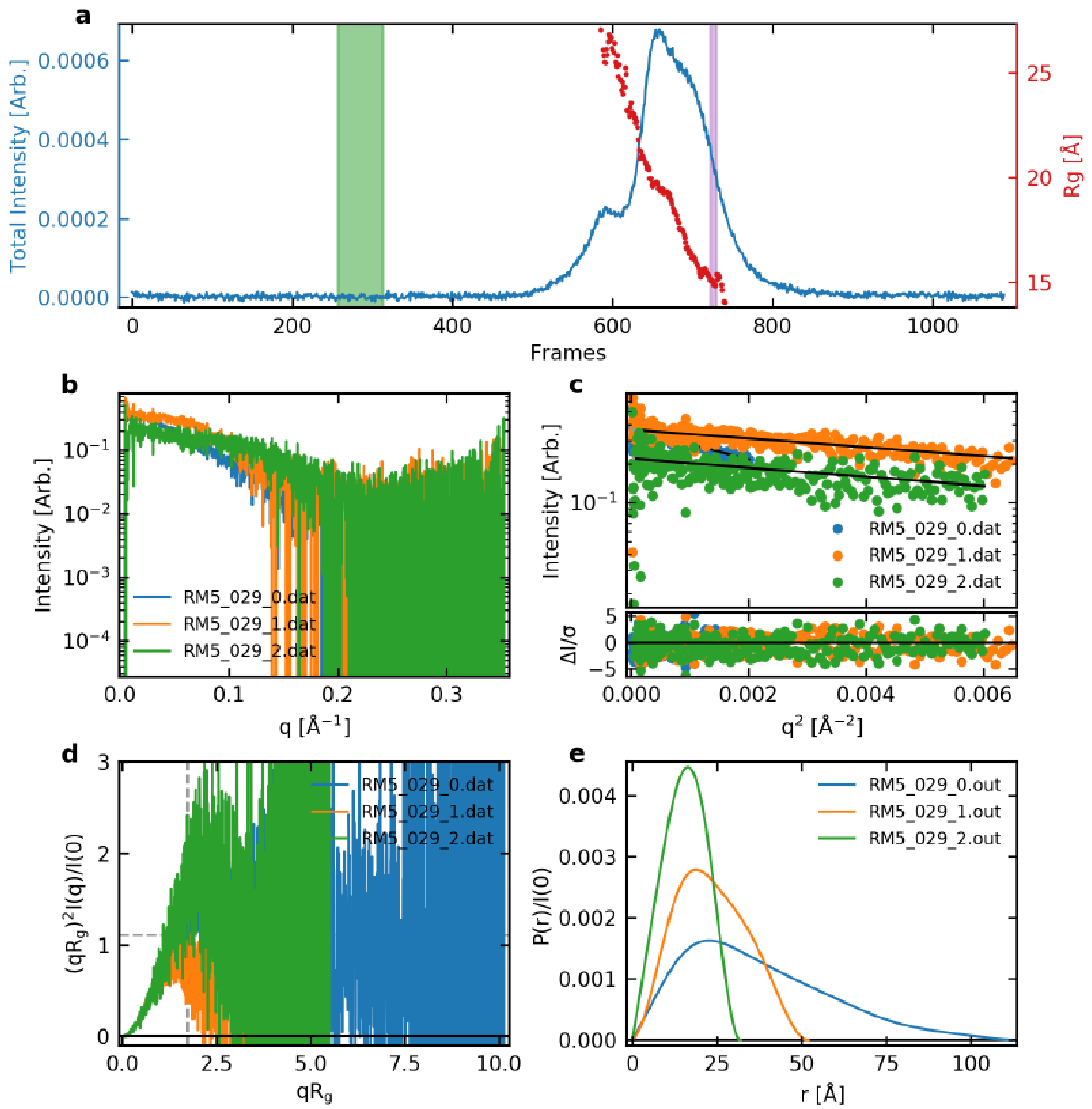

S11. SAXS data summary for 2KQG. a) Series intensity (blue, left axis) vs. frame, and, if available, Rg vs. frame (red, right axis). Green shaded regions are buffer regions, purple shaded regions are sample regions. b) Scattering profile(s) on a log-lin scale. c) Guinier fit(s) (top) and fit residuals (bottom). d) Normalized Kratky plot. Dashed lines show where a globular system would peak. e) P(r) function(s), normalized by I(0). Green (029_2), orange (029_1), and blue (029_0) species are the result of deconvolution using evolving factor analysis(1). The monomeric species, as determined by approximate molecular weight (Vc), is shown in green. Figure created in BioXTAS RAW v2.1.1.

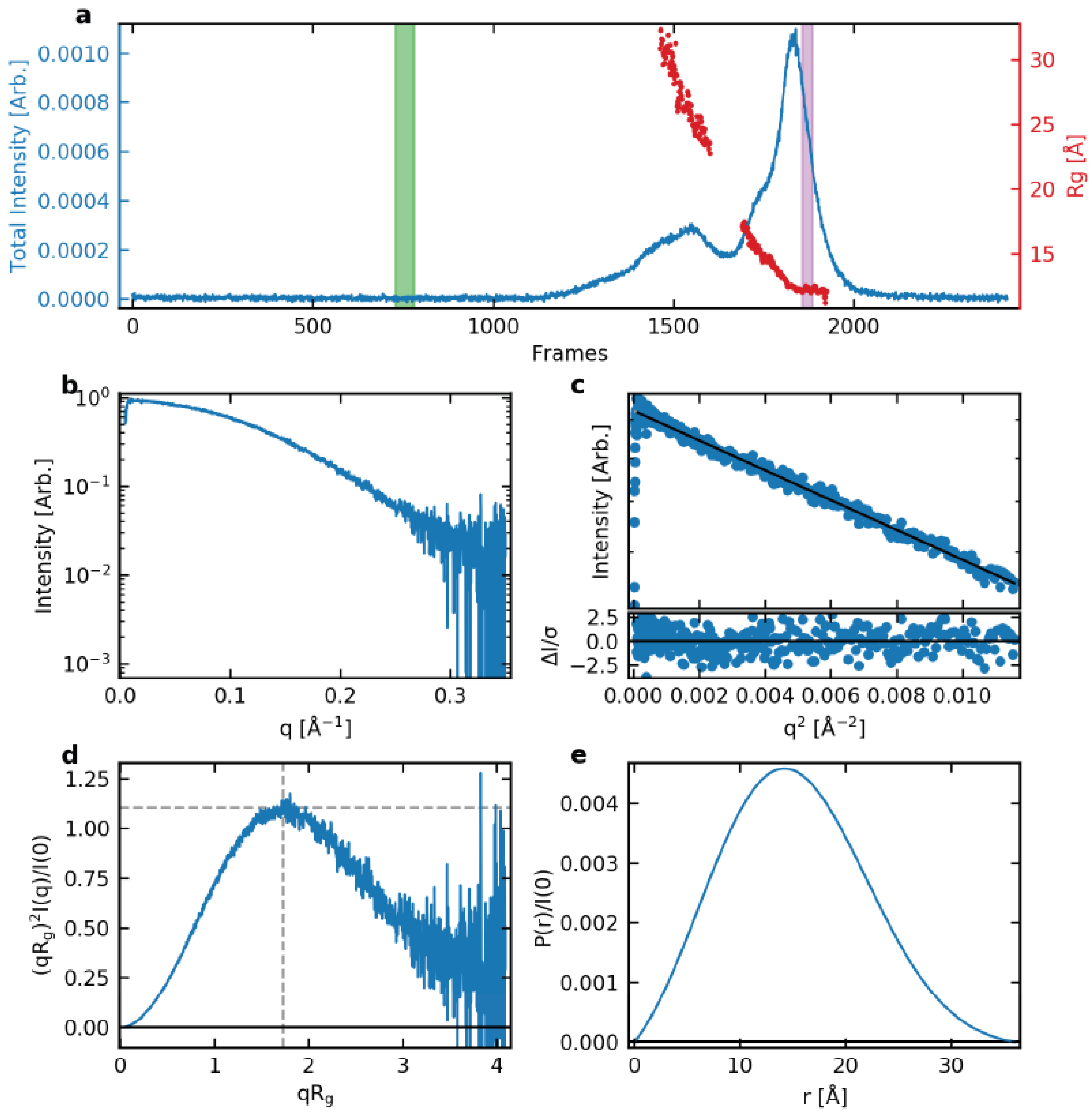

S12. SAXS data summary for 2KZD. a) Series intensity (blue, left axis) vs. frame, and, if available, Rg vs. frame (red, right axis). Green shaded regions are buffer regions, purple shaded regions are sample regions. b) Scattering profile(s) on a log-lin scale. c) Guinier fit(s) (top) and fit residuals (bottom). d) Normalized Kratky plot. Dashed lines show where a globular system would peak. e) P(r) function(s), normalized by I(0). Figure created in BioXTAS RAW v2.1.1.

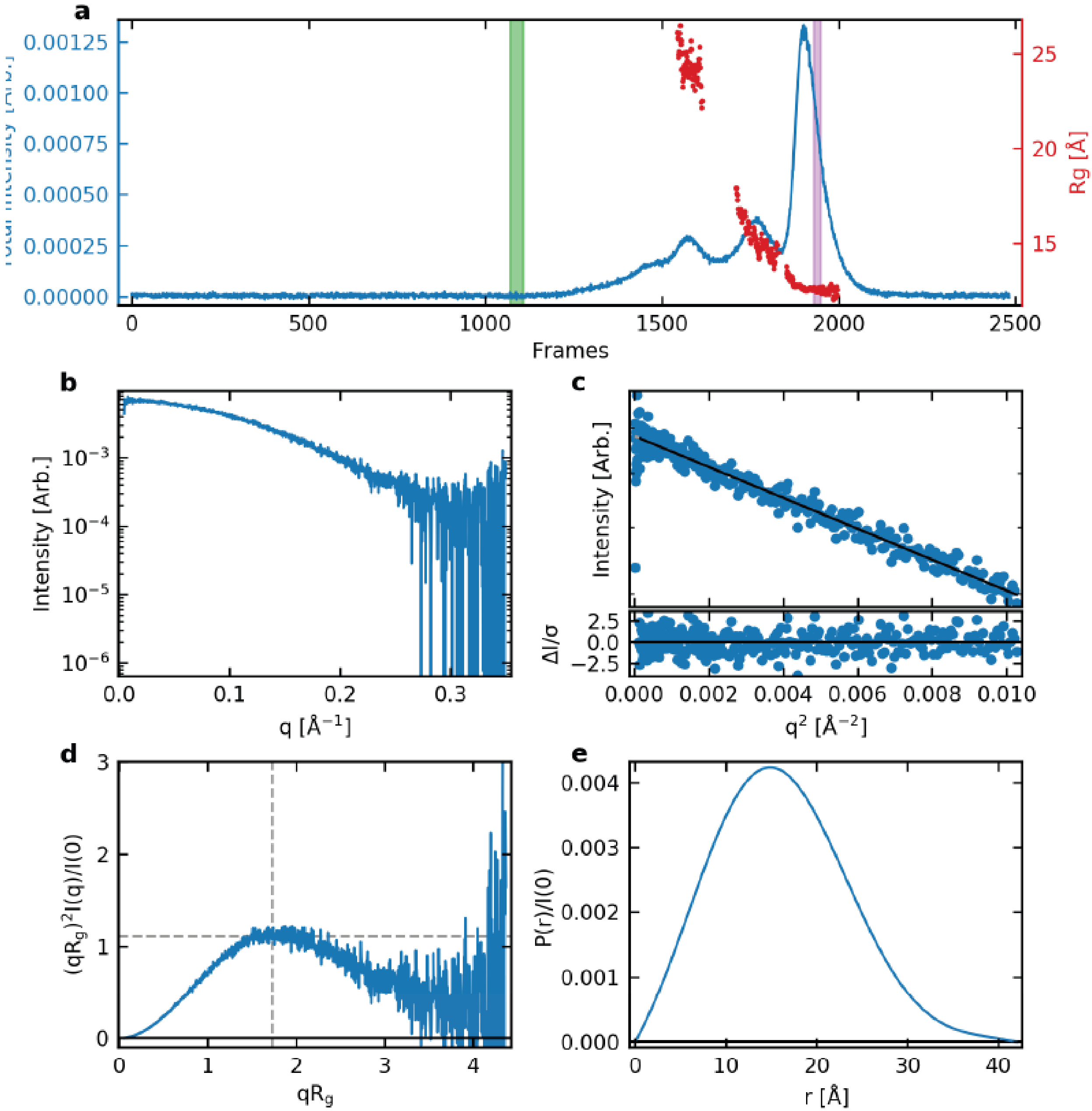

S13. SAXS data summary for 2KZE. a) Series intensity (blue, left axis) vs. frame, and, if available, Rg vs. frame (red, right axis). Green shaded regions are buffer regions, purple shaded regions are sample regions. b) Scattering profile(s) on a log-lin scale. c) Guinier fit(s) (top) and fit residuals (bottom). d) Normalized Kratky plot. Dashed lines show where a globular system would peak. e) P(r) function(s), normalized by I(0). Figure created in BioXTAS RAW v2.1.1.

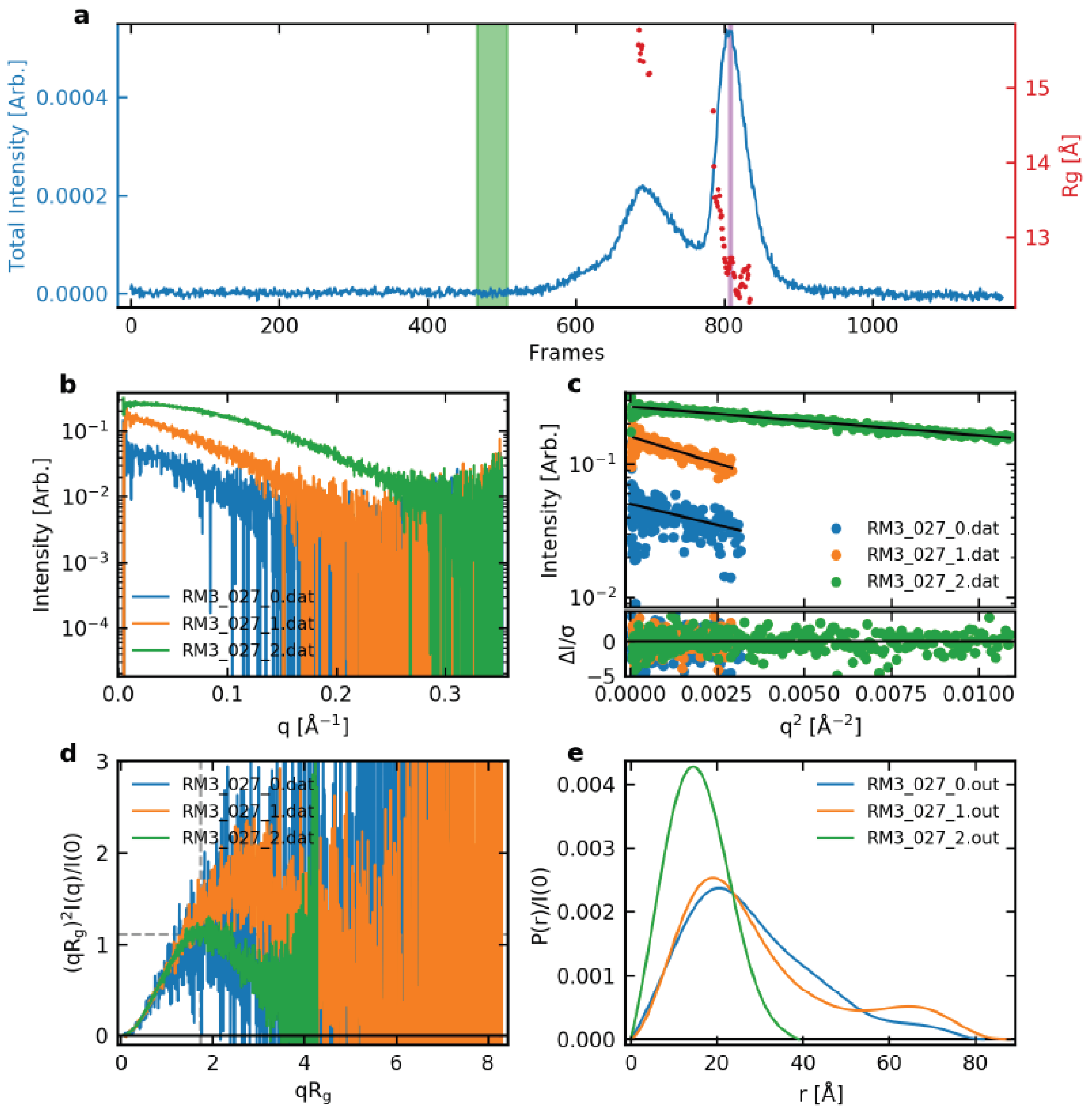

S14. SAXS data summary for 2LBY. a) Series intensity (blue, left axis) vs. frame, and, if available, Rg vs. frame (red, right axis). Green shaded regions are buffer regions, purple shaded regions are sample regions. b) Scattering profile(s) on a log-lin scale. c) Guinier fit(s) (top) and fit residuals (bottom). d) Normalized Kratky plot. Dashed lines show where a globular system would peak. e) P(r) function(s), normalized by I(0). Green (027_2), orange (027_1), and blue (027_0) species are the result of deconvolution using evolving factor analysis(1). The monomeric species, as determined by approximate molecular weight (Vc), is shown in green. Figure created in BioXTAS RAW v2.1.1.

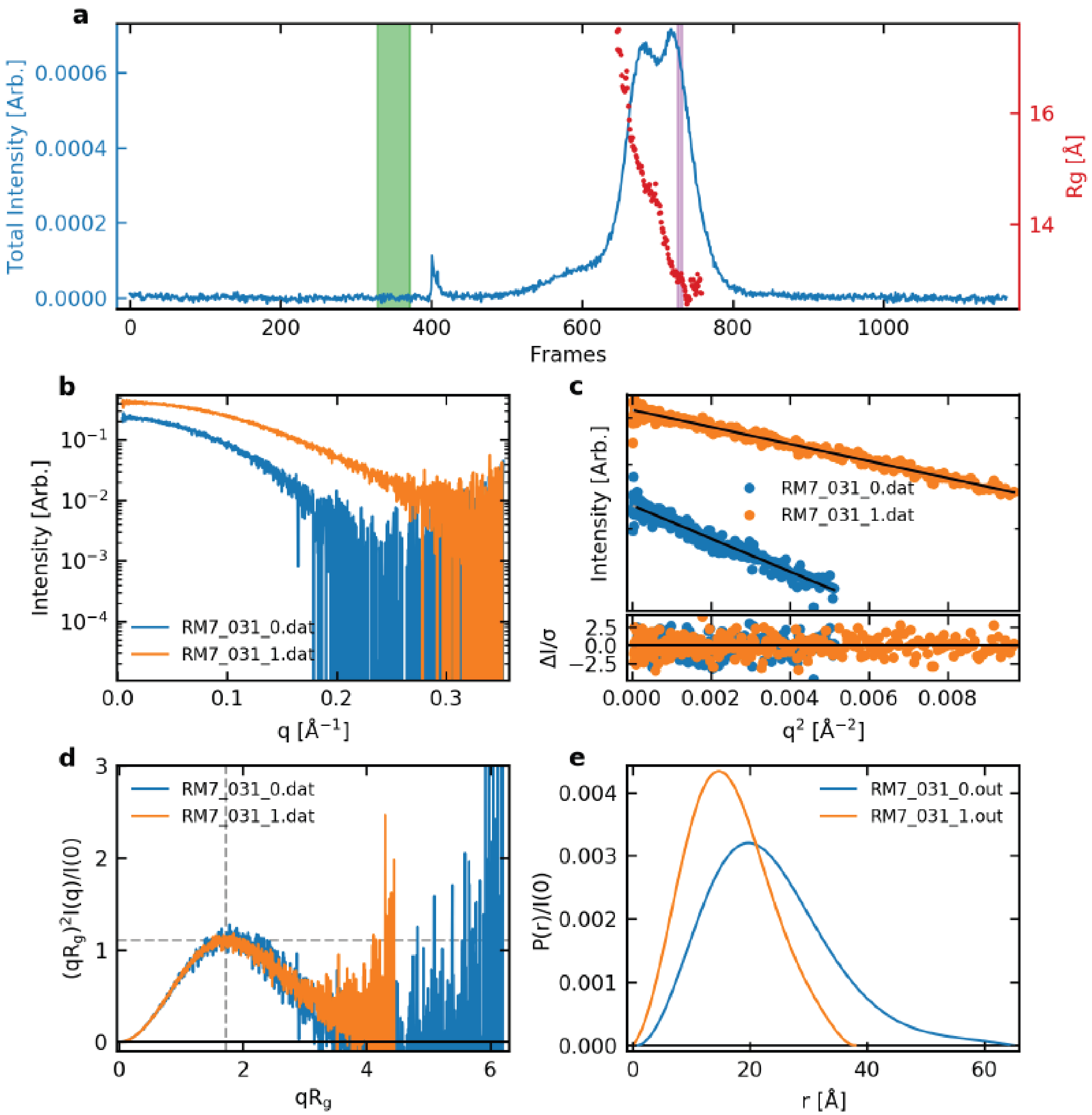

S15. SAXS data summary for 2M27. a) Series intensity (blue, left axis) vs. frame, and, if available, Rg vs. frame (red, right axis). Green shaded regions are buffer regions, purple shaded regions are sample regions. b) Scattering profile(s) on a log-lin scale. c) Guinier fit(s) (top) and fit residuals (bottom). d) Normalized Kratky plot. Dashed lines show where a globular system would peak. e) P(r) function(s), normalized by I(0). Orange (031_1), and blue (031_0) species are the result of deconvolution using evolving factor analysis(1). The monomeric species, as determined by approximate molecular weight (Vc), is shown in orange. Figure created in BioXTAS RAW v2.1.1.

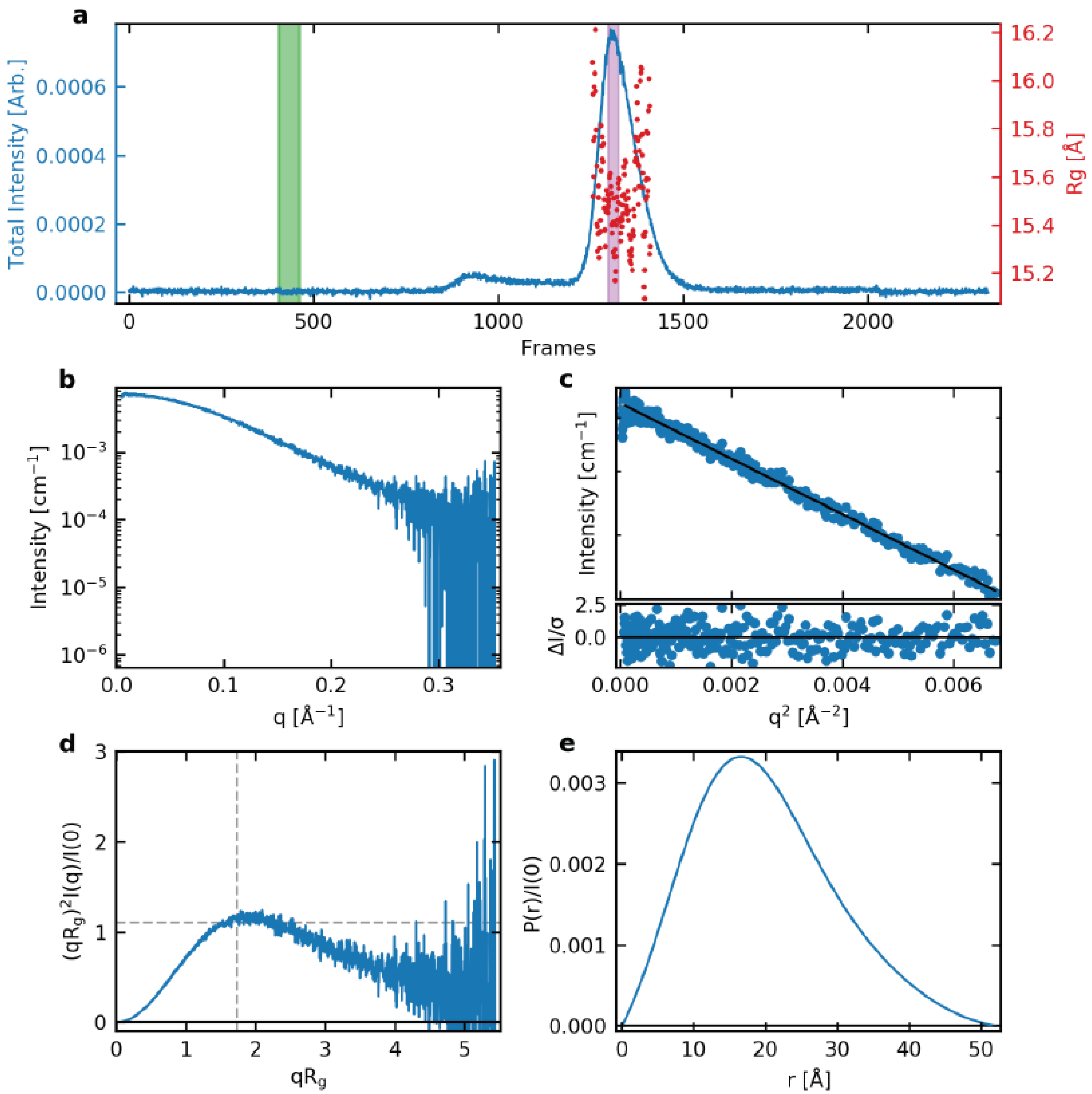

S16. SAXS data summary for 5CMX. a) Series intensity (blue, left axis) vs. frame, and, if available, Rg vs. frame (red, right axis). Green shaded regions are buffer regions, purple shaded regions are sample regions. b) Scattering profile(s) on a log-lin scale. c) Guinier fit(s) (top) and fit residuals (bottom). d) Normalized Kratky plot. Dashed lines show where a globular system would peak. e) P(r) function(s), normalized by I(0). Figure created in BioXTAS RAW v2.1.1.

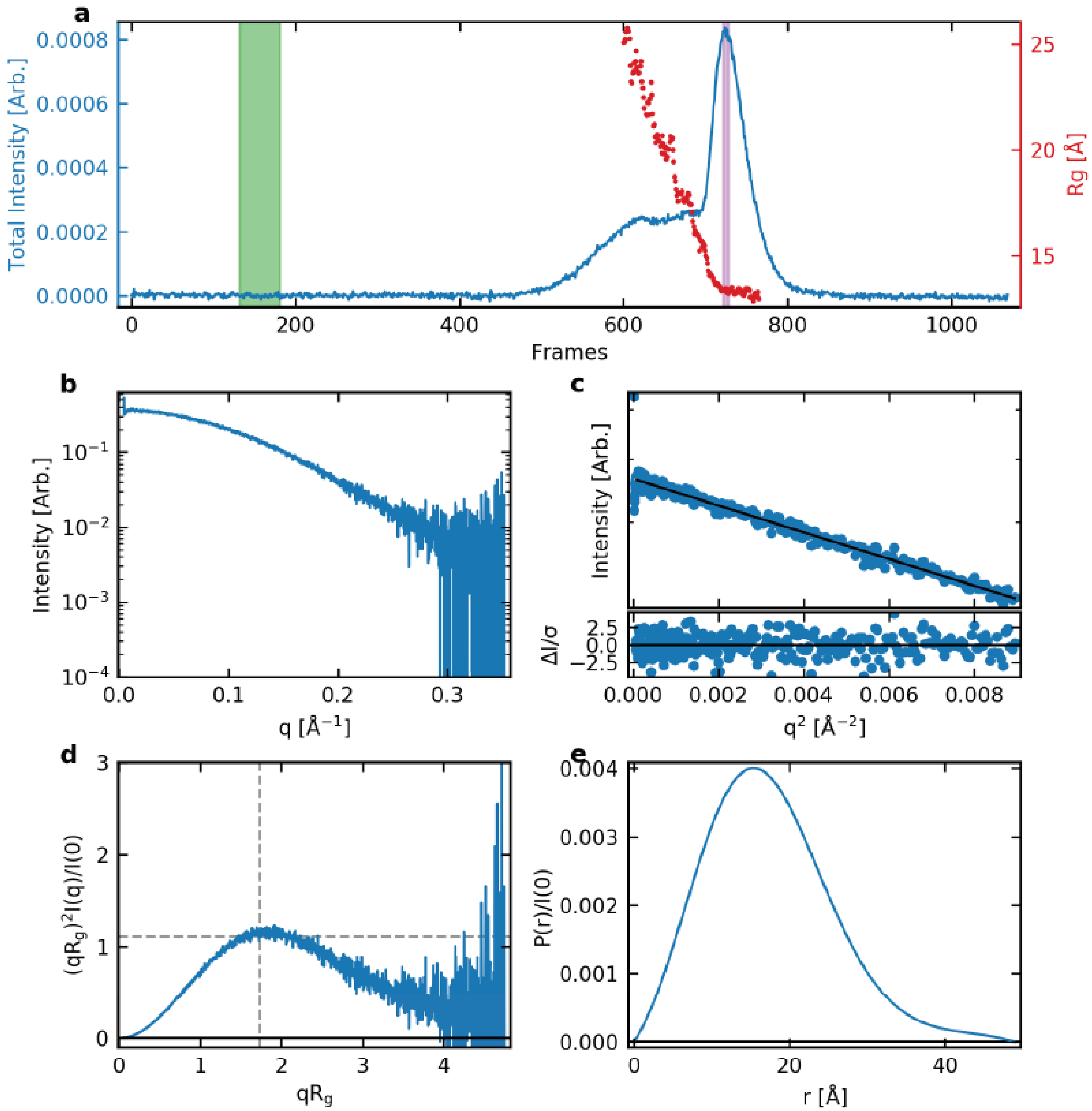

S17. SAXS data summary for 5I2V. a) Series intensity (blue, left axis) vs. frame, and, if available, Rg vs. frame (red, right axis). Green shaded regions are buffer regions, purple shaded regions are sample regions. b) Scattering profile(s) on a log-lin scale. c) Guinier fit(s) (top) and fit residuals (bottom). d) Normalized Kratky plot. Dashed lines show where a globular system would peak. e) P(r) function(s), normalized by I(0). Figure created in BioXTAS RAW v2.1.1.

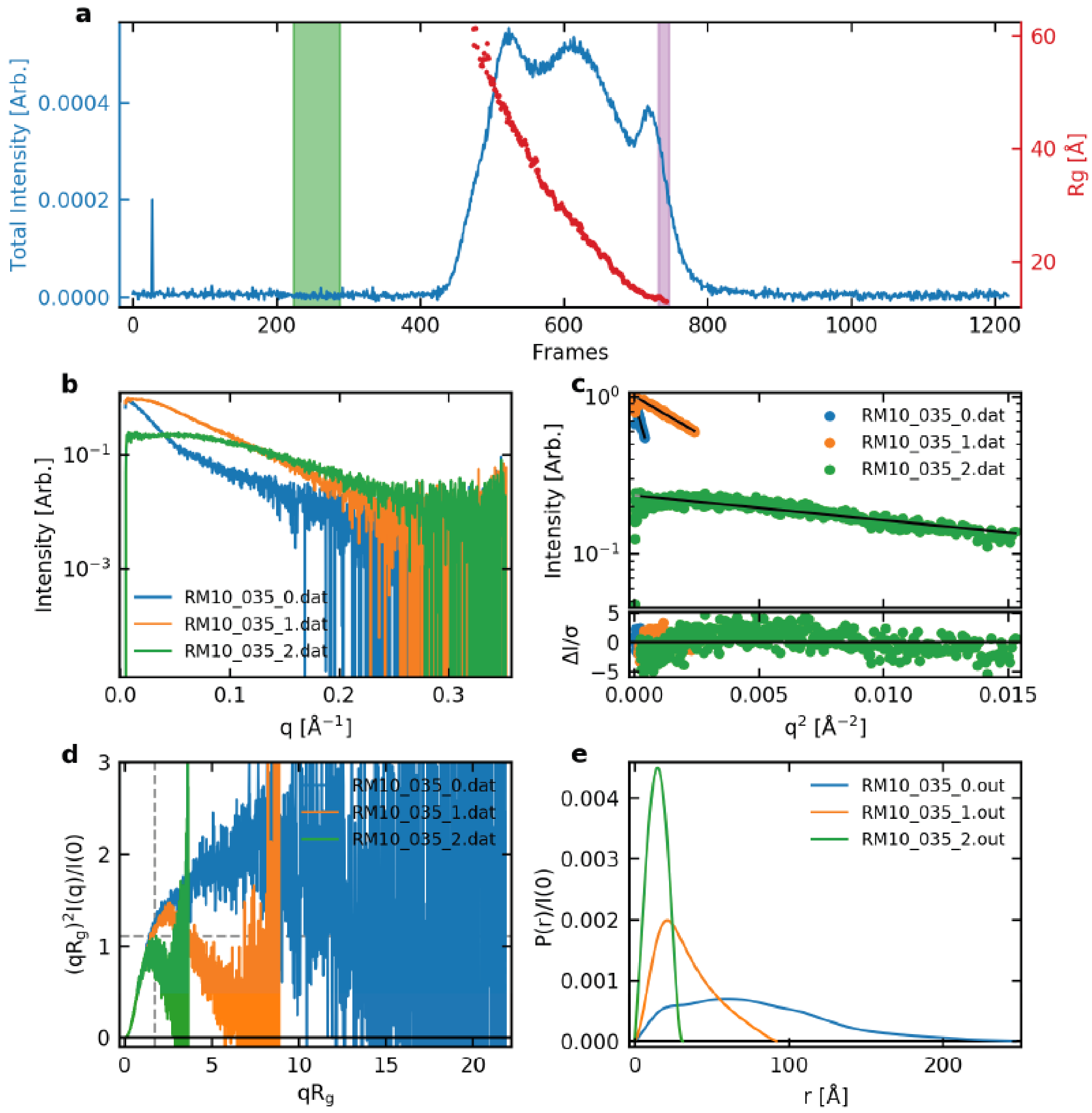

S18. SAXS data summary for 6GH0. a) Series intensity (blue, left axis) vs. frame, and, if available, Rg vs. frame (red, right axis). Green shaded regions are buffer regions, purple shaded regions are sample regions. b) Scattering profile(s) on a log-lin scale. c) Guinier fit(s) (top) and fit residuals (bottom). d) Normalized Kratky plot. Dashed lines show where a globular system would peak. e) P(r) function(s), normalized by I(0). Green (035_2), orange (031_1), and blue (031_0) species are the result of deconvolution using evolving factor analysis(1). The monomeric species, as determined by approximate molecular weight (Vc), is shown in green. Figure created in BioXTAS RAW v2.1.1.

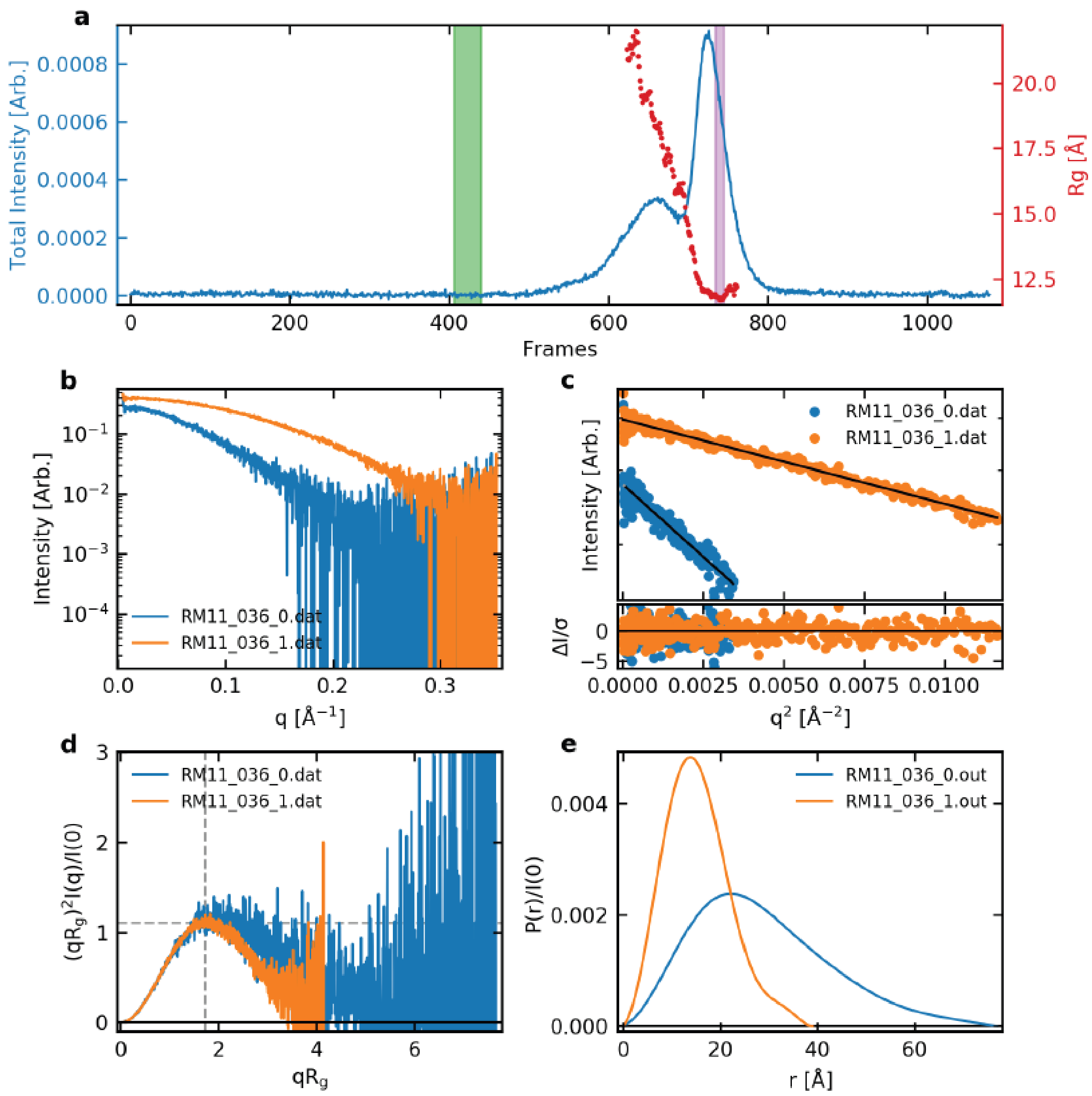

S19. SAXS data summary for 6L92. a) Series intensity (blue, left axis) vs. frame, and, if available, Rg vs. frame (red, right axis). Green shaded regions are buffer regions, purple shaded regions are sample regions. b) Scattering profile(s) on a log-lin scale. c) Guinier fit(s) (top) and fit residuals (bottom). d) Normalized Kratky plot. Dashed lines show where a globular system would peak. e) P(r) function(s), normalized by I(0). Orange (031_1), and blue (031_0) species are the result of deconvolution using evolving factor analysis(1). The monomeric species, as determined by approximate molecular weight (Vc), is shown in orange. Figure created in BioXTAS RAW v2.1.1.

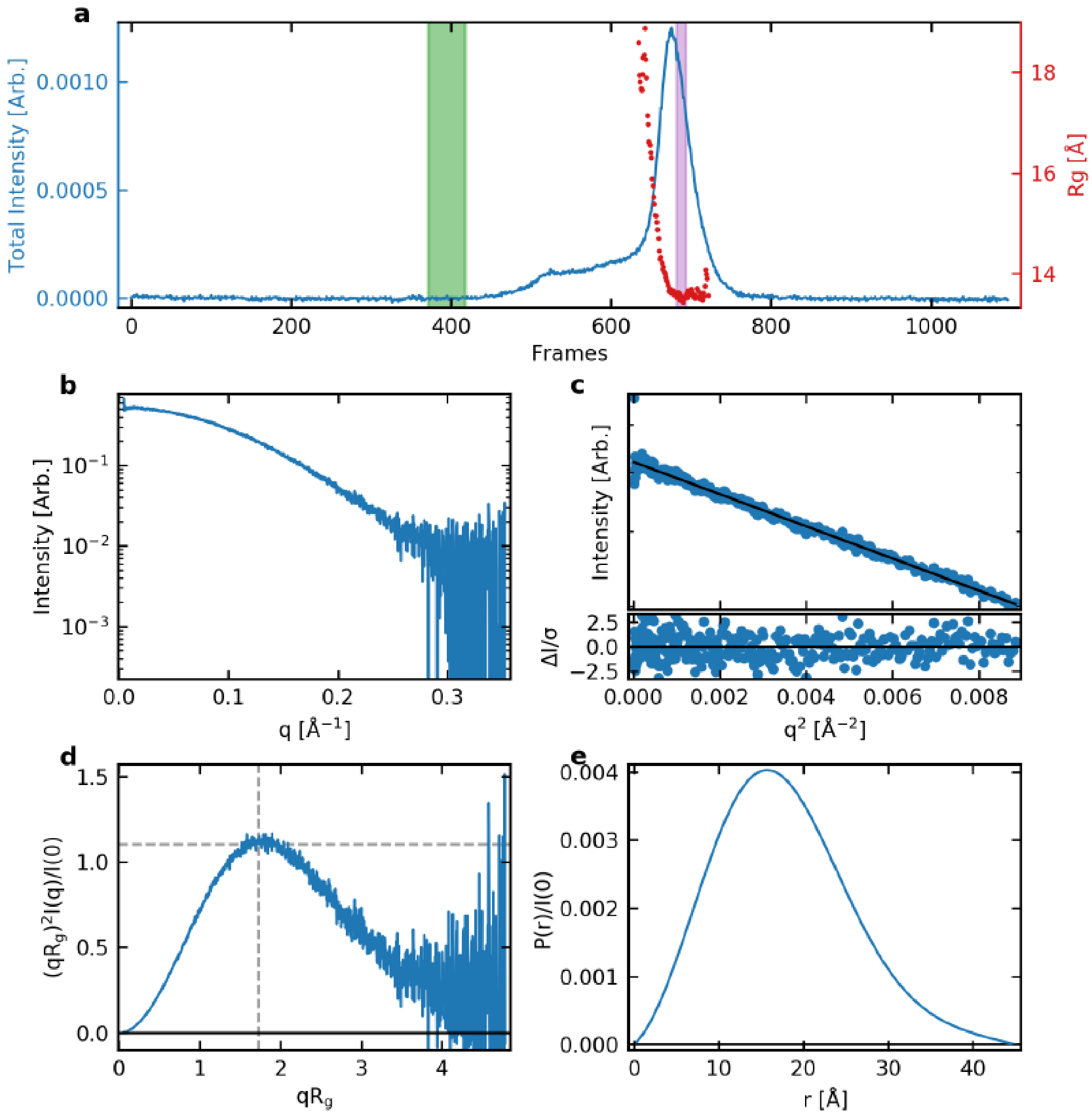

S20. SAXS data summary for 6NEB. a) Series intensity (blue, left axis) vs. frame, and, if available, Rg vs. frame (red, right axis). Green shaded regions are buffer regions, purple shaded regions are sample regions. b) Scattering profile(s) on a log-lin scale. c) Guinier fit(s) (top) and fit residuals (bottom). d) Normalized Kratky plot. Dashed lines show where a globular system would peak. e) P(r) function(s), normalized by I(0). Figure created in BioXTAS RAW v2.1.1.

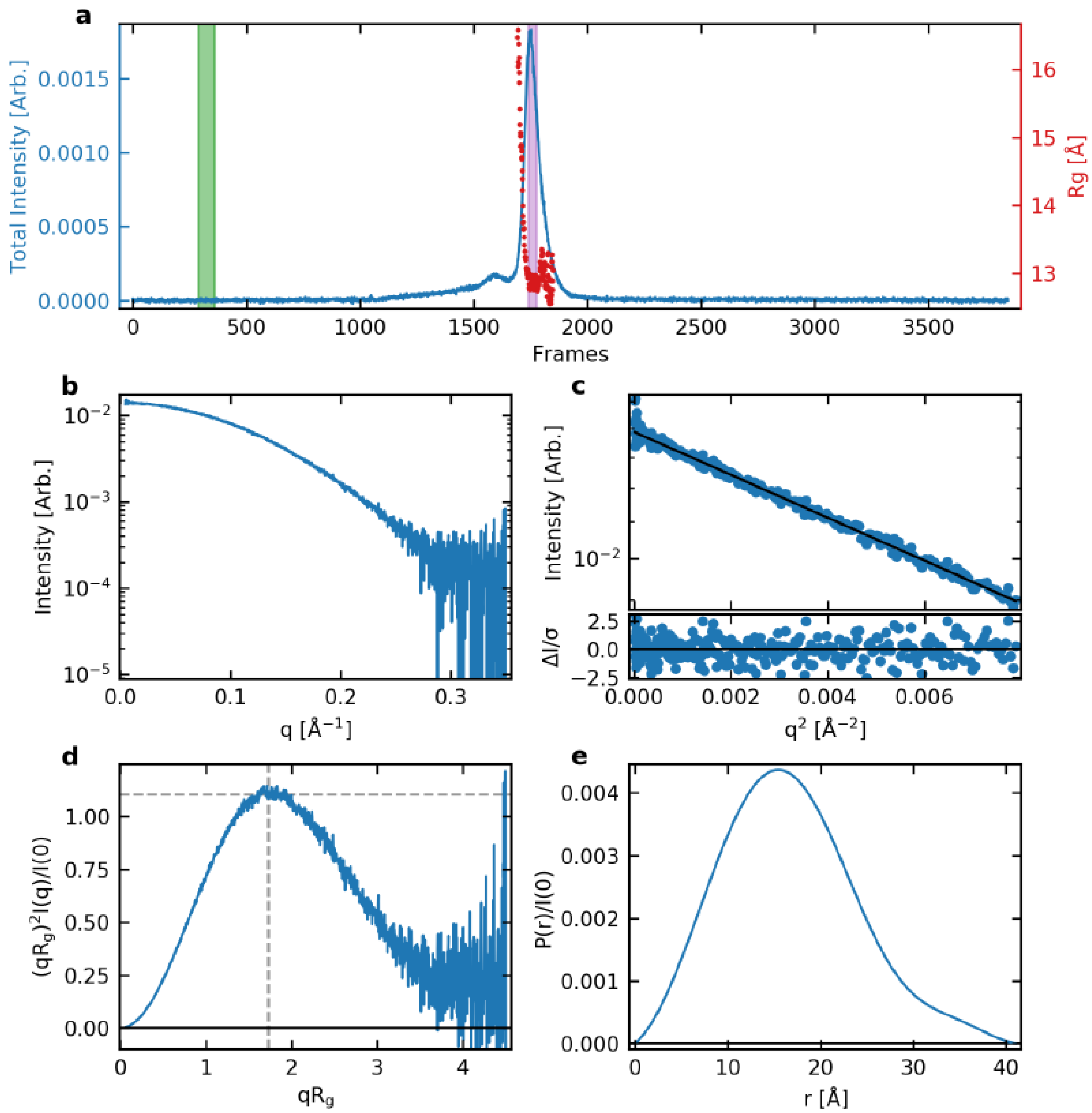

S21. SAXS data summary for 201D. a) Series intensity (blue, left axis) vs. frame, and, if available, Rg vs. frame (red, right axis). Green shaded regions are buffer regions, purple shaded regions are sample regions. b) Scattering profile(s) on a log-lin scale. c) Guinier fit(s) (top) and fit residuals (bottom). d) Normalized Kratky plot. Dashed lines show where a globular system would peak. e) P(r) function(s), normalized by I(0). Figure created in BioXTAS RAW v2.1.1.

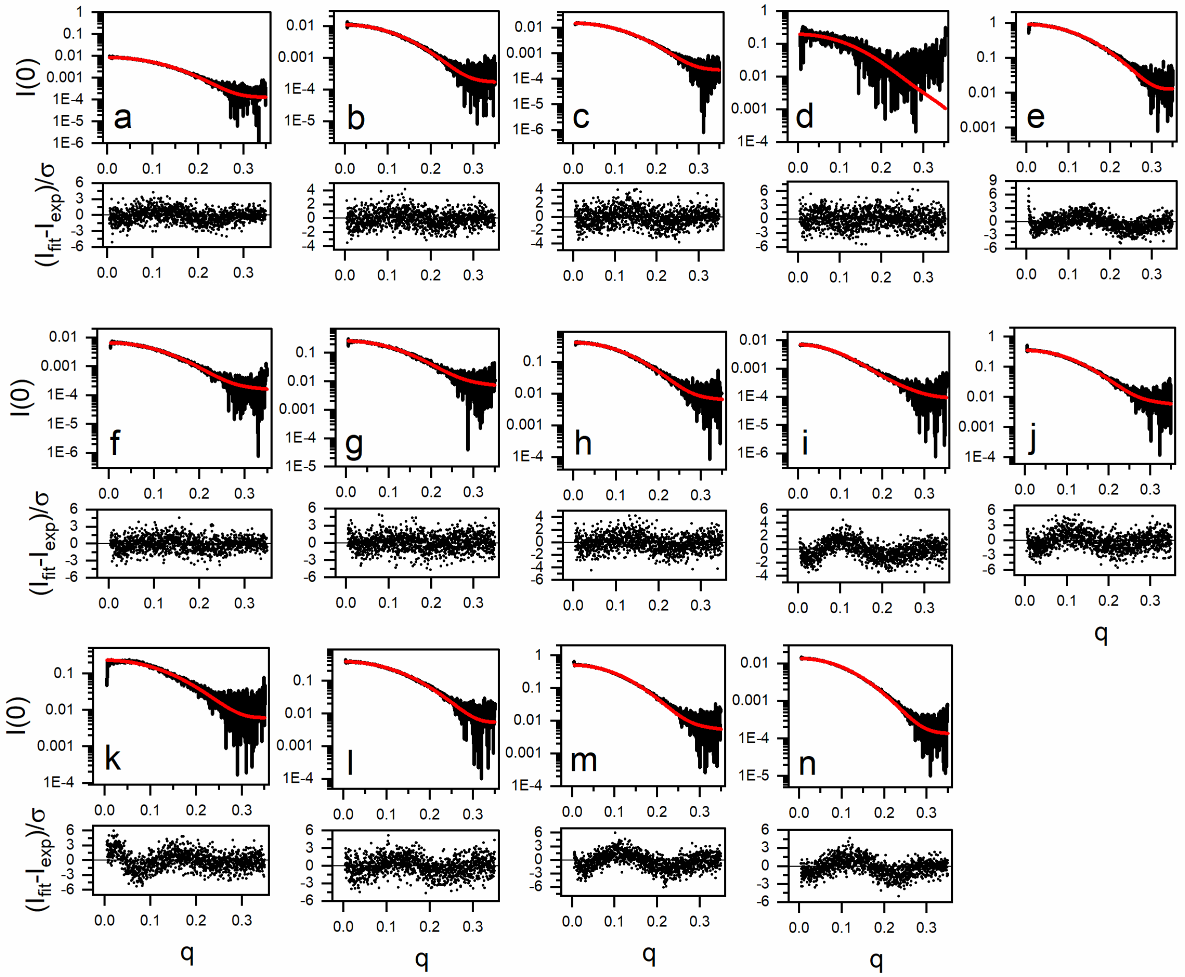

Figure S22. CRYSOL v2.8.3 fits of the 14 G4s from the PDB used in building the R_g regression. PDB ID's are: (a) 1XAV, (b) 2GKU, (c) 2JSL, (d) 2KQG, (e) 2KZD, (f) 2KZE, (g) 2LBY, (h) 2M27, (i) 5CMX, (j) 5I2V, (k) 6GH0, (l) 6L92, (m) 6NEB, (n) 201D. Tabulated results for the fits are given in Table S4.

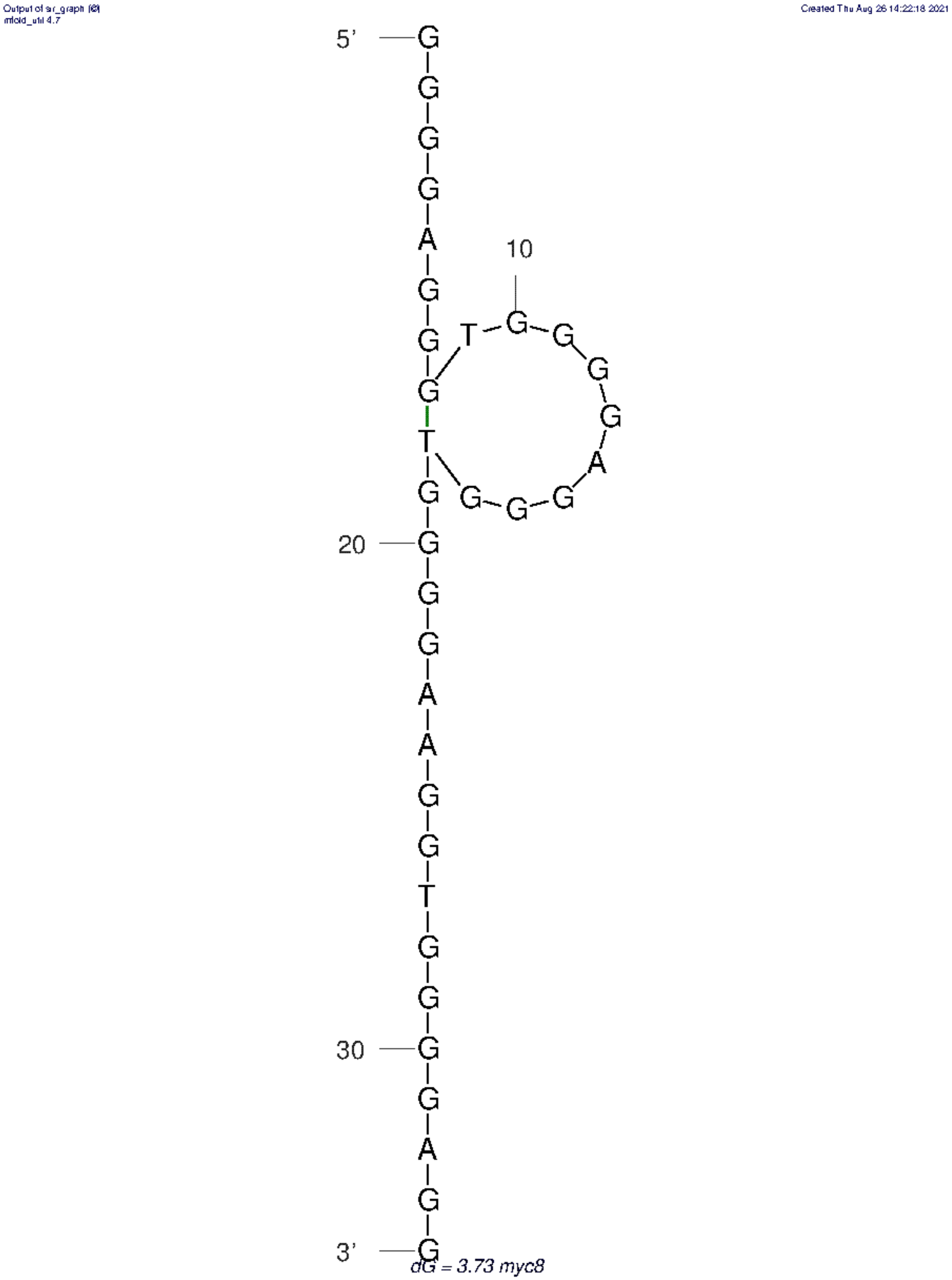

Figure S23. Mfold analysis of cMyc-8.

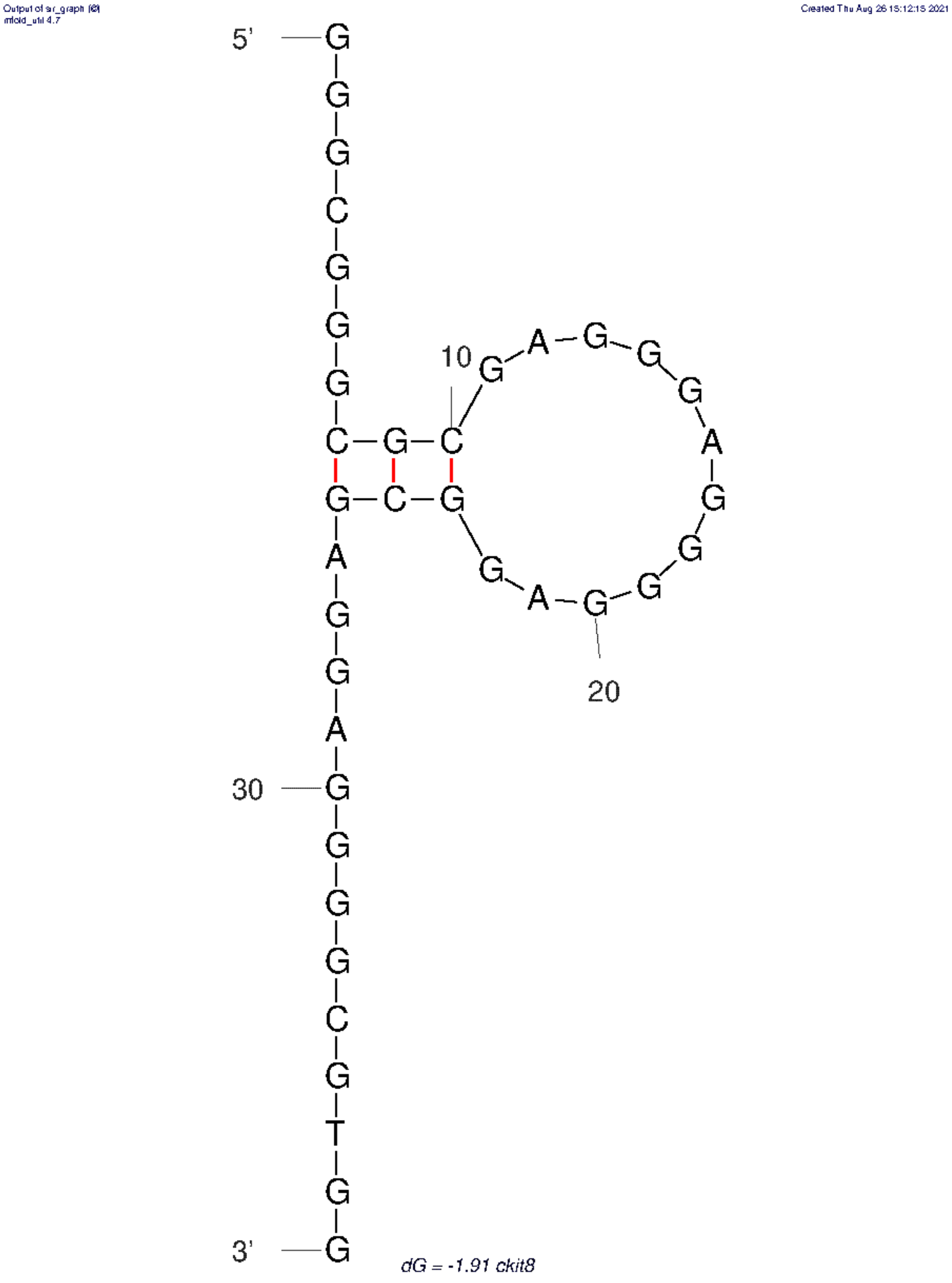

Figure S24. Mfold analysis of cKit-8.

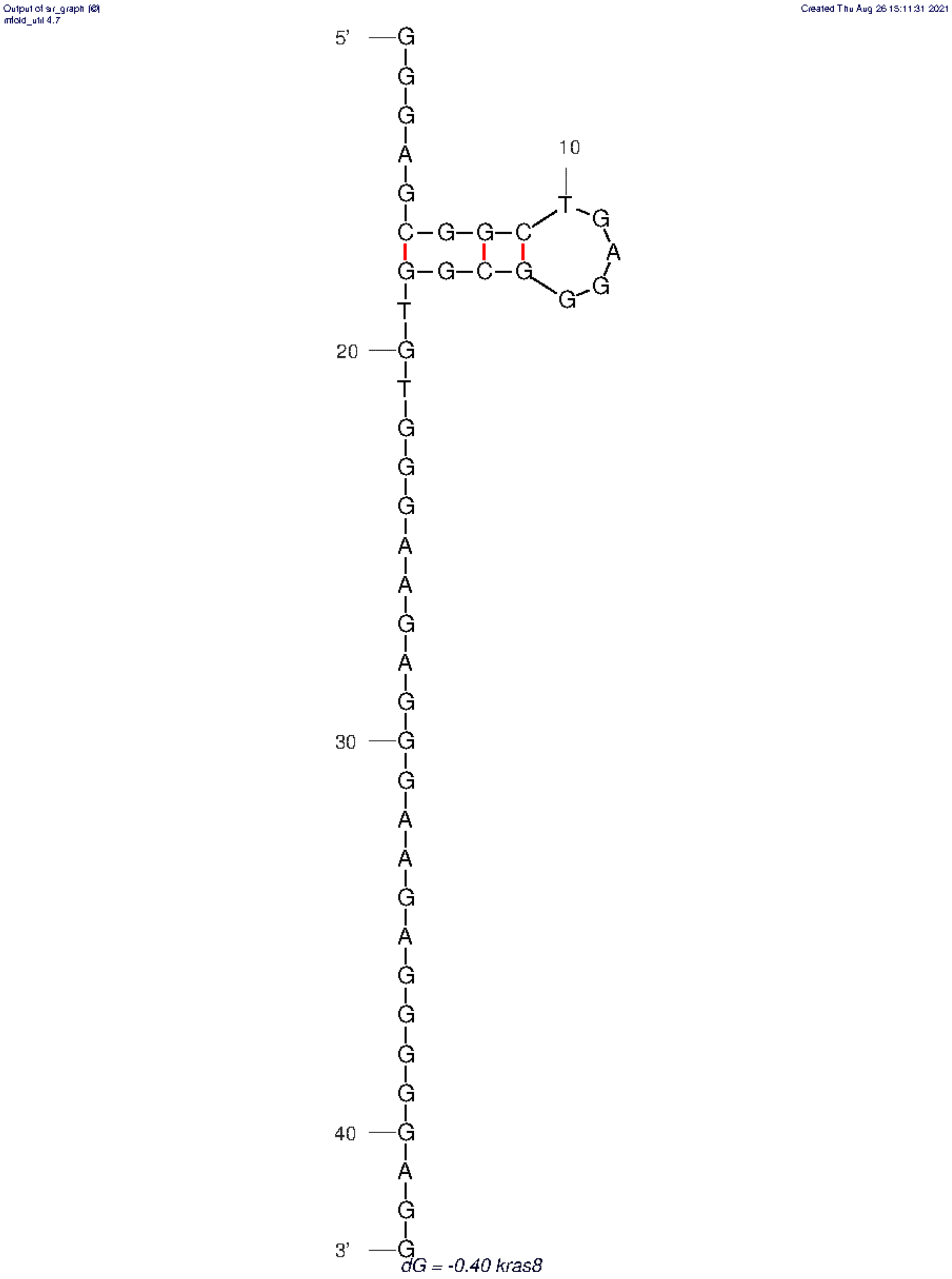

Figure S25. Mfold analysis of kRas-8.

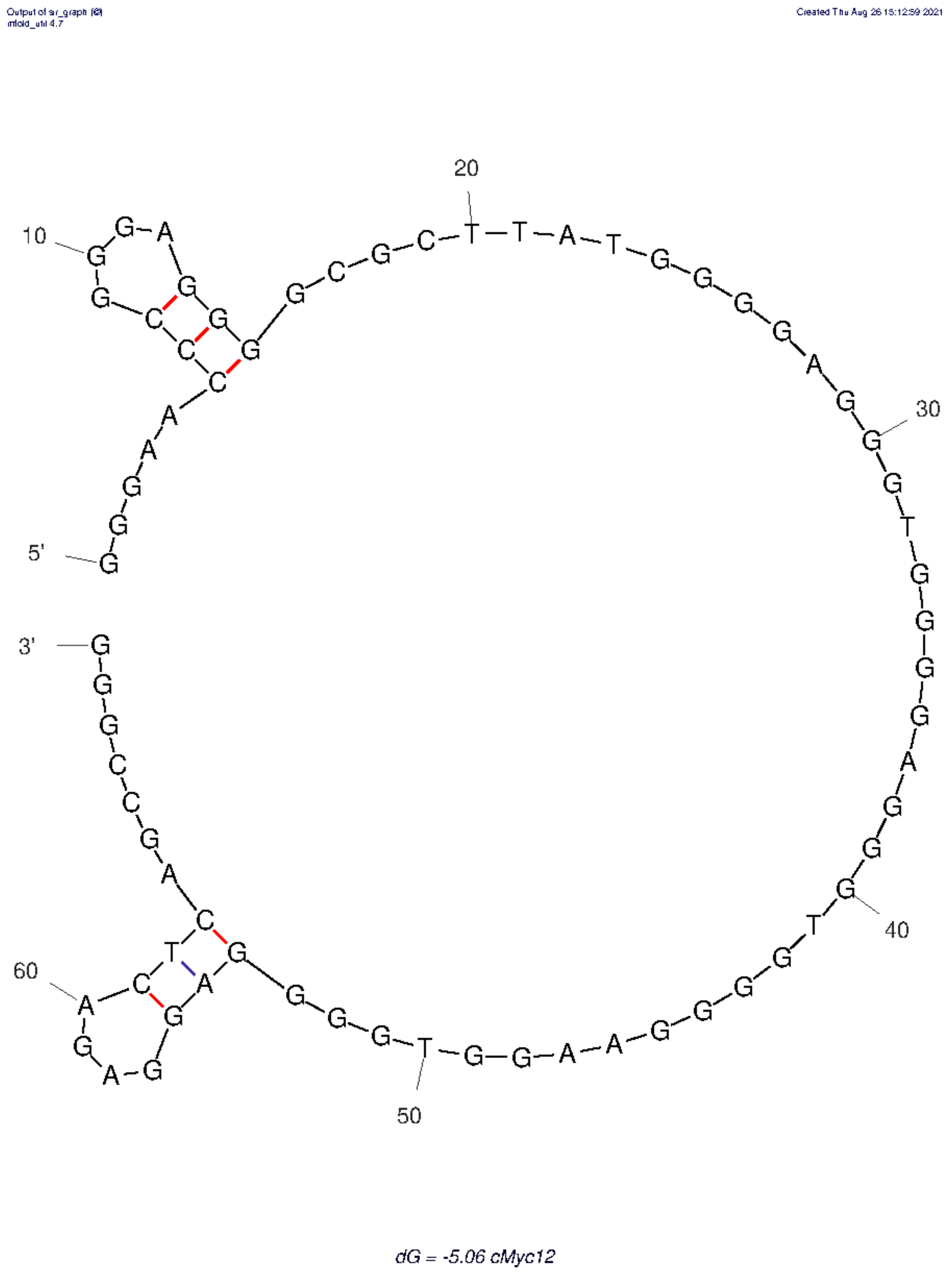

Figure S26. Mfold analysis of cMyc-12.

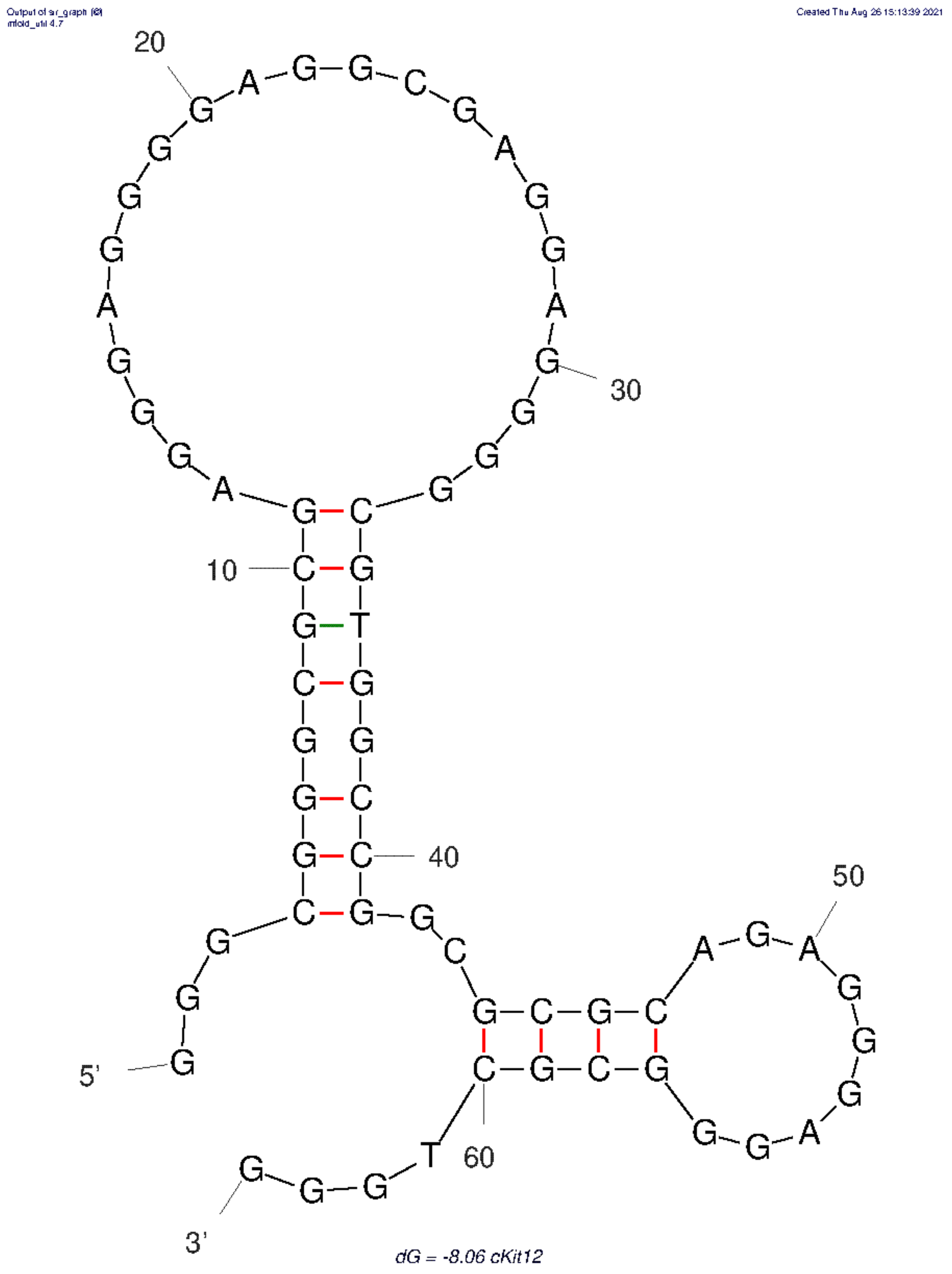

Figure S27. Mfold analysis of cKit-12.

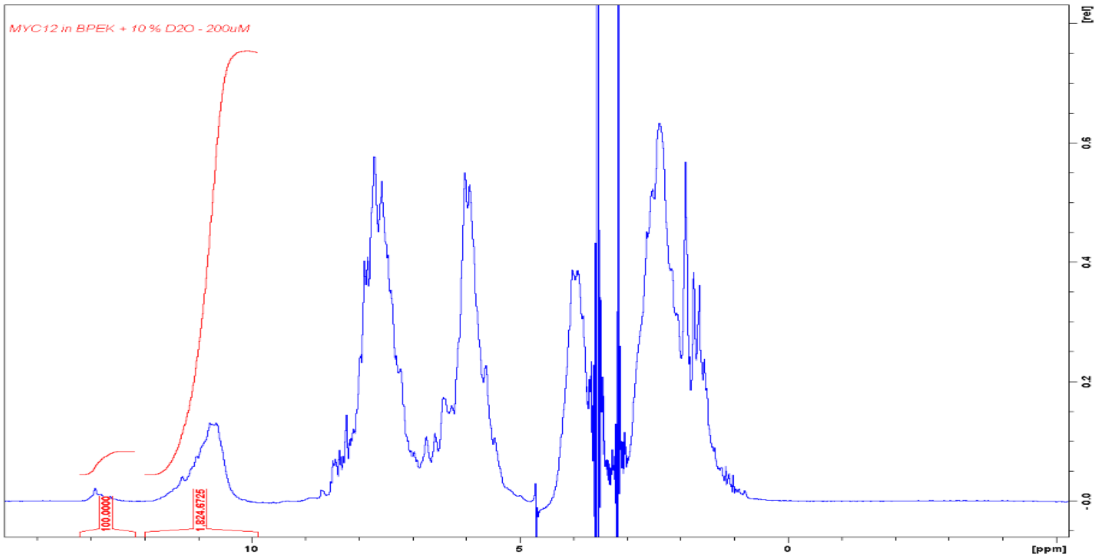

Figure S28. 1D proton NMR analysis of MYC-12. Red lines are integrations of the Watson-Crick and G-quadruplex imino proton regions, yielding a ratio of ~18:1 G-tetrad imino protons to duplex (Watson-Crick face) imino protons.

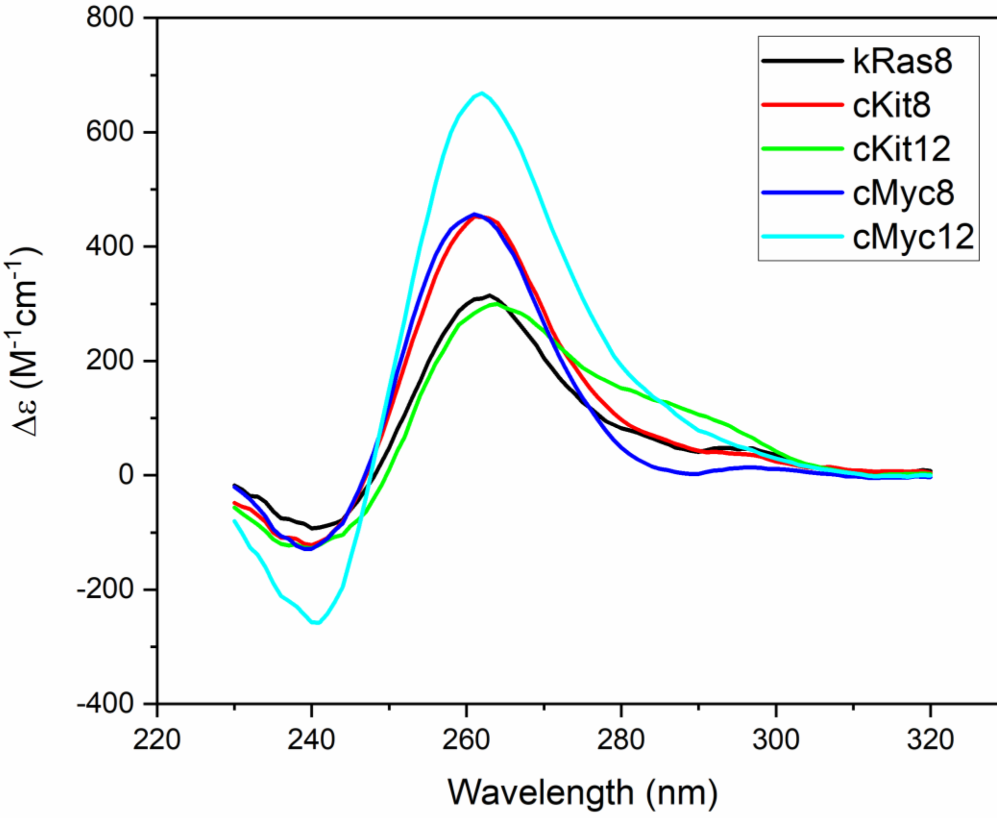

Figure S29. CD spectra of extended promoter sequences with 5' flanking nucleotides.

REFERENCES

1. Meisburger, S.P., Taylor, A.B., Khan, C.A., Zhang, S., Fitzpatrick, P.F. and Ando, N. (2016) Domain Movements upon Activation of Phenylalanine Hydroxylase Characterized by Crystallography and Chromatography-Coupled Small-Angle X-ray Scattering. *Journal of the American Chemical Society*, **138**, 6506-6516.
2. Dexheimer, T.S., Sun, D. and Hurley, L.H. (2006) Deconvoluting the structural and drug-recognition complexity of the G-quadruplex-forming region upstream of the bcl-2 P1 promoter. *J Am Chem Soc*, **128**, 5404-5415.
3. Guo, K., Gokhale, V., Hurley, L.H. and Sun, D. (2008) Intramolecularly folded G-quadruplex and i-motif structures in the proximal promoter of the vascular endothelial growth factor gene. *Nucleic Acids Res*, **36**, 4598-4608.
4. De Armond, R., Wood, S., Sun, D., Hurley, L.H. and Ebbinghaus, S.W. (2005) Evidence for the presence of a guanine quadruplex forming region within a polypurine tract of the hypoxia inducible factor 1alpha promoter. *Biochemistry*, **44**, 16341-16350.
5. Palumbo, S.L., Memmott, R.M., Uribe, D.J., Krotova-Khan, Y., Hurley, L.H. and Ebbinghaus, S.W. (2008) A novel G-quadruplex-forming GGA repeat region in the c-myb promoter is a critical regulator of promoter activity. *Nucleic Acids Res*, **36**, 1755-1769.
6. Qin, Y., Rezler, E.M., Gokhale, V., Sun, D. and Hurley, L.H. (2007) Characterization of the G-quadruplexes in the duplex nuclease hypersensitive element of the PDGF-A promoter and modulation of PDGF-A promoter activity by TMPyP4. *Nucleic Acids Res*, **35**, 7698-7713.
7. Mitchell, T., Ramos-Montoya, A., Di Antonio, M., Murat, P., Ohnmacht, S., Micco, M., Jurmeister, S., Fryer, L., Balasubramanian, S., Neidle, S. *et al.* (2013) Downregulation of androgen receptor transcription by promoter g-quadruplex stabilization as a potential alternative treatment for castrate-resistant prostate cancer. *Biochemistry*, **52**, 1429-1436.
8. Tong, X., Lan, W., Zhang, X., Wu, H., Liu, M. and Cao, C. (2011) Solution structure of all parallel G-quadruplex formed by the oncogene RET promoter sequence. *Nucleic Acids Res*, **39**, 6753-6763.
9. Greco, M.L., Kotar, A., Rigo, R., Cristofari, C., Plavec, J. and Sissi, C. (2017) Coexistence of two main folded G-quadruplexes within a single G-rich domain in the EGFR promoter. *Nucleic Acids Res*, **45**, 10132-10142.
10. Sengar, A., Vandana, J.J., Chambers, V.S., Di Antonio, M., Winnerdy, F.R., Balasubramanian, S. and Phan, A.T. (2019) Structure of a (3+1) hybrid G-quadruplex in the PARP1 promoter. *Nucleic Acids Res*, **47**, 1564-1572.
11. Wang, J.M., Huang, F.C., Kuo, M.H., Wang, Z.F., Tseng, T.Y., Chang, L.C., Yen, S.J., Chang, T.C. and Lin, J.J. (2014) Inhibition of cancer cell migration and invasion through suppressing the Wnt1-mediating signal pathway by G-quadruplex structure stabilizers. *J Biol Chem*, **289**, 14612-14623.
12. Kuo, M.H., Wang, Z.F., Tseng, T.Y., Li, M.H., Hsu, S.T., Lin, J.J. and Chang, T.C. (2015) Conformational transition of a hairpin structure to G-quadruplex within the WNT1 gene promoter. *J Am Chem Soc*, **137**, 210-218.
13. Yafe, A., Etzioni, S., Weisman-Shomer, P. and Fry, M. (2005) Formation and properties of hairpin and tetraplex structures of guanine-rich regulatory sequences of muscle-specific genes. *Nucleic Acids Res*, **33**, 2887-2900.
14. Huang, M.C., Chu, I.T., Wang, Z.F., Lin, S., Chang, T.C. and Chen, C.T. (2018) A G-Quadruplex Structure in the Promoter Region of CLIC4 Functions as a Regulatory Element for Gene Expression. *Int J Mol Sci*, **19**.
15. Zhu, J., Fleming, A.M. and Burrows, C.J. (2018) The RAD17 Promoter Sequence Contains a Potential Tail-Dependent G-Quadruplex That Downregulates Gene Expression upon Oxidative Modification. *ACS Chem Biol*, **13**, 2577-2584.

16. Redstone, S.C.J., Fleming, A.M. and Burrows, C.J. (2019) Oxidative Modification of the Potential G-Quadruplex Sequence in the PCNA Gene Promoter Can Turn on Transcription. *Chem Res Toxicol*, **32**, 437-446.
17. Huang, W., Smaldino, P.J., Zhang, Q., Miller, L.D., Cao, P., Stadelman, K., Wan, M., Giri, B., Lei, M., Nagamine, Y. *et al.* (2012) Yin Yang 1 contains G-quadruplex structures in its promoter and 5'-UTR and its expression is modulated by G4 resolvase 1. *Nucleic Acids Res*, **40**, 1033-1049.
18. Ohnmacht, S.A., Micco, M., Petrucci, V., Todd, A.K., Reszka, A.P., Gunaratnam, M., Carvalho, M.A., Zloh, M. and Neidle, S. (2012) Sequences in the HSP90 promoter form G-quadruplex structures with selectivity for disubstituted phenyl bis-oxazole derivatives. *Bioorg Med Chem Lett*, **22**, 5930-5935.
19. Wei, P.C., Wang, Z.F., Lo, W.T., Su, M.I., Shew, J.Y., Chang, T.C. and Lee, W.H. (2013) A cis-element with mixed G-quadruplex structure of NPGPx promoter is essential for nucleolin-mediated transactivation on non-targeting siRNA stress. *Nucleic Acids Res*, **41**, 1533-1543.
20. Zhao, Y. and Uhler, J.P. (2018) Identification of a G-quadruplex forming sequence in the promoter of UCP1. *Acta Biochim Biophys Sin (Shanghai)*, **50**, 718-722.
21. Stevens, A.J. and Kennedy, M.A. (2017) Structural Analysis of G-Quadruplex Formation at the Human MEST Promoter. *PLoS One*, **12**, e0169433.
22. Purohit, G., Mukherjee, A.K., Sharma, S. and Chowdhury, S. (2018) Extratelomeric Binding of the Telomere Binding Protein TRF2 at the PCGF3 Promoter Is G-Quadruplex Motif-Dependent. *Biochemistry*, **57**, 2317-2324.
23. Farhath, M.M., Thompson, M., Ray, S., Sewell, A., Balci, H. and Basu, S. (2015) G-Quadruplex-Enabling Sequence within the Human Tyrosine Hydroxylase Promoter Differentially Regulates Transcription. *Biochemistry*, **54**, 5533-5545.
24. Schlag, K., Steinhilber, D., Karas, M. and Sorg, B.L. (2020) Analysis of proximal ALOX5 promoter binding proteins by quantitative proteomics. *FEBS J*, **287**, 4481-4499.
25. Salvati, E., Zizza, P., Rizzo, A., Iachettini, S., Cingolani, C., D'Angelo, C., Porru, M., Randazzo, A., Pagano, B., Novellino, E. *et al.* (2014) Evidence for G-quadruplex in the promoter of vegfr-2 and its targeting to inhibit tumor angiogenesis. *Nucleic Acids Res*, **42**, 2945-2957.
26. Basundra, R., Kumar, A., Amrane, S., Verma, A., Phan, A.T. and Chowdhury, S. (2010) A novel G-quadruplex motif modulates promoter activity of human thymidine kinase 1. *FEBS J*, **277**, 4254-4264.
27. Waller, Z.A., Howell, L.A., Macdonald, C.J., O'Connell, M.A. and Searcey, M. (2014) Identification and characterisation of a G-quadruplex forming sequence in the promoter region of nuclear factor (erythroid-derived 2)-like 2 (Nrf2). *Biochem Biophys Res Commun*, **447**, 128-132.
28. Yan, J., Zhao, D., Dong, L., Pan, S., Hao, F. and Guan, Y. (2017) A novel G-quadruplex motif in the Human MET promoter region. *Biosci Rep*, **37**.
29. Mathad, R.I., Hatzakis, E., Dai, J. and Yang, D. (2011) c-MYC promoter G-quadruplex formed at the 5'-end of NHE III1 element: insights into biological relevance and parallel-stranded G-quadruplex stability. *Nucleic Acids Res*, **39**, 9023-9033.
30. Fernando, H., Reszka, A.P., Huppert, J., Ladame, S., Rankin, S., Venkitaraman, A.R., Neidle, S. and Balasubramanian, S. (2006) A conserved quadruplex motif located in a transcription activation site of the human c-kit oncogene. *Biochemistry*, **45**, 7854-7860.
31. Zhang, L., Tan, W., Zhou, J., Xu, M. and Yuan, G. (2017) Investigation of G-quadruplex formation in the FGFR2 promoter region and its transcriptional regulation by liensinine. *Biochim Biophys Acta Gen Subj*, **1861**, 884-891.
32. Kirby, N., Cowieson, N., Hawley, A.M., Mudie, S.T., McGillivray, D.J., Kusel, M., Samardzic-Boban, V. and Ryan, T.M. (2016) Improved radiation dose efficiency in solution SAXS using a sheath flow sample environment. *Acta Crystallogr D Struct Biol*, **72**, 1254-1266.

33. Hopkins, J.B., Gillilan, R.E. and Skou, S. (2017) BioXTAS RAW: improvements to a free open-source program for small-angle X-ray scattering data reduction and analysis. *J Appl Crystallogr*, **50**, 1545-1553.
34. Semenyuk, A.V. and Svergun, D.I. (1991) GNOM– a program package for small-angle scattering data processing. *Journal of Applied Crystallography*, **24**, 537-540.
35. Franke, D. and Svergun, D.I. (2009) DAMMIF, a program for rapid ab-initio shape determination in small-angle scattering. *J Appl Crystallogr*, **42**, 342-346.
36. Volkov, V.V. and Svergun, D.I. (2003) Uniqueness of ab initio shape determination in small-angle scattering. *Journal of Applied Crystallography*, **36**, 860-864.
37. Petoukhov, M.V., Franke, D., Shkumatov, A.V., Tria, G., Kikhney, A.G., Gajda, M., Gorba, C., Mertens, H.D., Konarev, P.V. and Svergun, D.I. (2012) New developments in the ATSAS program package for small-angle scattering data analysis. *J Appl Crystallogr*, **45**, 342-350.
38. Svergun, D.I. (1999) Restoring low resolution structure of biological macromolecules from solution scattering using simulated annealing. *Biophys J*, **76**, 2879-2886.
39. Svergun, D., Barberato, C. and Koch, M.H.J. (1995) CRYSOL - a Program to Evaluate X-ray Solution Scattering of Biological Macromolecules from Atomic Coordinates. *Journal of Applied Crystallography*, **28**, 768-773.
